# A gene signature consisting of ubiquitin ligases and deubiquitinating enzymes of SKP2 is associated with clinical outcome in breast cancer

**DOI:** 10.1038/s41598-022-06451-w

**Published:** 2022-02-15

**Authors:** Lon W. R. Fong, Jangsoon Lee, Hui-Kuan Lin, Naoto T. Ueno, Shuxing Zhang

**Affiliations:** 1 Intelligent Molecular Discovery Laboratory, Department of Experimental Therapeutics, The University of Texas MD Anderson Cancer Center, Houston, TX 77054 USA; 2 Department of Breast Medical Oncology, The University of Texas MD Anderson Cancer Center, Houston, TX 77030 USA; 3 Department of Cancer Biology, Wake Forest University Baptist Medical Center, Winston-Salem, NC 27101 USA

**Keywords:** Computational biology and bioinformatics, Cancer, Breast cancer, Cancer genetics, Cancer genomics, Tumour biomarkers

## Abstract

The ubiquitination of SKP2, an oncoprotein, is controlled by its E3 ligases, including APC/C^FZR1^ and deubiquitinases such as USP10. We identified a two-gene signature for the ubiquitination of SKP2, consisting of the copy number of FZR1 compared to the copy number of USP10. The signature reflects the level of SKP2 activity, stratifying BC patients into two groups with significantly different protein levels of SKP2 ubiquitination substrate p27 (*t*-test *p* < 0.01) and recapitulating the expression patterns of SKP2 between tumor and normal tissue (Spearman’s *ρ* = 0.39.) The signature is also highly associated with clinical outcome in luminal BC but not other subtypes, characterizing patients into two groups with significantly different overall survival times (log-rank *p* = 0.006). In addition, it is dramatically associated with tumor grade (Chi-squared *p* = 6.7 × 10^−3^), stage (Chi-squared *p* = 1.6 × 10^−11^), and the number of positive lymph nodes (negative binomial regression coefficient *p* = 2.0 × 10^−3^). Our study provides a rationale for targeting the SKP2 ubiquitination pathway in luminal BC and for further investigation of the use of ubiquitinase/deubiquitinase genes as prognosis and treatment biomarkers.

## Introduction

S-phase kinase-associated protein 2 (SKP2) is part of the Skp1–Cullin–F-box (SCF) complex, an E3 ubiquitin ligase. The main targets of SKP2 include proteins such as p27 and p21 that block progression through the cell cycle, specifically S phase, G2 phase, and the beginning of the M phase^1–12^. By ubiquitinating these proteins, the SCF complex marks them for degradation by the 26S proteasome. As many of SKP2’s targets halt the cell-cycle progression and are therefore tumor suppressors, SKP2 has an oncogenic role in many cancers^13–16^. Accordingly, recent studies have shown SKP2 is a promising target for cancer therapy, and a few studies, including our own, have demonstrated successful pharmacological inhibition of SKP2 in preclinical cancer models^17–19^. Since the oncogenic role of SKP2 in breast cancer (BC) in particular is well established^20–23^, here we focus our study on this disease.

**Figure 1:**
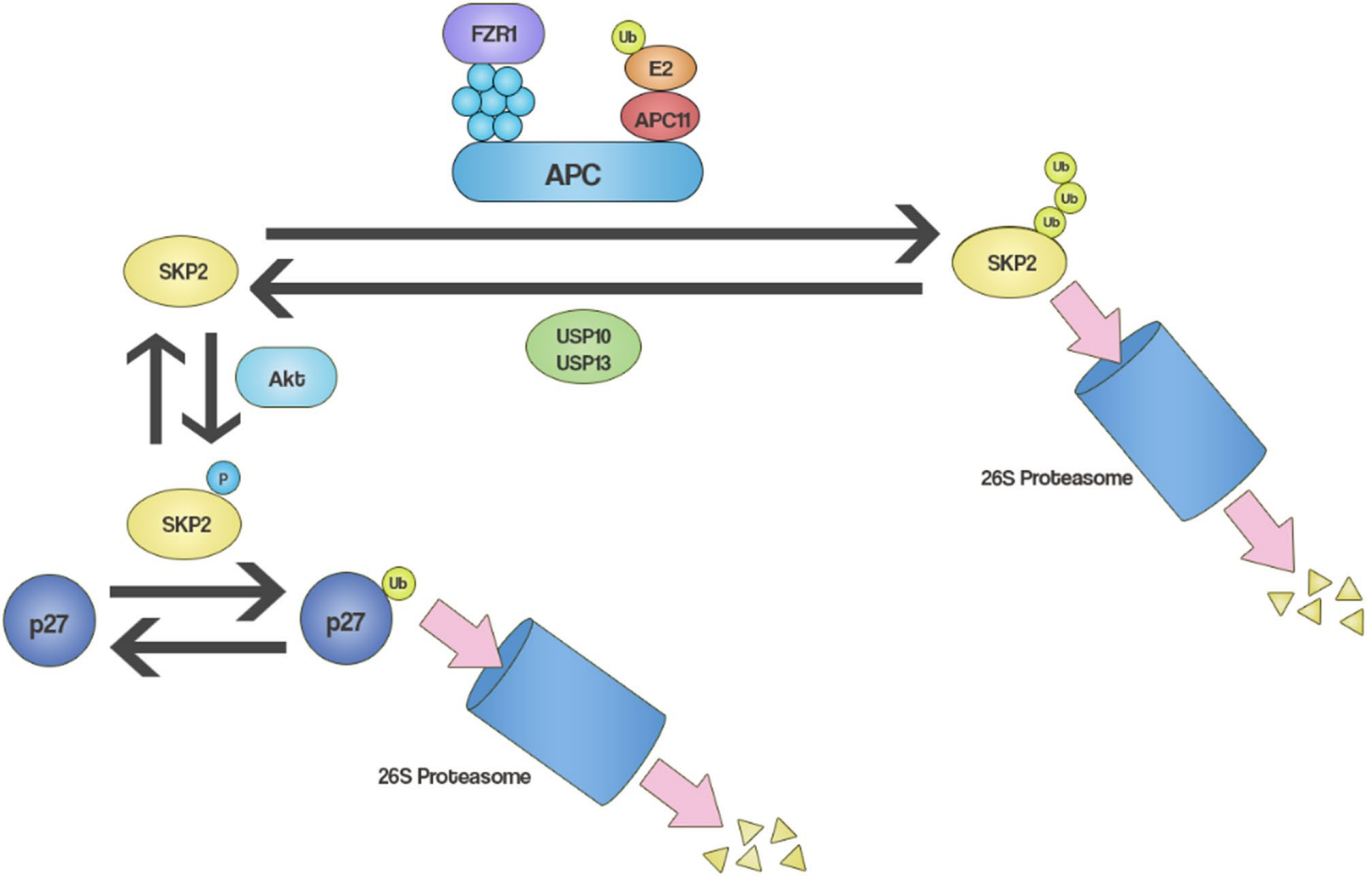
A schematic illustration of the regulation of SKP2 ubiquitination by APC/C (E3 ubiquitin ligase) and USP10/USP13 (deubiquitinating enzymes), and SKP2-mediated ubiquitination of p27.

Like many proteins, SKP2 can itself be ubiquitinated and thus marked for proteasomal degradation. The ubiquitin ligase that targets SKP2 is the anaphase-promoting complex/cyclosome (APC/C), with fizzy-related protein homolog (FZR1) the subunit that confers specificity for SKP2 (APC/C^FZR1^)^24,25^. Furthermore, SKP2 has also been shown to be deubiquitinated by ubiquitin-specific peptidase 13 (USP13) and ubiquitin-specific peptidase 10 (USP10) in different model systems^26–28^. Since these proteins control the ubiquitination of SKP2 and thereby SKP2 protein levels (Fig. 1), we hypothesized that a gene signature consisting of FZR1 and at least one of USP10 and USP13 could be used as an approximation for SKP2 ubiquitination and protein levels in BC.

Furthermore, the use of genomic aberrations as prognostic and predictive biomarkers is already standard clinical practice in BC, particularly the use of HER2 amplification status for both prognosis and to predict response to anti-HER2 therapy. We therefore further hypothesized that this gene signature could also be prognostic in BC. Finally, for the signature we used copy number rather than mRNA levels or mutations. This is for two reasons: first, copy number is more stable than mRNA levels, as transcription can be changed in response to external stimuli. Second, copy number was shown in a pan-cancer analysis by Smith and Sheltzer to be more prognostic than mutations in cancer driver genes^29^. Our proposed signatures take into account only two genes: one ubiquitin ligase component (FZR1) and one deubiquitinase (USP10 or USP13). The advantage of this signature is that it allows for the stratification of patients into three groups easily distinguished by biological characteristics readily apparent from the signature (Table 1), a feature decided upon with clinical decision-making in mind.

**Table 1:** A summary of our SKP2 ubiquitination signature.

SKP2 ubiquitination signature group	FZR1 copy number vs USP10/USP13 copy number	Expected SKP2 protein levels	Expected p27 protein levels
High	FZR1 > USP10/USP13	Low	High
Intermediate	FZR1 = USP10/USP13	Intermediate	Intermediate
Low	FZR1 < USP10/USP13	High	Low

Neither USP13 nor USP10 has yet been shown either in vitro or in vivo to deubiquitinate SKP2 in BC specifically; so far, USP10 has only been shown to deubiquitinate SKP2 in chronic myelogenous leukemia and neointima formation in vascular smooth-muscle tissue^27,28^, and evidence supporting the role of USP13 as a deubiquitinase of SKP2 has only been obtained from in vitro biochemical assays and in HeLa cells^26^. Our study therefore provides a rationale to further investigate the role of these proteins in BC. If the hypothesis is verified in vitro or in vivo, it would indicate that USP13 or USP10 may be a promising therapeutic target in BC. It also raises the possibility of using a bioinformatic approach as we have taken here to identify potential deubiquitinases of other proteins in other cancers.

In the present study, our goal is to identify gene signatures that could adequately represent target protein (SKP2) levels relevant in BC patient survival and clinical treatment. To this end, we first examined genomic and proteomic data from the Cancer Genome Atlas (TCGA). Next, we sought to provide evidence that our signature was acting through the proposed mechanism. Finally, we tested our hypothesis that the gene signature could be prognostic in BC by determining the extent to which our gene signature was associated with clinical outcome.

## Results

### Identification and validation of a gene signature to approximate SKP2 ubiquitination and protein levels

Two ubiquitin-specific peptidases (USPs), USP10 and USP13, have been shown to deubiquitinate SKP2, but neither of them has been reported with this function in BC specifically. Meanwhile, APC/C^FZR1^ is the only E3 ubiquitin ligase experimentally verified to ubiquitinate SKP2, and it has been demonstrated to do so in BC cells^30^. We therefore set out to identify whether one of these USPs, or possibly both, deubiquitinates SKP2 in BC. To do this, we created two separate gene signatures putatively representing levels of SKP2 ubiquitination: one consisting of FZR1 and USP10, and the other consisting of FZR1 and USP13. We then used each of these gene signatures to stratify patient samples from TCGA into two groups: patients with higher FZR1 copy number than the particular USP (USP10 or USP13) were categorized as “high SKP2 ubiquitination”, and patients with higher copy numbers of the particular USP than FZR1 were categorized as “low SKP2 ubiquitination” (Table 1). Copy-number comparisons were performed using the copy-number levels provided by cBioPortal, which assigns a level (− 2, − 1, 0, 1, 2) to a gene based on its putative copy-number alteration (deep deletion, shallow deletion, diploidy, low-level gain, and high-level amplification, respectively). For example, a patient with a USP10 or USP13 copy-number level of 0 and an FZR copy-number level of -1 would be classified as “low SKP2 ubiquitination”.

**Figure 2:**
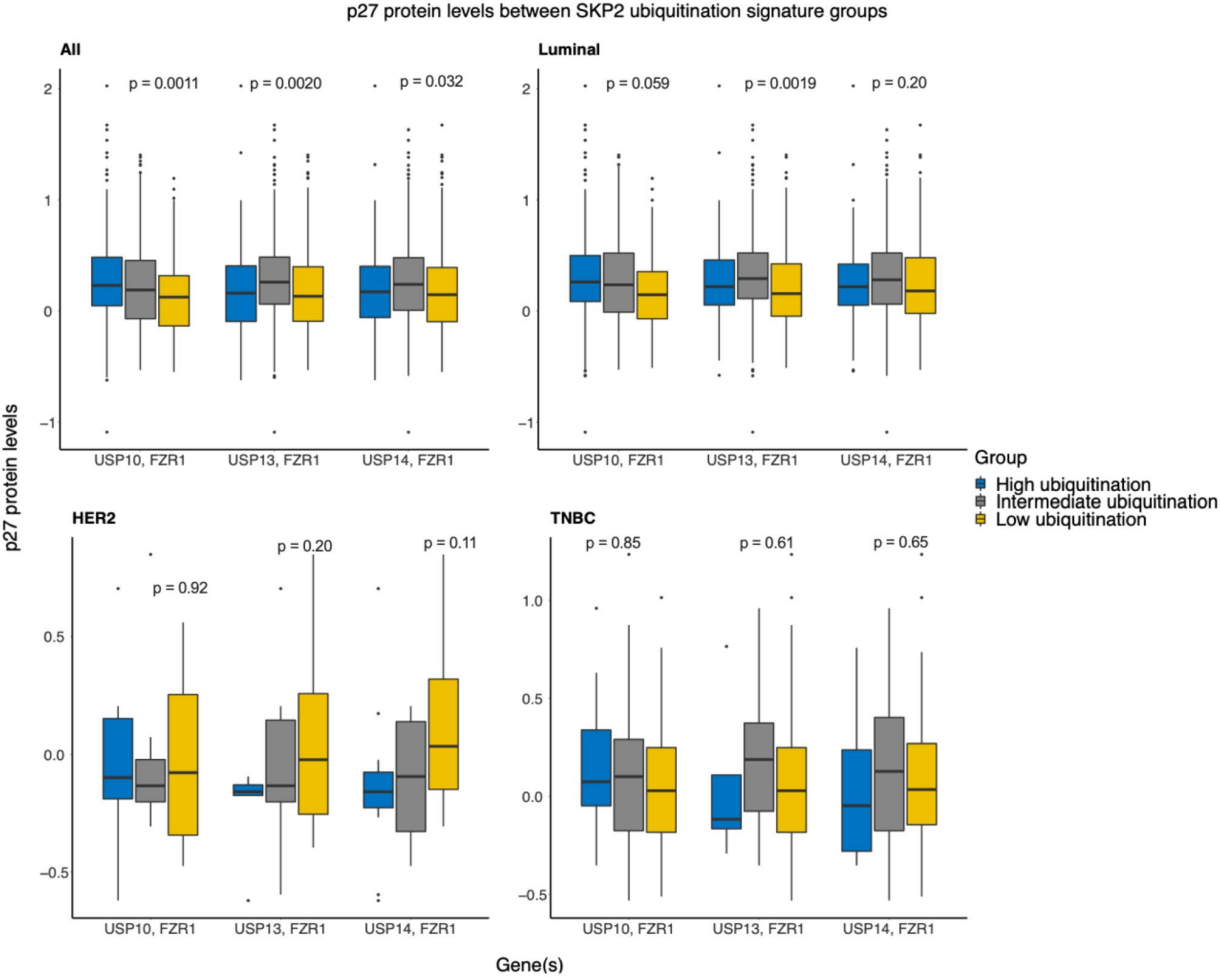
Selection of genes as SKP2 ubiquitination signature. Analyses were performed on the data set in aggregate (*n* = 873) and on three major subtypes separately: luminal (*n* = 629), HER2 (*n* = 36), and triple-negative BC (TNBC) (*n* = 92). Comparison of p27 protein levels between SKP2 ubiquitination groups as defined by our signature (copy number). Luminal BC was defined as being estrogen receptor (ER)-positive (ER^+^); HER2 BC was defined as being HER2-positive (HER2^+^) and ER- and progesterone receptor (PR)-negative (ER^−^, PR^−^); TNBC was defined as being ER^−^, PR^−^, and HER2^−^. Immunohistochemical staining was used to determine the status of each marker. *p*-values shown are for one-way ANOVA.

Next, we examined the SKP2 downstream targets using our gene signature. Both p27 and p21 are known substrates of SKP2 and have been reported to be inversely correlated with SKP2 levels^3,6,31–33^, making them an ideal proxy for the SKP2 level or activity; they are among the most well-documented targets of SKP2 in the literature, an important consideration for validating our signature. However, the association of p21 with breast cancer prognosis is ambiguous and p27 has been more intensively studied^3,6,31–33^. Therefore, we focused on p27 and compared its protein levels between the two groups, assuming that if a gene signature actually reflected levels of SKP2 ubiquitination, the group of patients it classifies as having high SKP2 ubiquitination would have higher levels of p27, and on the other hand the group classified as having low SKP2 ubiquitination would have lower p27. Our reasoning is as follows: higher ubiquitination of SKP2 should lead to increased degradation and lower levels of SKP2; decreased SKP2 should in turn lead to higher levels of p27, since p27 is a target for ubiquitination by SKP2^3,6,31–33^. Of course, we would expect the opposite for lower ubiquitination of SKP2 (Table 1). As illustrated in Fig. 2, a signature consisting of FZR1 and USP10 stratified luminal BC patients into groups of different levels of p27 and in the expected pattern (i.e., the high-ubiquitination group having the highest p27 levels and the low-ubiquitination group having the lowest levels), although this pattern was just above the threshold for significance at a cutoff of *p* = 0.05 (one-way ANOVA *p* = 0.059, *F* = 2.85, group DOF = 2, residuals DOF = 626). The signature consisting of FZR1 and USP13, meanwhile, did not display the expected pattern in any of the subtypes tested, nor did a negative control consisting of FZR1 and USP14, a deubiquitinase whose knockdown was shown not to decrease SKP2 levels (Table S1)^27^.

**Figure 3:**
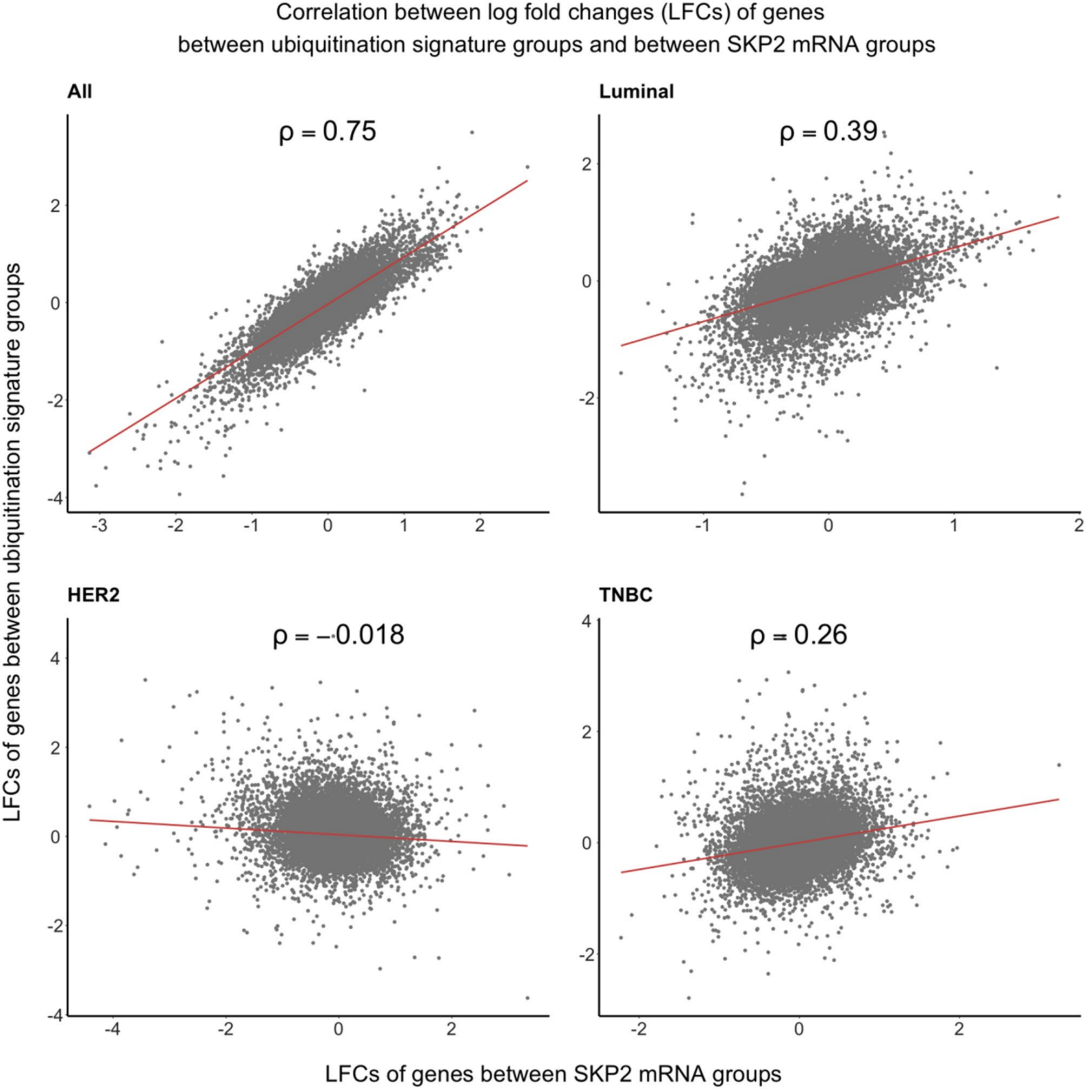
Correlation between Skp2 ubiquitination levels and SKP2 mRNA levels. Correlation between (1) the log fold changes (LFCs) of the genes differentially expressed between the high- and low-SKP2 ubiquitination groups (y-axis) and (2) the LFCs of the genes differentially expressed between high and low SKP2 copy number (x-axis). Analyses were performed on a dataset in aggregate (*n* = 728) and on three major subtypes separately: luminal (*n* = 566), HER2 (*n* = 27), and triple-negative BC (TNBC) (*n* = 62). Number of genes in each dataset = 20,501. Subtypes are defined as in Fig. 2. Spearman’s method was used to calculate the rank correlation. The results of the limma linear-model fits, including all 20,501 genes, can be found in Supplementary File S1.

To further demonstrate that our SKP2 ubiquitination signature can be used to characterize changes in SKP2 protein levels, we determined the extent to which our SKP2-ubiquitination signature can be employed to predict the same downstream effects in terms of alterations in gene expression as SKP2 expression levels change. To this end, we calculated the changes in the expression of all measured genes between two groups of BC patients: those classified by our signature as having high SKP2 ubiquitination and those having low SKP2 ubiquitination. These changes were represented as log fold changes (LFCs). We then did the same for those with high SKP2 expression and those with normal or low SKP2 expression. We expect that if changes in SKP2 levels is truly the mechanism underlying our SKP2 ubiquitination signature, there should be a correlation between the two vectors of LFCs produced above. As seen in Fig. 3, the highest Spearman correlation between the two sets of changes in gene expression occurs in luminal BC, in line with the patterns of differential p27 protein levels as shown in Fig. 2.

### Assessment of the clinical relevance of SKP2 ubiquitination signature

**Figure 4:**
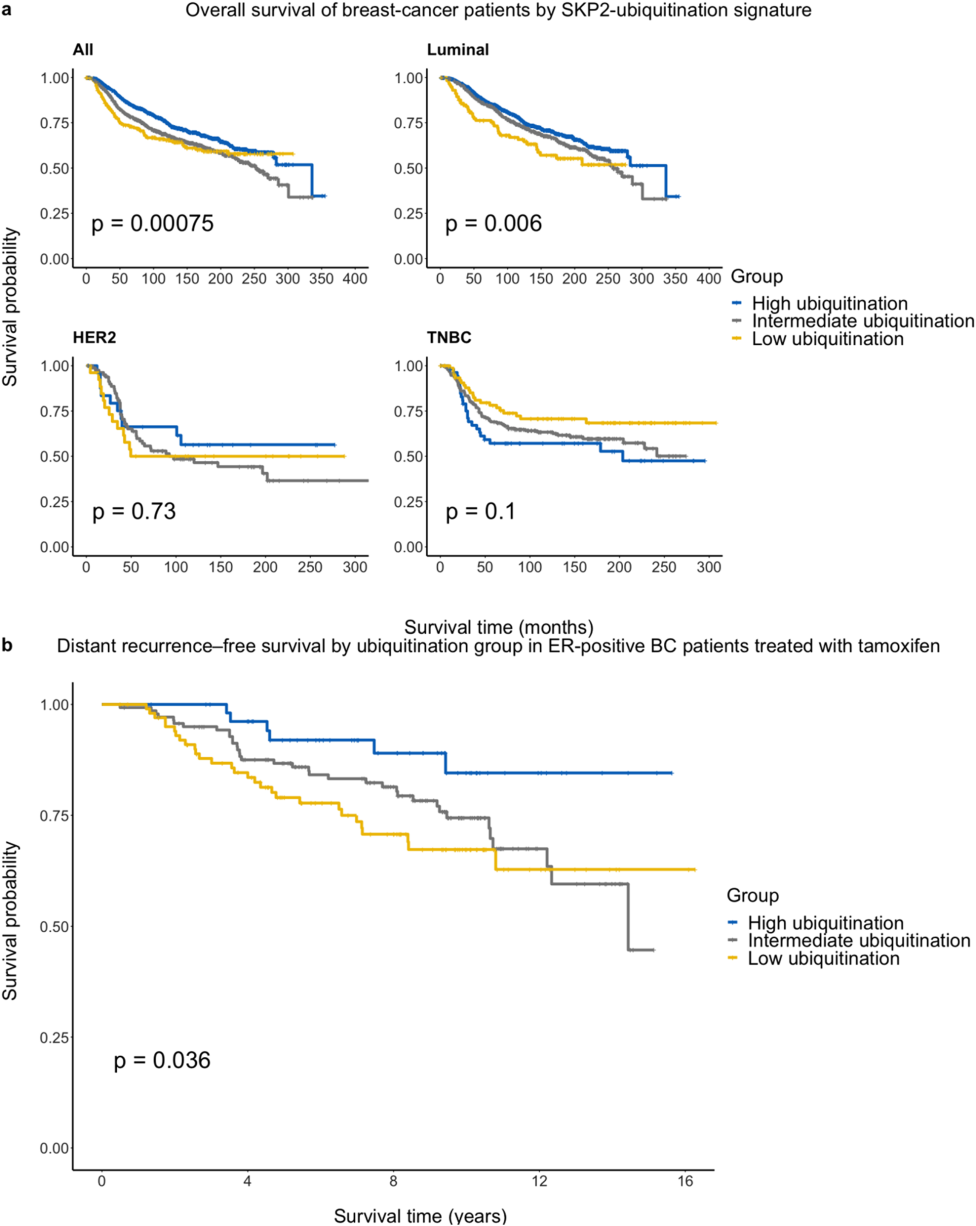
(**a**) Differences in overall survival between the SKP2 ubiquitination groups. Analyses were performed on the data set in aggregate (*n* = 1981) and on three major subtypes separately: luminal (*n* = 1527), HER2 (*n* = 134), and TNBC (*n* = 320). Subtypes are defined as in Fig. 2. The Kaplan–Meier method was used to graph the survival curves, and the log-rank test was used to calculate the significance in difference in survival between ubiquitination groups. (**b**) Distant recurrence-free survival differences between SKP2 ubiquitination-signature groups in ER^+^ BC patients treated with tamoxifen (*n* = 298).

We next sought to determine the ability of our SKP2 ubiquitination signature to correlate with BC patient clinical outcome. First, we determined the extent to which our signature can stratify patients into groups of significantly different survival times. To this end, we used the Molecular Taxonomy of Breast Cancer International Consortium (METABRIC) data set as provided by cBioPortal^34–36^; this data set contains information on both overall survival and copy-number alterations for 1981 of 2173 patients. Using our SKP2 ubiquitination signature, we divided patients into high- and low-SKP2 ubiquitination groups as detailed in the previous section. We graphed the survival curves of the two groups using the Kaplan–Meier method and determined the significance of the survival differences using the log-rank method. Since SKP2 is an oncoprotein, we expected the high-SKP2 ubiquitination group to have better survival. As shown in Fig. 4a (see Table S2 for descriptive statistics), the patterns mirror those seen in comparing the p27 levels between the two groups, with significant survival differences as expected seen in luminal patients (log-rank-test *p* = 0.006) and no significant differences in survival seen in HER2 or TNBC patients (log-rank-test *p* = 0.73 and *p* = 0.1, respectively).

Since the luminal subtype showed the most significant differences in survival between the high- and low-SKP2 ubiquitination groups, we asked next whether our SKP2 ubiquitination signature would be able to distinguish survival outcomes in hormone therapy in luminal-subtype BC patients. To do this, we downloaded the GSE17705 dataset from the NCBI’s Gene Expression Omnibus (GEO) repository consisting of ER^+^ BC patients treated with tamoxifen^37^. Since this data set has gene-expression but not copy number-alteration data, we divided patients into high- and low-SKP2 ubiquitination groups in the following way: patients with FZR1 expression less than or equal to the median FZR1 expression of the data set and USP10 expression greater than the median USP10 expression of the data set were classified as “low SKP2 ubiquitination”. Patients with USP10 expression less than or equal to the median USP10 expression of the data set and FZR1 expression greater than the median FZR1 expression of the data set were classified as “high SKP2 ubiquitination”. All other patients were classified as “intermediate SKP2 ubiquitination”. As shown in Fig. 4b (see Table S3 for descriptive statistics), our signature indeed distinguishes patients by survival outcome (log-rank-test *p* = 0.036).

**Figure 5:**
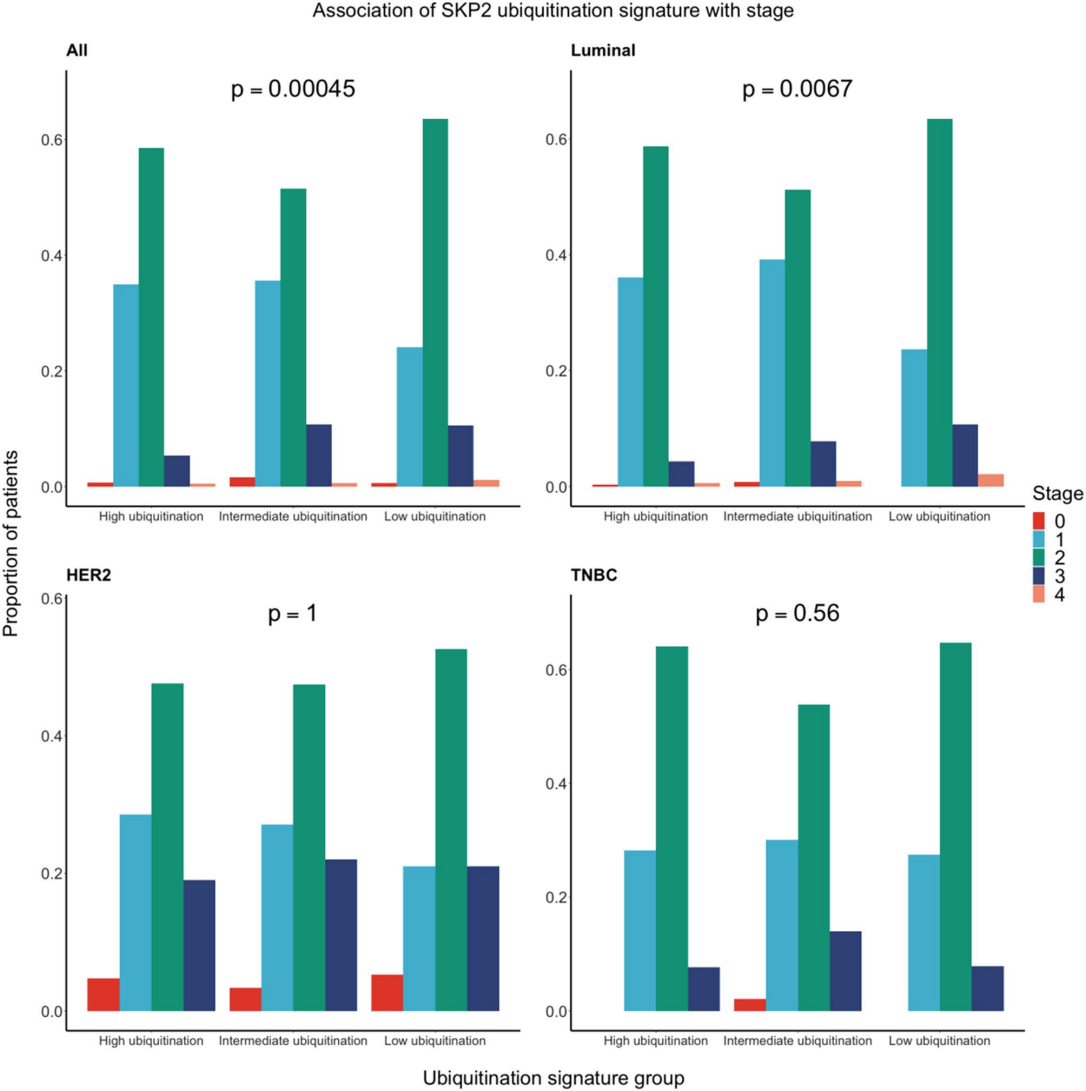
The distribution of patients at a given tumor stage by SKP2 ubiquitination group. Analyses were performed on the data set in aggregate (*n* = 1552) and on three major subtypes separately: luminal (*n* = 1169), HER2 (*n* = 99), and TNBC (*n* = 233). Subtypes are defined as in Fig. 2. The Chi-square test was used to determine the significance of association between ubiquitination group and tumor stage.

**Figure 6:**
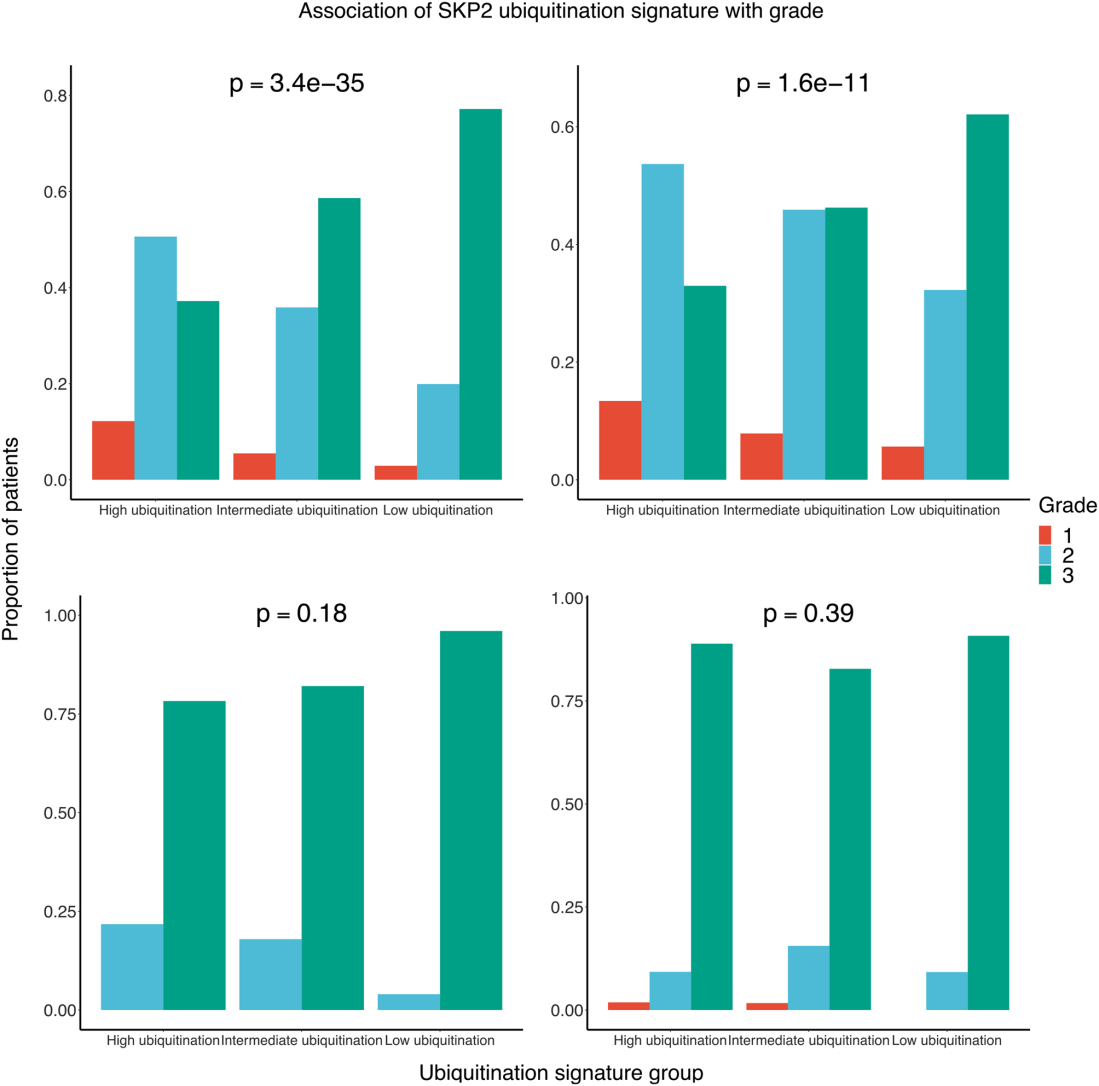
The distribution of patients with a given tumor grade by SKP2 ubiquitination group. Analyses were performed on the data set in aggregate (*n* = 2072) and on three major subtypes separately: luminal (*n* = 1560), HER2 (*n* = 126), and TNBC (*n* = 310). Subtypes are defined as in Fig. 2. The Chi-square test was used to determine the significance of association between ubiquitination group and tumor grade.

Taking one step deeper, we examined how our signature is associated with stage, tumor grade, and number of axillary lymph nodes positive for cancer-cell invasion (i.e., positive lymph nodes). For stage and tumor grade, we hypothesized that higher stage and grade would be associated with lower levels of SKP2 ubiquitination as classified by our signature. For the number of positive lymph nodes, we expected that patients classified as having lower levels of SKP2 ubiquitination would have more positive lymph nodes than patients classified as having higher levels of SKP2 ubiquitination. For these analyses, we used TCGA BC data as accessed through the cBioPortal website. Chi-squared tests were used to determine the association between stage/grade and our SKP2-ubiquitination signature, and negative binomial regression was used to determine the association between number of positive nodes and our SKP2-ubiquitination signature. As seen in Figs. 5 and 6, the proportion of patients with a given stage/grade differs significantly among signature groups in luminal BC (Chi-square *p* = 0.0067 and *p* = 1.6 × 10^−11^, respectively) but not in HER2 BC (Chi-square *p* = 1 and *p* = 0.18, respectively) or TNBC (Chi-square *p* = 0.56 and *p* = 0.39, respectively). The relationship between our SKP2-ubiquitination signature and stage/grade in luminal BC follows the expected pattern, with the low-ubiquitination group having higher proportions of patients with higher-grade or higher-stage tumors.

**Figure 7:**
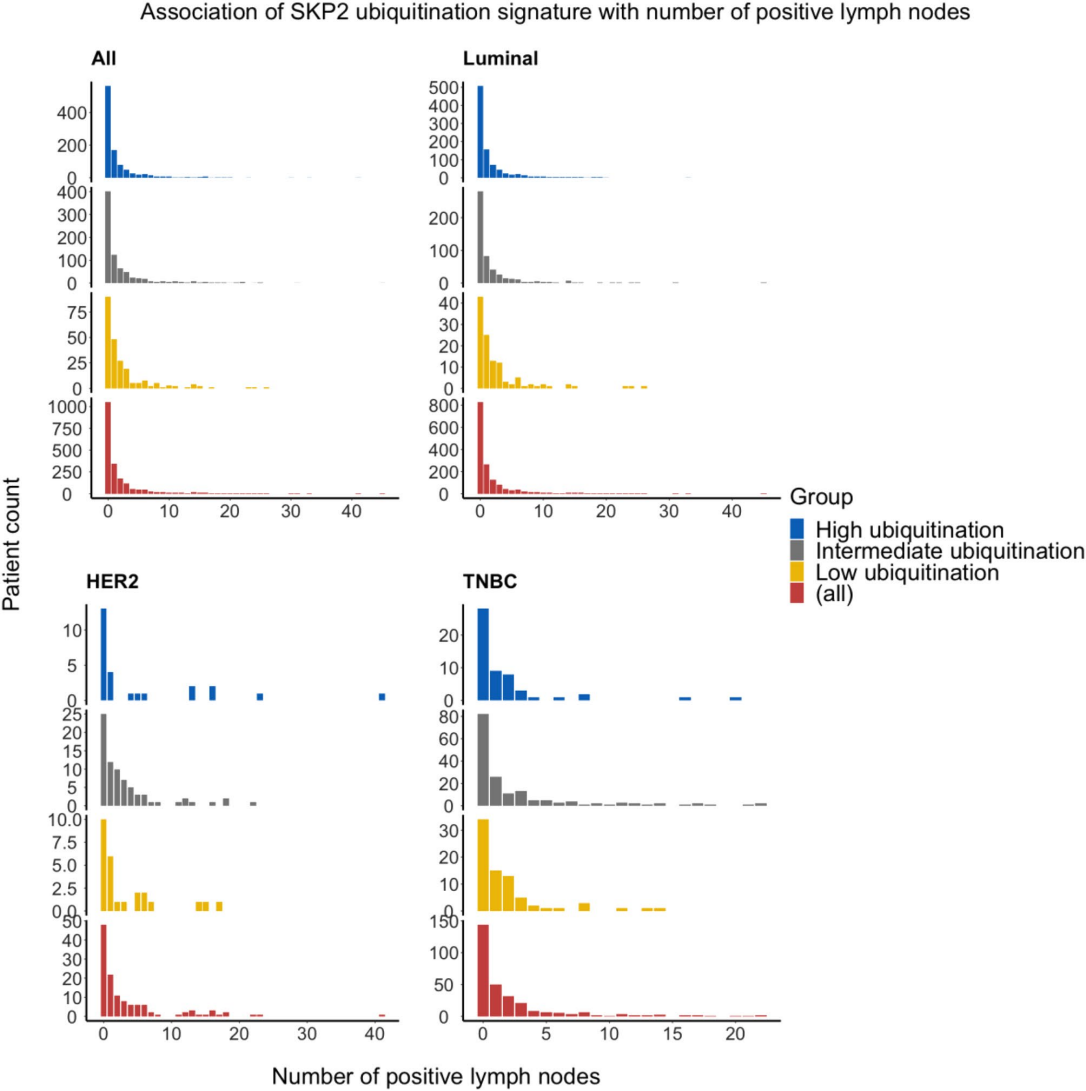
Comparison of the number of positive lymph nodes per patient among the SKP2 ubiquitination groups. Analyses were performed on the data set in aggregate (*n* = 2004) and on three major subtypes separately: luminal (*n* = 1542), HER2 (*n* = 127), and TNBC (*n* = 299). Subtypes are defined as in Fig. 2. Negative binomial regression was used to model count data. The means and variances of the counts of positive lymph nodes for each BC subtype is as follows: All: *μ* = 2.00; *σ*^2^ = 16.84. Luminal: *μ* = 1.80; *σ*^2^ = 14.52. HER2: *μ* = 3.70; *σ*^2^ = 37.77. TNBC: *μ* = 2.27; *σ*^2^ = 16.95.

The relationship between our SKP2-ubiquitination signature and number of positive lymph nodes again followed the expected pattern in luminal BC but not in HER2 BC or TNBC. Because the counts of positive lymph nodes follow a positively skewed distribution but the variance is unequal to the mean (Fig. 7), we fit a negative binomial regression model to the counts. The input variable is the SKP2-ubiquitination signature, and the output variable is the count of patients with a certain number of positive lymph nodes. As exhibited in Table 2, the coefficients for the input variable when the SKP2-ubiquitination signature is intermediate- or low-ubiquitination are positive and significant (Wald *p* = 5.2 × 10^−11^ and *p* = 6.5 × 10^−3^, respectively). This indicates that the count of patients with higher numbers of positive lymph nodes is expected to be higher in patients with an intermediate- or low-ubiquitination signature than in patients with a high-ubiquitination signature, as we expected.

**Table 2:** Coefficients for the negative binomial regression model fit to the count data. Positive coefficients indicate a higher expected count value for that factor level (i.e., ubiquitination signature group) compared to the baseline factor level (here, high ubiquitination). Significance levels are indicated by asterisks as follows: ***p < 0.001, **0.001 < = *p* < 0.01, *0.01 < = *p* < 0.05, no text: 0.05 < = *p* < = 1.

Factor level	Estimate	Std. error	z-value	p-value	Significance
**All**					
High ubiquitination (Intercept)	0.51	0.061	8.4	6.3e−17	***
Intermediate ubiquitination	0.32	0.091	3.5	5.4e−04	***
Low ubiquitination	0.39	0.14	2.8	5.3e−03	**
**Luminal**					
High ubiquitination (Intercept)	0.43	0.065	6.6	5.2e−11	***
Intermediate ubiquitination	0.29	0.11	2.7	6.5e−03	**
Low ubiquitination	0.58	0.19	3.1	2.0e−03	**
**HER2**					
High ubiquitination (Intercept)	1.70	0.31	5.4	7.0e−08	***
Intermediate ubiquitination	− 0.52	0.37	− 1.4	1.6e−01	
Low ubiquitination	− 0.49	0.45	− 1.1	2.7e−01	
**TNBC**					
High ubiquitination (Intercept)	0.58	0.25	2.3000	0.02	*
Intermediate ubiquitination	0.40	0.28	1.4000	0.16	
Low ubiquitination	0.00081	0.32	0.0025	1.00	

### Using SKP2 ubiquitination signature to discover potential treatments for luminal breast cancer

Since our SKP2 ubiquitination signature was shown to be significantly associated with differences in clinical outcome in luminal BC, we wanted to determine if our signature (or more specifically, its downstream effects) could be used to identify treatments for luminal BC from FDA-approved drugs. To do this, we again used the SKP2 ubiquitination signature to stratify patients into two groups of high- and low-SKP2 ubiquitination, then used the limma algorithm to identify the genes that were differentially expressed between the two groups^38^. We input this list of genes to the Connectivity Map’s (CMap)^39^ repurposing function via the DrInsight package and identified drugs that produce either a similar or an opposite perturbational gene signature (Fig. S1)^40^. Table 3 shows the drugs predicted by CMap to yield either the desired transcriptional perturbation (negative) or the opposite perturbation (positive). Of the seven of these drugs predicted to have the desired transcriptional perturbation in a luminal BC cell line (MCF7), two have been shown to downregulate SKP2 and stabilize p27 in BC cell lines (sirolimus and vorinostat), and one has been shown to induce G1 arrest in BC cell lines independent of DRD2 (thioridazine)^41–43^. Interestingly, one drug predicted to produce a positive perturbation profile (i.e., lead to a transcriptional profile more similar to that of the low-ubiquitination group) is estradiol, the major estrogen hormone. As covered in more detail in the “Discussion” section, several studies suggest that SKP2 plays an essential role in estrogen signaling, which drives malignancies such as luminal BC and endometrial cancer^44–46^.

**Table 3:** List of drugs identified as reversing the perturbational gene signature (negative perturbation profiles and therefore recommended) or promoting the perturbational gene signature (positive perturbation profiles and therefore not recommended) between the high- and low-SKP2 ubiquitination signature groups based on the differential gene-expression data in Supplementary File S1. Only drugs with a false-discovery rate < 0.05 were considered. BC cell lines are marked in bold.

Drug	Cell line	p-value	False-discovery rate
**Drugs with negative perturbation profiles (recommended drugs)**			
Trichostatin A	**MCF7**	3.5e−45	1.3e−41
Trichostatin A	PC3	8.4e−24	1.5e−20
Sirolimus	**MCF7**	1.0e−15	1.2e−12
LY-294002	**MCF7**	4.7e−13	4.2e−10
Trichostatin A	HL60	1.3e−12	9.0e−10
Fulvestrant	**MCF7**	1.4e−09	8.5e−07
Wortmannin	**MCF7**	6.6e−09	3.4e−06
Tanespimycin	HL60	2.1e−08	9.5e−06
Sirolimus	PC3	2.2e−07	8.9e−05
LY-294002	PC3	4.0e−07	1.4e−04
Tanespimycin	**MCF7**	8.8e−06	2.9e−03
Vorinostat	**MCF7**	1.2e−05	3.7e−03
LY-294002	HL60	1.5e−05	4.2e−03
Prochlorperazine	**MCF7**	4.7e−05	1.2e−02
Thioridazine	**MCF7**	1.1e−04	2.6e−02
**Drugs with positive perturbation profiles**			
Estradiol	MCF7	3.1e−06	0.011

**Figure 8:**
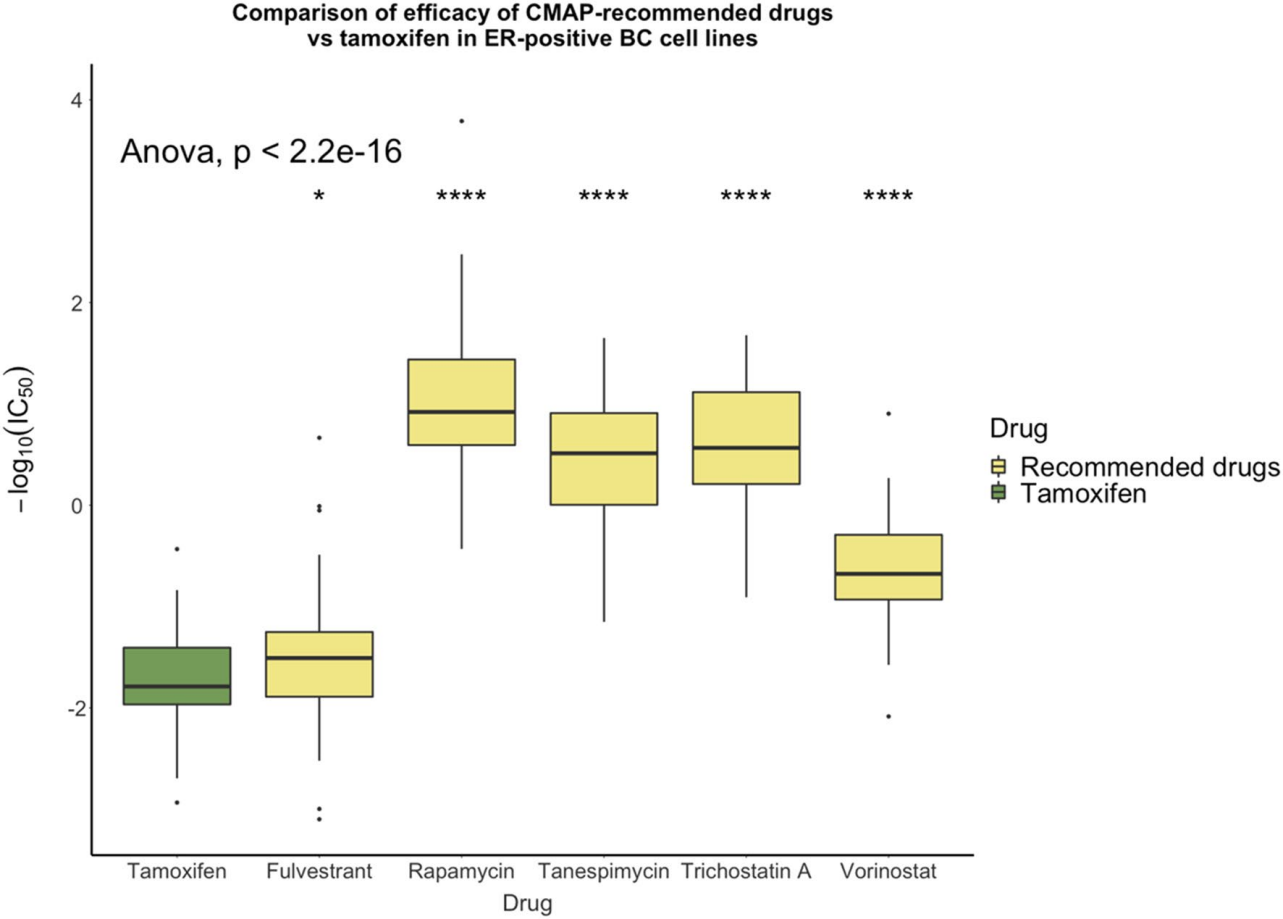
Comparison in ER^+^ BC cell lines of the IC_50_ values of the drugs recommended by CMap vs tamoxifen. ANOVA was used to detect unequal means within the groups, and Student’s *t-*test was used to determine the significance of between-group differences in IC_50_ values. Significance levels are indicated by asterisks as follows: ***p < 0.001, **0.001 < = *p* < 0.01, *0.01 < = *p* < 0.05, no text: 0.05 < = *p* < = 1.

To further assess the potential of the CMap-identified drugs as repurposed therapeutics for ER^+^ BC, we compared the IC_50_s of these drugs against that of the standard-of-care tamoxifen using data from the Genomics of Drug Sensitivity in Cancer (GDSC) database^47–49^. We identified ER^+^ BC cell lines per Stemke-Hale et al.^50^ in GDSC and used ANOVA to compare the IC_50_s of tamoxifen for these cell lines with those of the five CMap-identified drugs for which data were available in GDSC (fulvestrant, rapamycin, tanespimycin, trichostatin A, and vorinostat). As seen in Fig. 8, the mean IC_50_ of each of these drugs is significantly lower than that of tamoxifen, further suggesting that these drugs could be repurposed to treat ER^+^ BC.

## Discussion

In this study, we have developed a gene signature consisting of two genes, USP10 and FZR1, known to control the ubiquitination of the oncoprotein SKP2. FZR1, as part of the APC/C ligase complex, ubiquitinates SKP2 protein and marks it for degradation, while USP10, a deubiquitinase, removes ubiquitin from SKP2. This gene signature is presumably highly related to the levels of SKP2 as well as its downstream tumor suppressor targets such as p27. We therefore expected our signature to be able to stratify patients into groups of high, low, and intermediate SKP2 ubiquitination. The high-ubiquitination group is defined as having higher copy numbers of FZR1 than USP10 (i.e., higher copy numbers of the ubiquitinase component than the deubiquitinase), the low-ubiquitination group as the opposite, and the intermediate-ubiquitination group is defined as having similar copy numbers of the two genes. By measuring the levels of p27, a protein whose levels are controlled by SKP2, we provided indirect evidence that our signature is indeed able to stratify patients into groups of high and low SKP2 protein. Although p21 is also a target of SKP2 ubiquitination, we selected p27 for this analysis since the association of p21 levels in breast cancer remains ambiguous, possibly due to the involvement of p21 in other signaling pathways, while higher levels of p27 are more clearly associated with better prognosis^51–56^. Indeed, p21 levels did not show the same patterns as p27 across all ubiquitin-signature groups (Fig. S2 and Table S4, cf. Fig. 2 and Table S1).

We anticipate that our signature would also have clinical applicability, as SKP2 protein levels are negatively correlated with expression of the tumor suppressor p27 and with survival, and positively correlated with proliferation. Our study demonstrates this gene signature is indeed associated with survival, stage, grade, and the average number of positive lymph nodes in BC, but it is interesting to note that these associations were only present in the luminal subtype of BC; the fact that the luminal subtype is by far the most prevalent in the METABRIC dataset, accounting for over 75% of the samples (1637/2173), may explain why the patterns seen in the cohort as a whole mirror those in the luminal subtype. One possible explanation for this observation is that in breast cancer, SKP2-induced G1/S transition and progression through S phase is at least in part mediated by estrogen receptor alpha (ERα)^44^. In particular, the ubiquitination of ERα by SKP2 is important for ERα’s *trans*-activation of its downstream target genes^44,46^. Moreover, it is interesting to note that in endometrial cancer, a malignancy largely driven by estrogen signaling (as is luminal BC), estrogen decreases APC/C^FZR1^ levels, causing a subsequent increase in SKP2-mediated p27 degradation; and SKP2 knockdown or inhibition prevents estrogen-driven p27 degradation and cell growth^45,57^. Taken together, these observations suggest that SKP2’s oncogenic activity is mediated through ERα and plays a vital role in estrogen signaling in hormone-driven (i.e. luminal) BC and may explain why our signature, based on the control of SKP2 protein levels, is less correlated with clinical outcome in the ER^−^ subtypes. However, 50–70% of BCs are luminal^58^, meaning that there is a large patient population in which our gene signature may be used for prognosis and treatment.

Although we have shown that our signature is associated with clinical outcome, several further questions may arise concerning the rationale for our signature’s use in the clinic. As mentioned earlier, we used copy number rather than mRNA levels or mutations based on the following considerations: first, copy number is more stable than mRNA levels as transcription can be changed in response to external stimuli. Second, copy number was shown in a pan-cancer study to be more prognostic than mutations in cancer driver genes^29^. To confirm the relation between copy number and expression in our genes of interest, we examined the expression for SKP2, USP10, USP13, and FZR1 in the METABRIC data set and found that expression indeed increases with copy number (Fig. S3, Table S5). Our signature takes into account two genes: a ubiquitin ligase component (FZR1) and a deubiquitinase (USP10.) While this restriction undoubtedly prevents the signature from comprehensively accounting for the SKP2 axis, the simplicity makes it amenable for use by clinicians, allowing for easy stratification of patients into three groups distinguished by biological characteristics readily apparent from the signature (Table 1). As our results show, the signature does appear to have clinical relevancy. Finally, we examined whether our signature has any advantages over the use of SKP2 copy number directly or the use of USP10, USP13, or FZR1 copy numbers individually. While SKP2 protein levels have been demonstrated to have prognostic power in BC, to date there are no published studies showing that SKP2 copy number is similarly useful as a prognostic biomarker. Indeed, our own analyses show that neither copy number nor expression of either SKP2, USP10, USP13, or FZR1 individually is as well correlated with clinical outcome as our ubiquitination signature (Figs. S4–S12, Tables S6–S10).

This study also provides a rationale to further pursue several lines of inquiry. First, as demonstrated in our results, several FDA-approved drugs show potential as repurposed therapies for ER^+^ BC. Several of the drugs identified here are known to have anti-cancer effects, and one, fulvestrant, is already used to treat hormone receptor-positive, HER2-negative metastatic BC in post-menopausal women^59^, bolstering our confidence in the other recommended drugs. It is also interesting to note that the ranking of tamoxifen in the CMap study compared to the recommended drugs agrees with its comparative ranking in terms of IC_50_, in that it is ranked lower in both cases (tamoxifen not shown in Table 3 because its false-discovery rate exceeded the cutoff). This suggests that the use of gene-perturbation signatures is an effective way to identify potential anti-cancer therapeutics. CMap is not limited to breast cancer, therefore it may be worth examining how therapeutics identified based on a ubiquitination gene signature work for other cancer types (e.g., tanespimycin for HL60 of leukemia, sirolimus for PC3 of prostate cancer). Next, as mentioned before, USP10 has not yet been shown either in vitro or in vivo to deubiquitinate SKP2 in BC specifically; so far, it has only been shown to deubiquitinate SKP2 in chronic myelogenous leukemia^27^. Our results therefore provide an impetus to further investigate the role of USP10 in BC, especially as a possible therapeutic target. This study also raises the possibility of using a bioinformatic approach as we have taken here to identify potential deubiquitinases of other proteins in cancers. It is highly worth noting that, although this study focuses on the ubiquitination of a single protein (SKP2) in a single cancer type, it has implications for other proteins in other cancer types, since ubiquitination and proteasomal degradation is a universal cellular process. Our investigation here lays the groundwork for potential future studies of using ubiquitinase and deubiquitinase genes as signatures to stratify patients and predict clinical outcome.

## Methods

### Programming environment

All analyses were conducted in R 3.6.3 running on RStudio 1.3.959.

### Data curation and processing

TCGA and METABRIC data were obtained from cBioPortal using the R packages CGDSR and TCGA2STAT^34–36,60^. The copy-number data as provided by cBioPortal assigns a copy-number level to each gene based on the copy-number data in the original studies. The cBioPortal copy-number levels are as follows: − 2 indicates a deep loss; − 1 indicates a shallow loss; 0 indicates diploidy; 1 indicates a low-level gain; and 2 indicates a high-level gain. Comparison of copy number in our analyses was performed using these levels. The copy-number data for TCGA was generated using the GISTIC algorithm, and the copy-number data for METABRIC was generated using the GISTIC2 algorithm, as described previously^61,62^.

Another data set from the NCBI Gene Expression Omnibus (GEO) website, GSE17705, was used in analysis of clinical outcome^37^, and it contains ER^+^ patients treated with adjuvant tamoxifen (*n* = 298). Samples were collected by surgical excision and frozen; no information on whether patients were treated with neoadjuvant therapy was available. GSE17705 was downloaded from the GEO website as series matrix files and raw microarray CEL files (Affymetrix Human Genome U133A Array). The raw CEL files were read into R using the package simpleaffy and normalized using the GCRMA method as implemented in the gcrma package. Probesets for the genes studied in the analysis of GSE17705 were selected using the Jetset method^63^.

GDSC data were obtained from https://www.cancerrxgene.org/^47–49^.

Where appropriate, all data in the original data sets were obtained in accordance with the relevant guidelines and regulations and with approval of the respective institutional review boards.

### Statistical analysis

Associations between categorical variables (e.g., gene-signature group and clinical outcome group) were assessed using the Chi-square test. Correlations between continuous variables were calculated using Spearman’s rank correlation coefficient. Survival analysis was conducted using the log-rank test, and results were graphed using the Kaplan–Meier method. Association between groups and continuous variables were assessed using ANOVA. Comparisons of continuous variables between two groups were performed using Student’s *t*-test. Negative binomial regression was used to model count data. The significance of the coefficients resulting from negative binomial regression was calculated using the Wald test, as implemented in the MASS R package^64^. The limma algorithm was used to perform differential expression analysis^38^. Two-tailed tests were used where applicable, and results were considered significant at *p* < 0.05 unless otherwise stated.

## Supplementary Information

Supplementary Table S1.

Supplementary Information.

## Data Availability

All scripts used in this analysis are available upon request.

## References

[1] Gstaiger M (2001). Skp2 is oncogenic and overexpressed in human cancers. Proc. Natl. Acad. Sci..

[2] Wang Z (2012). Skp2 is a promising therapeutic target in breast cancer. Front. Oncol..

[3] Carrano AC, Eytan E, Hershko A, Pagano M (1999). SKP2 is required for ubiquitin-mediated degradation of the CDK inhibitor p27. Nat. Cell Biol..

[4] Tsvetkov LM, Yeh KH, Lee SJ, Sun H, Zhang H (1999). p27(Kip1) ubiquitination and degradation is regulated by the SCF(Skp2) complex through phosphorylated Thr187 in p27. Curr. Biol. CB.

[5] Yu ZK, Gervais JL, Zhang H (1998). Human CUL-1 associates with the SKP1/SKP2 complex and regulates p21(CIP1/WAF1) and cyclin D proteins. Proc. Natl. Acad. Sci. U.S.A..

[6] Bornstein G (2003). Role of the SCFSkp2 ubiquitin ligase in the degradation of p21Cip1 in S phase. J. Biol. Chem..

[7] Kossatz U (2004). Skp2-dependent degradation of p27kip1 is essential for cell cycle progression. Genes Dev..

[8] Xiong Y (1993). p21 is a universal inhibitor of cyclin kinases. Nature.

[9] Harper JW, Adami GR, Wei N, Keyomarsi K, Elledge SJ (1993). The p21 Cdk-interacting protein Cip1 is a potent inhibitor of G1 cyclin-dependent kinases. Cell.

[10] Shin I (2002). PKB/Akt mediates cell-cycle progression by phosphorylation of p27Kip1 at threonine 157 and modulation of its cellular localization. Nat. Med..

[11] Liang J (2002). PKB/Akt phosphorylates p27, impairs nuclear import of p27 and opposes p27-mediated G1 arrest. Nat. Med..

[12] Abbas T, Dutta A (2009). p21 in cancer: Intricate networks and multiple activities. Nat. Rev. Cancer.

[13] Carrano AC, Pagano M (2001). Role of the F-box protein Skp2 in adhesion-dependent cell cycle progression. J. Cell Biol..

[14] Chan C-H, Lee S-W, Wang J, Lin H-K (2010). Regulation of Skp2 expression and activity and its role in cancer progression. ScientificWorldJournal.

[15] Asmamaw MD, Liu Y, Zheng Y-C, Shi X-J, Liu H-M (2020). Skp2 in the ubiquitin-proteasome system: A comprehensive review. Med. Res. Rev..

[16] Cai Z (2020). The Skp2 pathway: A critical target for cancer therapy. Semin. Cancer Biol..

[17] Wu L (2012). Specific small molecule inhibitors of Skp2-mediated p27 degradation. Chem. Biol..

[18] Chan C-H (2013). Pharmacological inactivation of Skp2 SCF ubiquitin ligase restricts cancer stem cell traits and cancer progression. Cell.

[19] Zhao H (2020). Targeted inhibition of the E3 ligase SCFSkp2/Cks1 has antitumor activity in RB1-deficient human and mouse small-cell lung cancer. Cancer Res..

[20] Porter PL (1997). Expression of cell-cycle regulators p27Kip1 and cyclin E, alone and in combination, correlate with survival in young breast cancer patients. Nat. Med..

[21] Catzavelos C (1997). Decreased levels of the cell-cycle inhibitor p27Kip1 protein: Prognostic implications in primary breast cancer. Nat. Med..

[22] Slotky M (2005). The expression of the ubiquitin ligase subunit Cks1 in human breast cancer. Breast Cancer Res. BCR.

[23] Sonoda H (2006). Significance of skp2 expression in primary breast cancer. Clin. Cancer Res. Off. J. Am. Assoc. Cancer Res..

[24] Bashir T, Dorrello NV, Amador V, Guardavaccaro D, Pagano M (2004). Control of the SCF (Skp2-Cks1) ubiquitin ligase by the APC/C(Cdh1) ubiquitin ligase. Nature.

[25] Wei W (2004). Degradation of the SCF component Skp2 in cell-cycle phase G1 by the anaphase-promoting complex. Nature.

[26] Chen M, Gutierrez GJ, Ronai ZA (2011). Ubiquitin-recognition protein Ufd1 couples the endoplasmic reticulum (ER) stress response to cell cycle control. Proc. Natl. Acad. Sci. U.S.A..

[27] Liao Y (2019). USP10 modulates the SKP2/Bcr-Abl axis via stabilizing SKP2 in chronic myeloid leukemia. Cell Discov..

[28] Xia X (2021). USP10 exacerbates neointima formation by stabilizing Skp2 protein in vascular smooth muscle cells. J. Biol. Chem..

[29] Smith JC, Sheltzer JM (2018). Systematic identification of mutations and copy number alterations associated with cancer patient prognosis. eLife.

[30] Fujita T, Liu W, Doihara H, Wan Y (2009). An in vivo study of Cdh1/APC in breast cancer formation. Int. J. Cancer.

[31] Nakayama K (2004). Skp2-mediated degradation of p27 regulates progression into mitosis. Dev. Cell.

[32] Wang W, Nacusi L, Sheaff RJ, Liu X (2005). Ubiquitination of p21Cip1/WAF1 by SCFSkp2: Substrate requirement and ubiquitination site selection. Biochemistry.

[33] Davidovich S, Ben-Izhak O, Shapira M, Futerman B, Hershko DD (2008). Over-expression of Skp2 is associated with resistance to preoperative doxorubicin-based chemotherapy in primary breast cancer. Breast Cancer Res..

[34] Mukherjee A (2018). Associations between genomic stratification of breast cancer and centrally reviewed tumour pathology in the METABRIC cohort. Npj Breast Cancer.

[35] Cerami E (2012). The cBio cancer genomics portal: An open platform for exploring multidimensional cancer genomics data. Cancer Discov..

[36] Gao J (2013). Integrative analysis of complex cancer genomics and clinical profiles using the cBioPortal. Sci. Signal..

[37] Symmans WF (2010). Genomic index of sensitivity to endocrine therapy for breast cancer. J. Clin. Oncol. Off. J. Am. Soc. Clin. Oncol..

[38] Ritchie ME (2015). Limma powers differential expression analyses for RNA-sequencing and microarray studies. Nucleic Acids Res..

[39] Lamb J (2006). The Connectivity Map: Using gene-expression signatures to connect small molecules, genes, and disease. Science.

[40] Chan J, Wang X, Turner JA, Baldwin NE, Gu J (2019). Breaking the paradigm: Dr Insight empowers signature-free, enhanced drug repurposing. Bioinformatics.

[41] Shapira M, Kakiashvili E, Rosenberg T, Hershko DD (2006). The mTOR inhibitor rapamycin down-regulates the expression of the ubiquitin ligase subunit Skp2 in breast cancer cells. Breast Cancer Res. BCR.

[42] Tegowski M, Fan C, Baldwin AS (2018). Thioridazine inhibits self-renewal in breast cancer cells via DRD2-dependent STAT3 inhibition, but induces a G1 arrest independent of DRD2. J. Biol. Chem..

[43] Uehara N, Yoshizawa K, Tsubura A (2012). Vorinostat enhances protein stability of p27 and p21 through negative regulation of Skp2 and Cks1 in human breast cancer cells. Oncol. Rep..

[44] Bhatt S, Xiao Z, Meng Z, Katzenellenbogen BS (2012). Phosphorylation by p38 mitogen-activated protein kinase promotes estrogen receptor α turnover and functional activity via the SCFSkp2 proteasomal complex. Mol. Cell. Biol..

[45] Pavlides SC (2013). Inhibitors of SCF-Skp2/Cks1 E3 ligase block estrogen-induced growth stimulation and degradation of nuclear p27kip1: Therapeutic potential for endometrial cancer. Endocrinology.

[46] Zhou W, Srinivasan S, Nawaz Z, Slingerland JM (2014). ERα, SKP2 and E2F-1 form a feed forward loop driving late ERα targets and G1 cell cycle progression. Oncogene.

[47] Garnett MJ (2012). Systematic identification of genomic markers of drug sensitivity in cancer cells. Nature.

[48] Yang W (2013). Genomics of Drug Sensitivity in Cancer (GDSC): A resource for therapeutic biomarker discovery in cancer cells. Nucleic Acids Res..

[49] Iorio F (2016). A landscape of pharmacogenomic interactions in cancer. Cell.

[50] Stemke-Hale K (2008). An integrative genomic and proteomic analysis of PIK3CA, PTEN, and AKT mutations in breast cancer. Cancer Res..

[51] Elledge RM, Allred DC (1998). Prognostic and predictive value of p53 and p21 in breast cancer. Breast Cancer Res. Treat..

[52] Weiss RH (2003). p21Waf1/Cip1 as a therapeutic target in breast and other cancers. Cancer Cell.

[53] Alkarain A, Jordan R, Slingerland J (2004). p27 deregulation in breast cancer: Prognostic significance and implications for therapy. J. Mammary Gland Biol. Neoplasia.

[54] Sgambato A (1997). Deregulated expression of p27 (Kip1) in human breast cancers. Clin. Cancer Res. Off. J. Am. Assoc. Cancer Res..

[55] Chiarle R, Pagano M, Inghirami G (2000). The cyclin dependent kinase inhibitor p27 and its prognostic role in breast cancer. Breast Cancer Res..

[56] Viglietto G (2002). Cytoplasmic relocalization and inhibition of the cyclin-dependent kinase inhibitor p27Kip1 by PKB/Akt-mediated phosphorylation in breast cancer. Nat. Med..

[57] Huang K-T (2012). Estrogen and progesterone regulate p27kip1 levels via the ubiquitin-proteasome system: Pathogenic and therapeutic implications for endometrial cancer. PLoS One.

[58] Fragomeni SM, Sciallis A, Jeruss JS (2018). Molecular subtypes and local-regional control of breast cancer. Surg. Oncol. Clin. N. Am..

[59] Blackburn SA, Parks RM, Cheung K-L (2018). Fulvestrant for the treatment of advanced breast cancer. Expert Rev. Anticancer Ther..

[60] Wan Y-W, Allen GI, Liu Z (2016). TCGA2STAT: Simple TCGA data access for integrated statistical analysis in R. Bioinformatics.

[61] The Cancer Genome Atlas Network (2012). Comprehensive molecular portraits of human breast tumors. Nature.

[62] Pereira B (2016). The somatic mutation profiles of 2,433 breast cancers refines their genomic and transcriptomic landscapes. Nat. Commun..

[63] Li Q, Birkbak NJ, Gyorffy B, Szallasi Z, Eklund AC (2011). Jetset: Selecting the optimal microarray probe set to represent a gene. BMC Bioinform..

[64] Venables WN, Ripley BD, Venables WN, Ripley BD (2002). Generalized linear models. Modern Applied Statistics with S.

