# Supplementary material for: A gene signature consisting of ubiquitin ligases and deubiquitinating enzymes of SKP2 is associated with clinical outcome in breast cancer
Source: Sci Rep. 2022 Feb 15;12:2478. doi: 10.1038/s41598-022-06451-w (PMC8847659; doi:10.1038/s41598-022-06451-w)
Supplement: Supplementary file 2 — Supplementary Information. [file 41598_2022_6451_MOESM2_ESM.pdf]

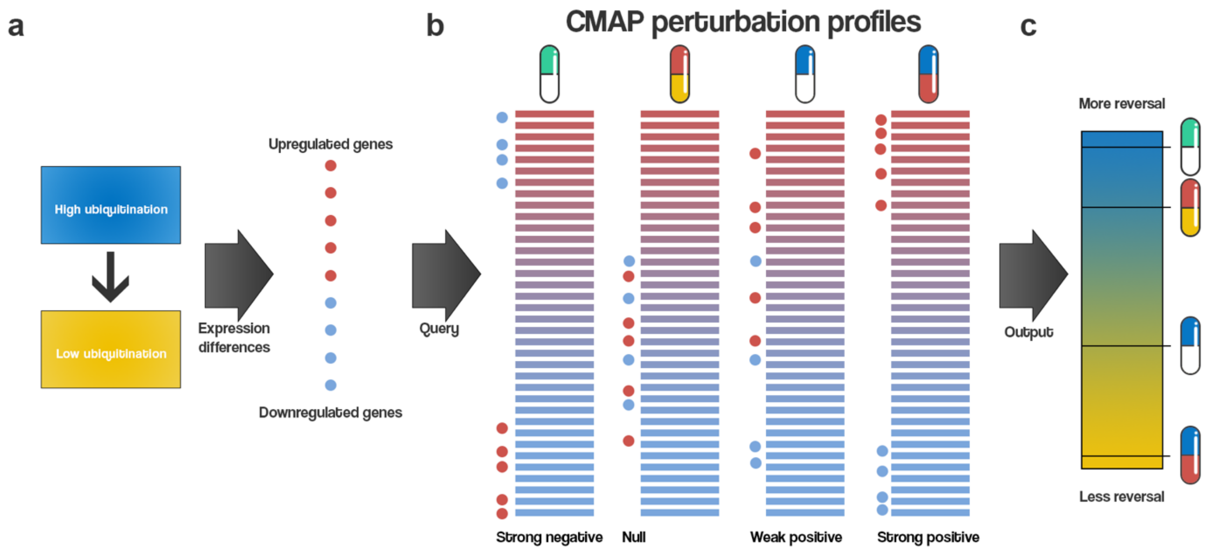

Fig. S1. A scheme used to identify potential drugs for the treatment of ER$^+$ BC based on the approach (Supplementary Reference 1). **a.** Calculation of the differences in gene expression between the high-ubiquitination and low-ubiquitination groups. **b.** Query of CMAP using the differential expression profile generated in **a**. **c.** Output from the CMAP query. Drugs that reverse the input transcriptional profile and therefore would be expected to produce a transcriptional profile closer to that of the high-ubiquitination group are ranked more highly.

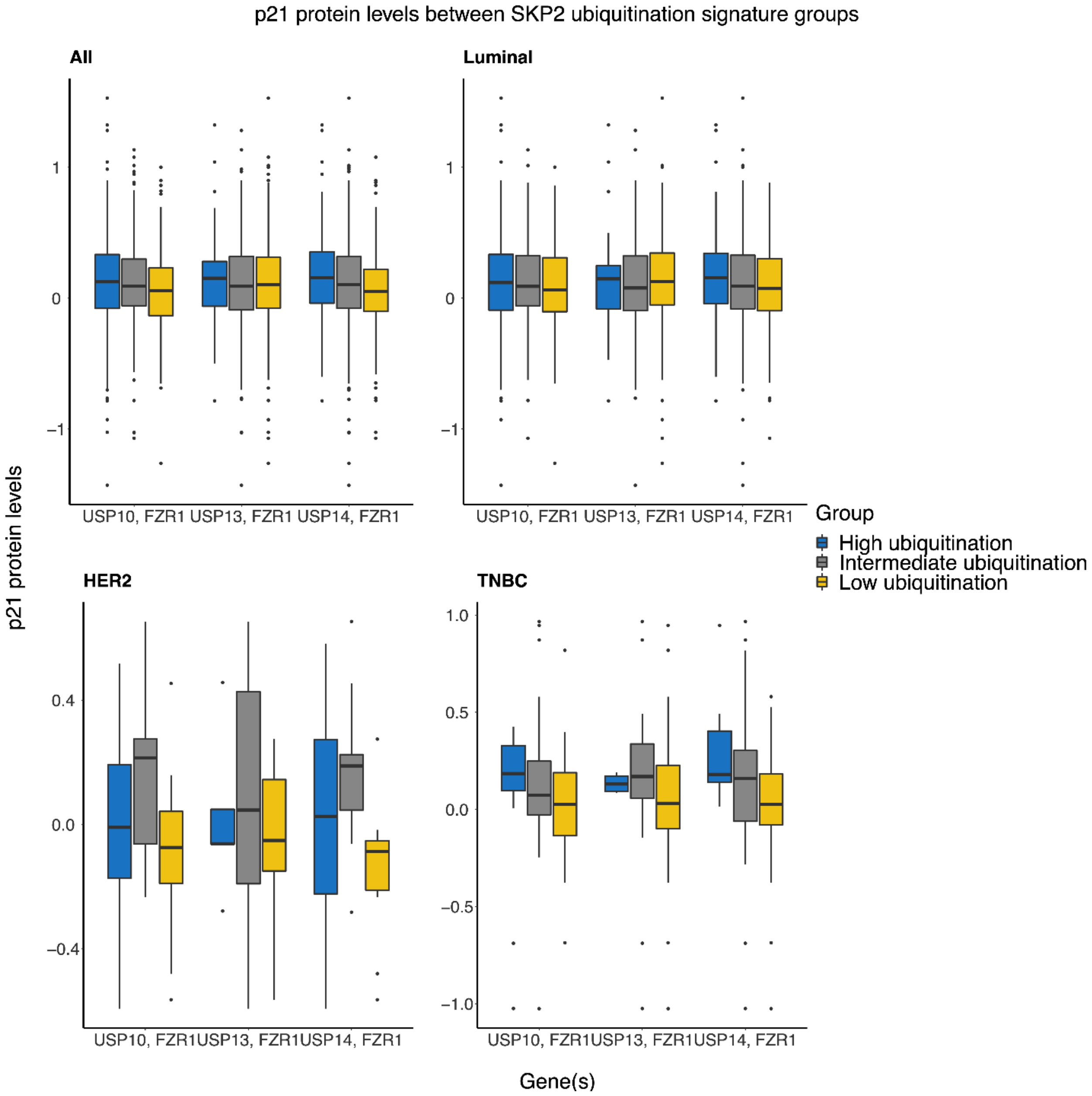

Fig. S2. **Comparison of p21 protein levels between ubiquitination gene-signature groups.** Analyses were performed on the data set in aggregate (n = 873) and on three major subtypes separately: luminal (n = 629), HER2 (n = 36), and triple-negative BC (TNBC) (n = 92). Comparison of p27 protein levels between SKP2 ubiquitination groups as defined by our signature (copy number). Subtypes are defined as in **Fig. 2**. *P*-values shown are for one-way ANOVA. Groups were determined as described in **Table 1**. The signature consisting of USP14 and FZR1 served as the negative control, as in **Fig. 2**.

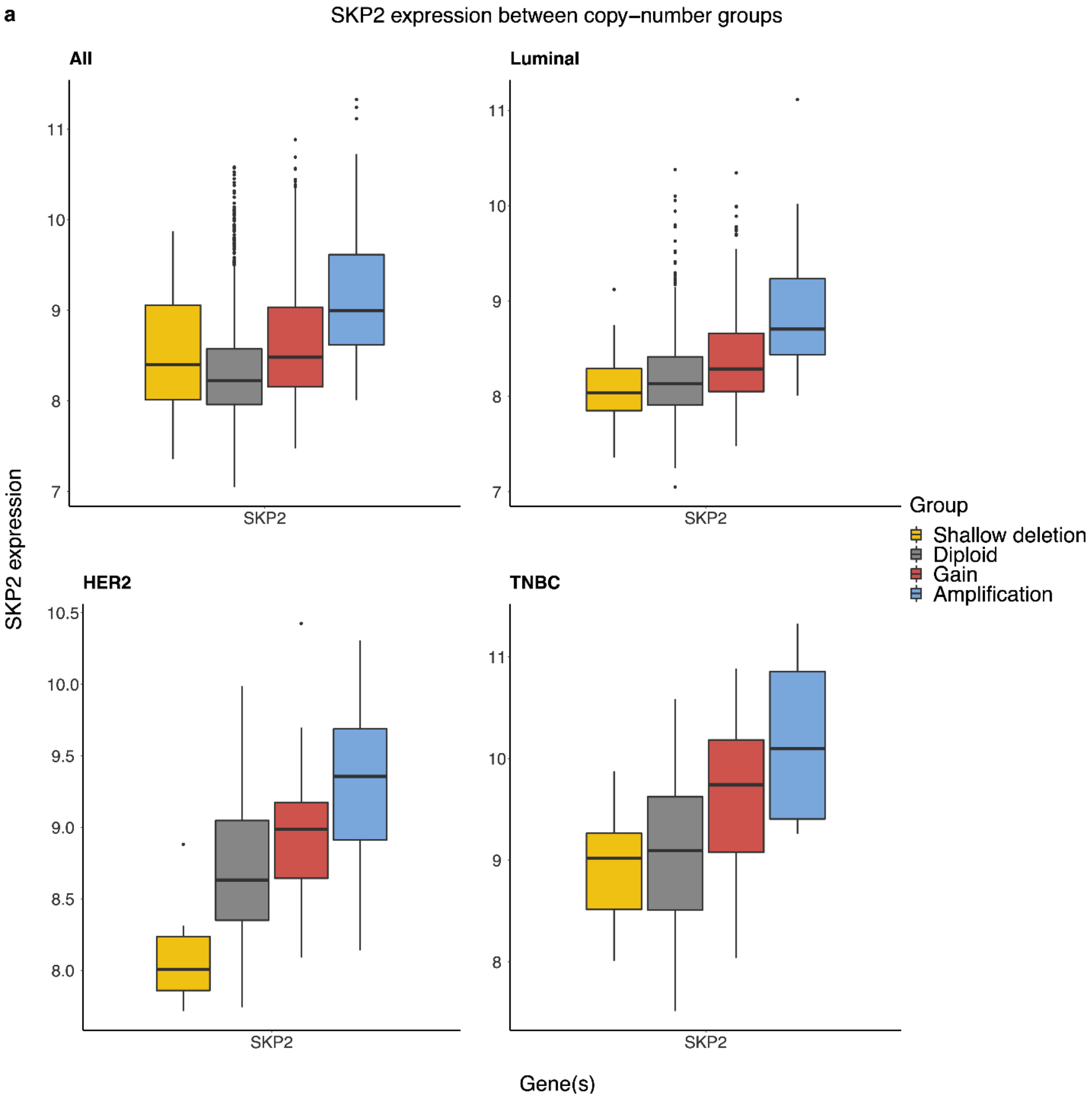

a
SKP2 expression between copy−number groups
All
Luminal
HER2
TNBC
SKP2 expression
11
10
9
8
7
10.5
10.0
9.5
9.0
8.5
8.0
SKP2
Gene(s)
Group
Shallow deletion
Diploid
Gain
Amplification

b

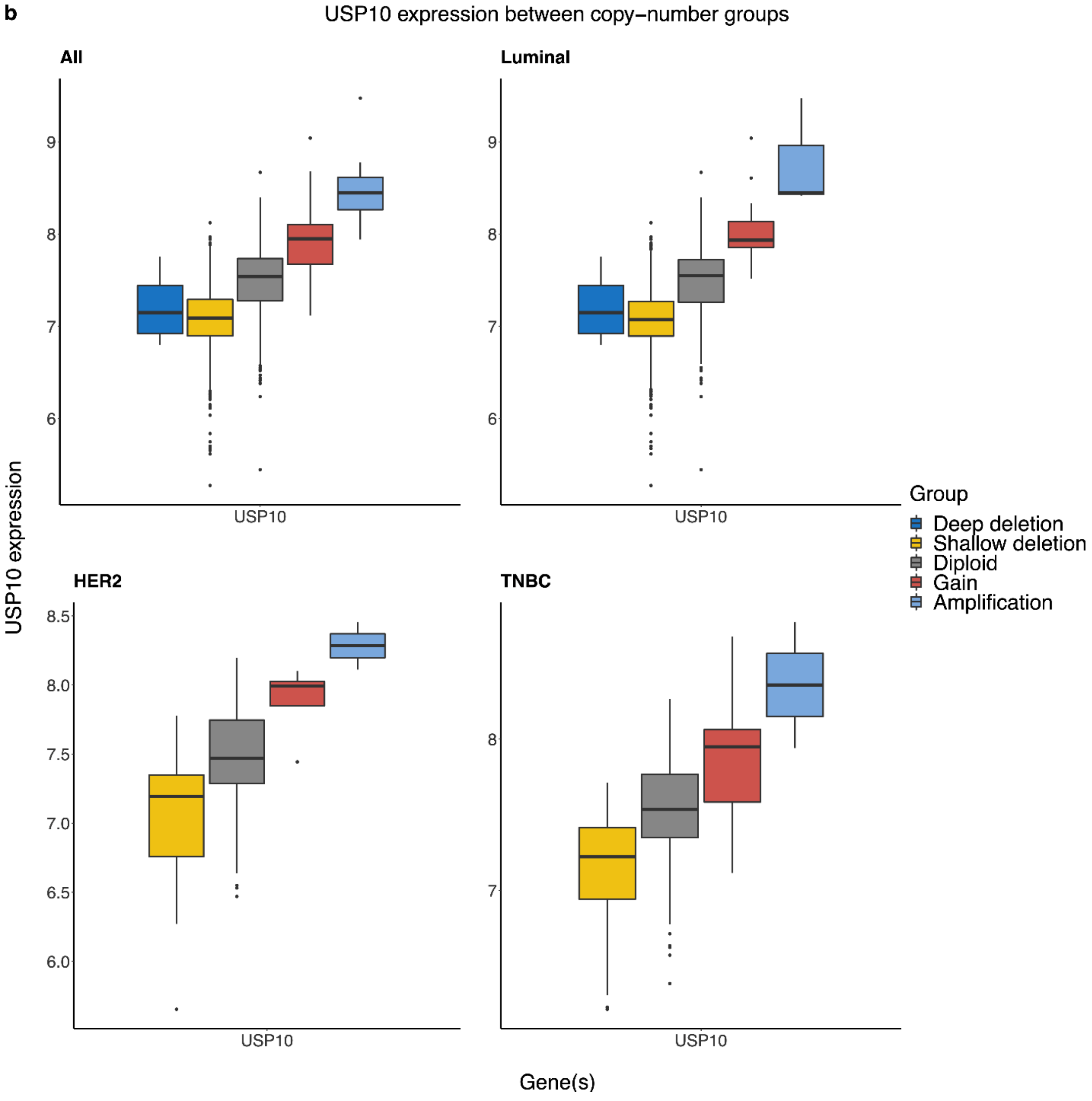
USP10 expression between copy−number groups
All
Luminal
HER2
TNBC
USP10 expression
USP10
Gene(s)
Group
Deep deletion
Shallow deletion
Diploid
Gain
Amplification
9
8
7
6
8.5
8.0
7.5
7.0
6.5
6.0

c

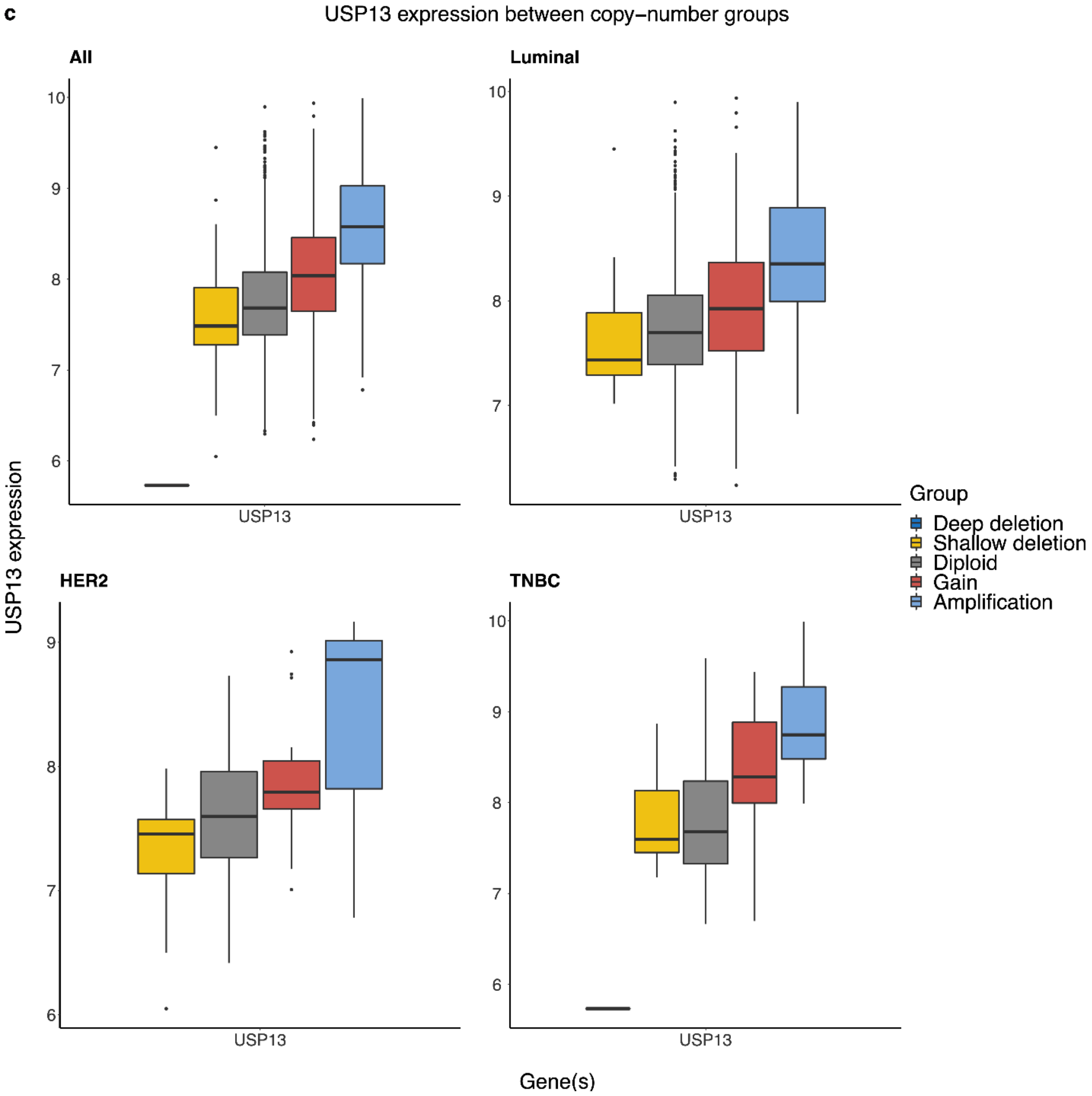
USP13 expression between copy−number groups
All
Luminal
HER2
TNBC
USP13 expression
USP13
Gene(s)
Group
Deep deletion
Shallow deletion
Diploid
Gain
Amplification
10
9
8
7
6

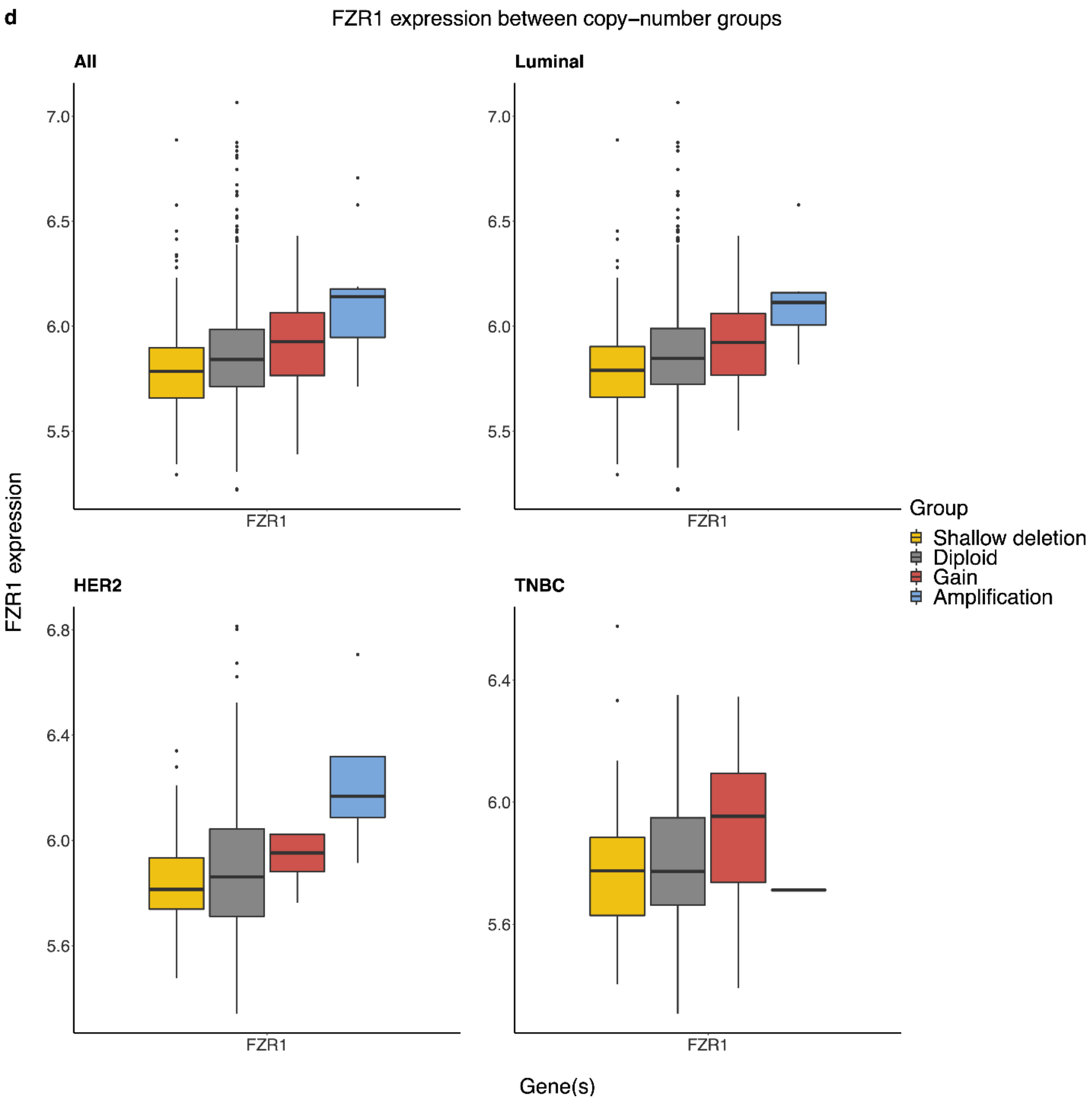

Figure S3. Association of expression with copy number in SKP2 (a), USP10 (b), USP13 (c), and FZR1 (d). Samples were grouped according to copy number–alteration (CAN) of the gene of interest as follows: deep deletion (CNA <= –2), shallow deletion (CNA <= –1), diploid (CNA = 0), gain (CNA = 1), and amplification (CNA >= 1). Analyses were performed on the data set in aggregate (*n* = 1,904) and on three major subtypes separately: luminal (*n* = 1,478), HER2 (*n* = 127), and TNBC (*n* = 299). Subtypes are defined as in **Fig. 2**.

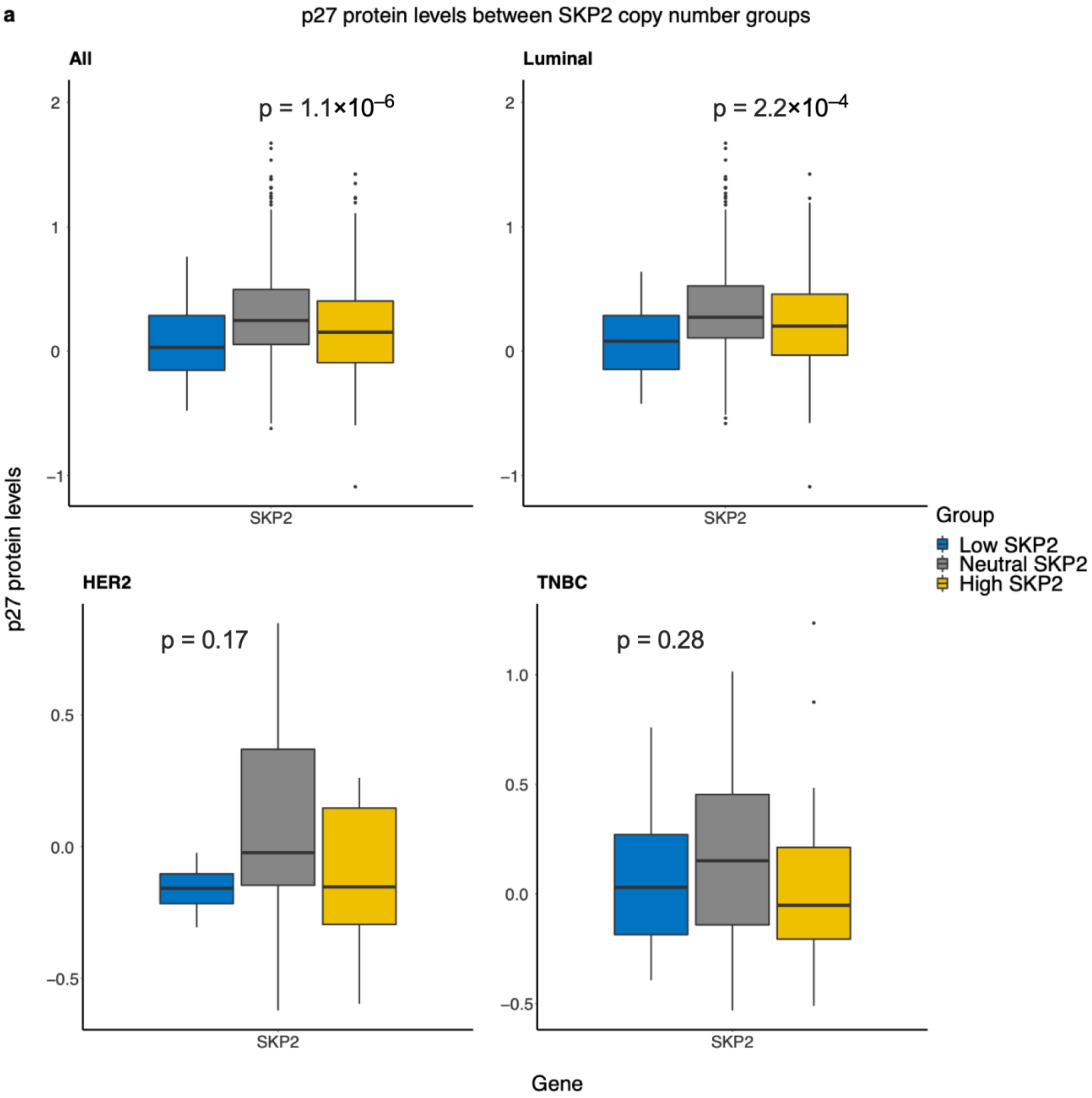
a
p27 protein levels between SKP2 copy number groups
All
p = 1.1×10–6
Luminal
p = 2.2×10–4
HER2
p = 0.17
TNBC
p = 0.28
p27 protein levels
SKP2
Gene
Group
Low SKP2
Neutral SKP2
High SKP2

b

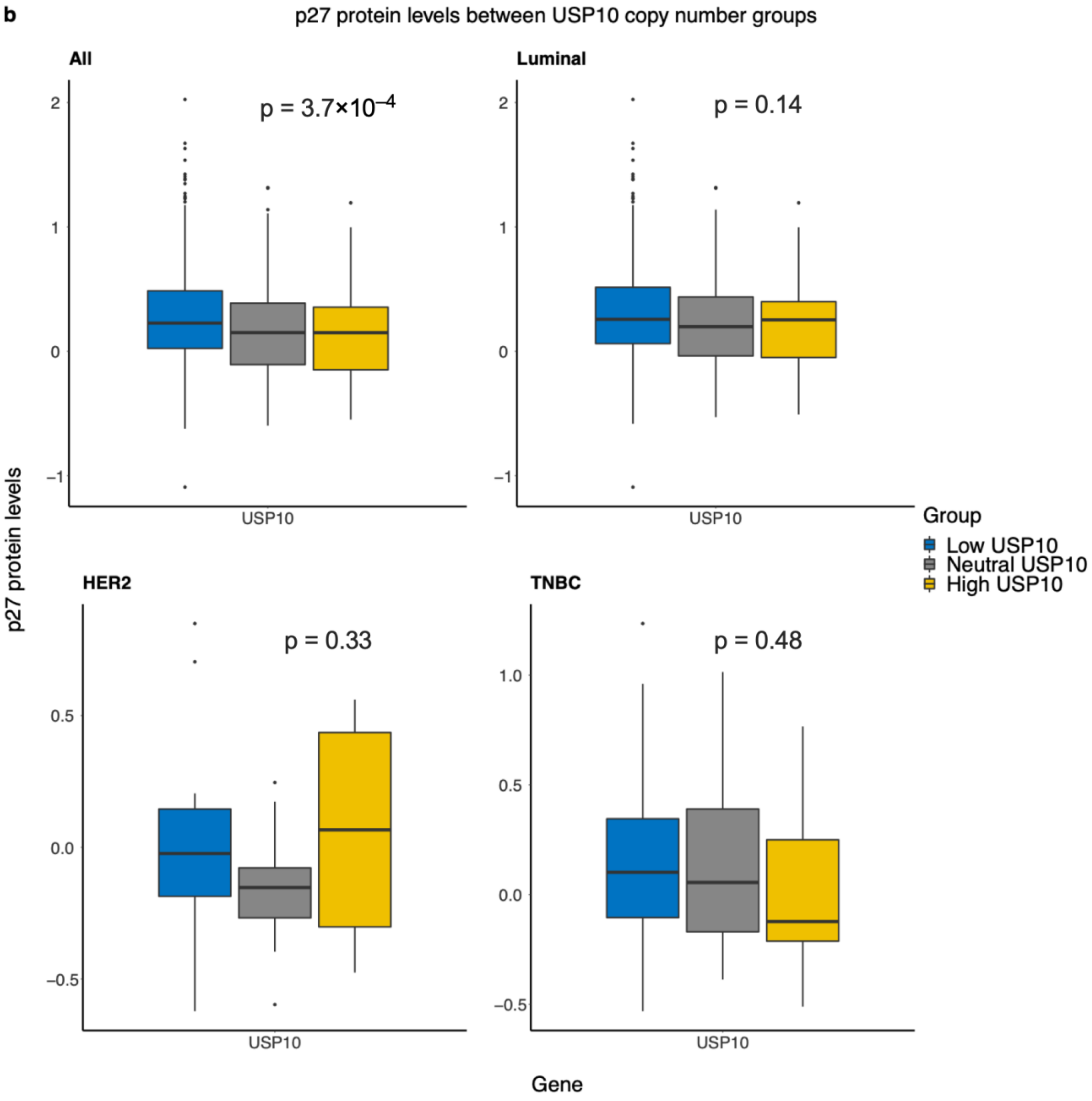
p27 protein levels between USP10 copy number groups
All
p = 3.7×10⁻⁴
Luminal
p = 0.14
HER2
p = 0.33
TNBC
p = 0.48
p27 protein levels
USP10
Gene
Group
Low USP10
Neutral USP10
High USP10

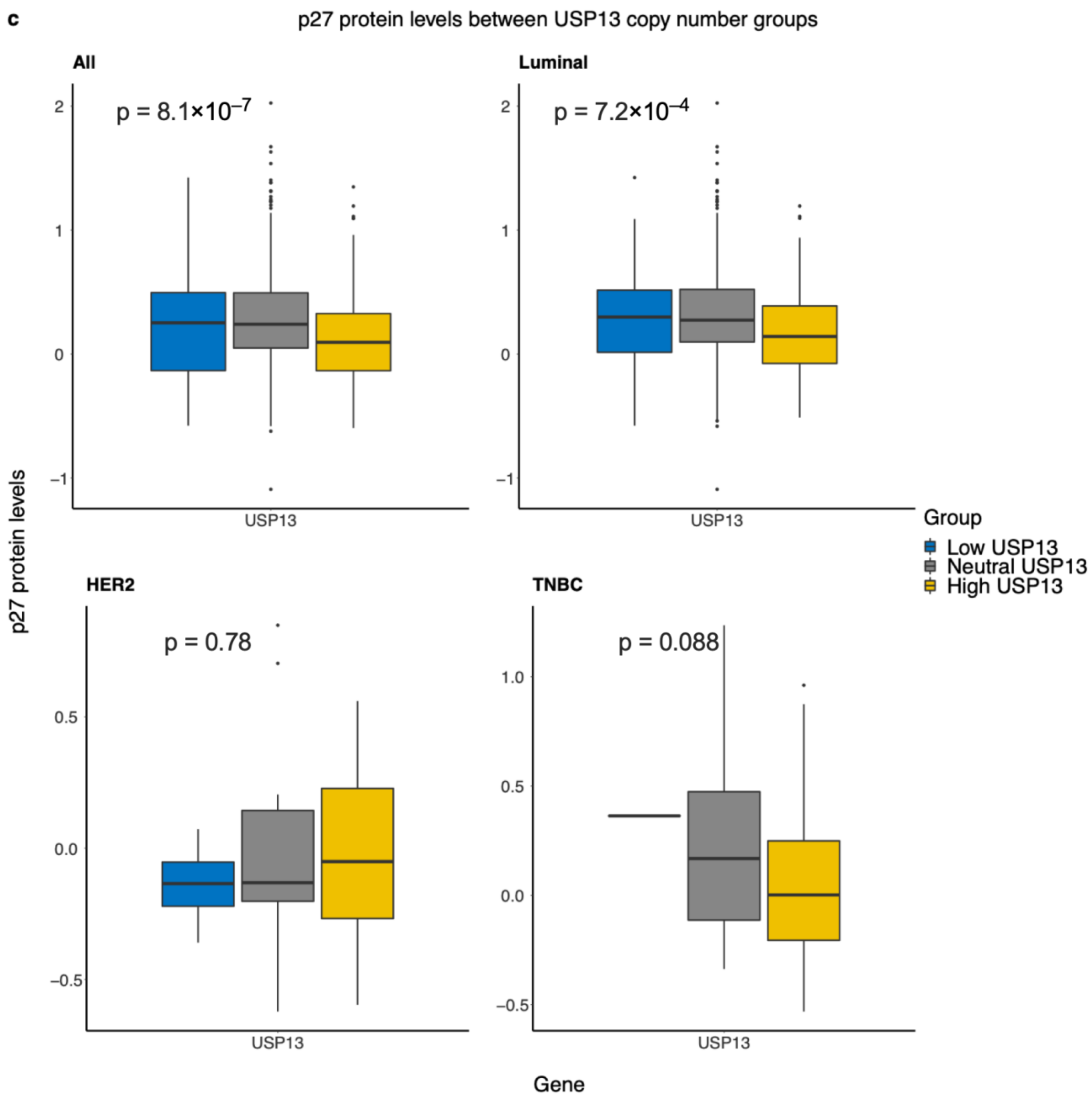
c
p27 protein levels between USP13 copy number groups
All
p = 8.1×10−7
Luminal
p = 7.2×10−4
HER2
p = 0.78
TNBC
p = 0.088
p27 protein levels
USP13
Gene
Group
Low USP13
Neutral USP13
High USP13

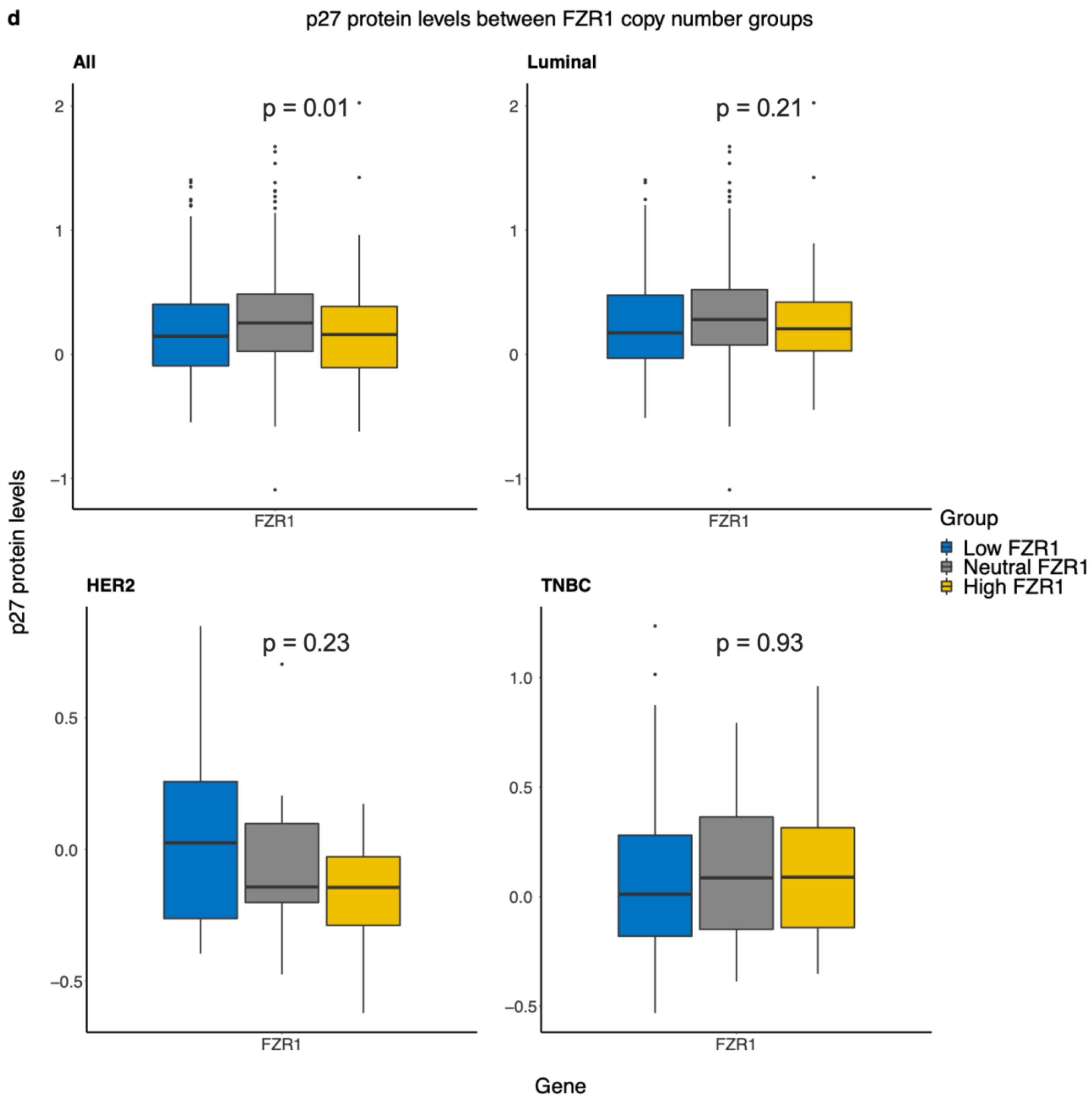

Fig. S4. **Comparison of p27 protein levels between copy-number groups of SKP2 (a), USP10 (b), USP13 (c), and FZR1 (d).** Analyses were performed on the data set in aggregate (*n* = 873) and on three major subtypes separately: luminal (*n* = 629), HER2 (*n* = 36), and triple-negative BC (TNBC) (*n* = 92.) Subtypes are defined as in **Fig. 2**. *P*-values shown are for one-way ANOVA. Samples were grouped according to copy-number alteration (CNA) of the gene of interest: samples with CNA levels < 0 were designated as "Low [gene of interest]"; those with CNA levels = 0 were designed as "Neutral [gene of interest]"; and those with CNA levels > 0 were designated as "High [gene of interest]." CNA levels were determined as described in the **Methods** section.

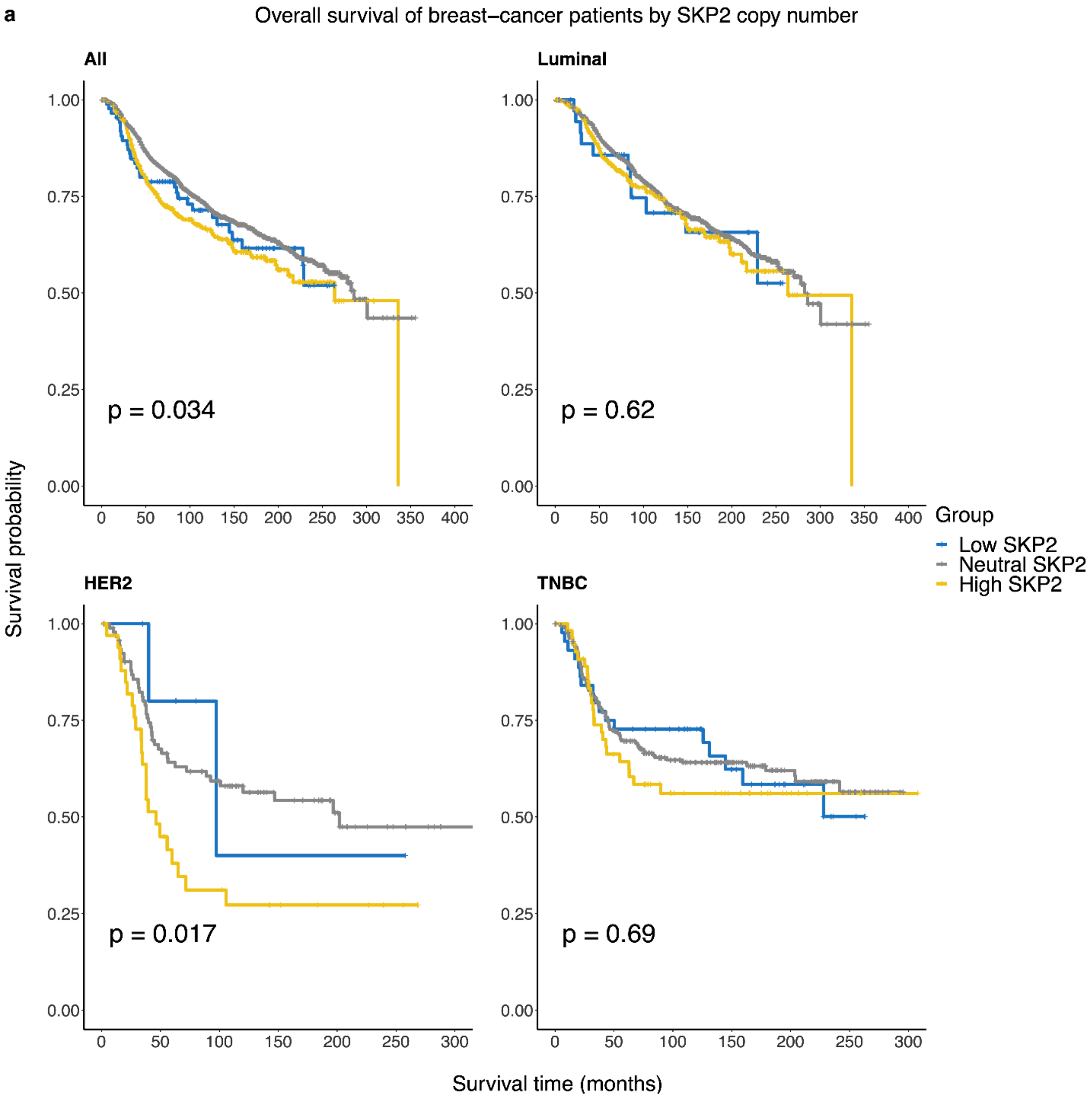
a
Overall survival of breast–cancer patients by SKP2 copy number
All
Luminal
HER2
TNBC
p = 0.034
p = 0.62
p = 0.017
p = 0.69
Survival probability
Survival time (months)
1.00
0.75
0.50
0.25
0.00
0
50
100
150
200
250
300
350
400
Group
Low SKP2
Neutral SKP2
High SKP2

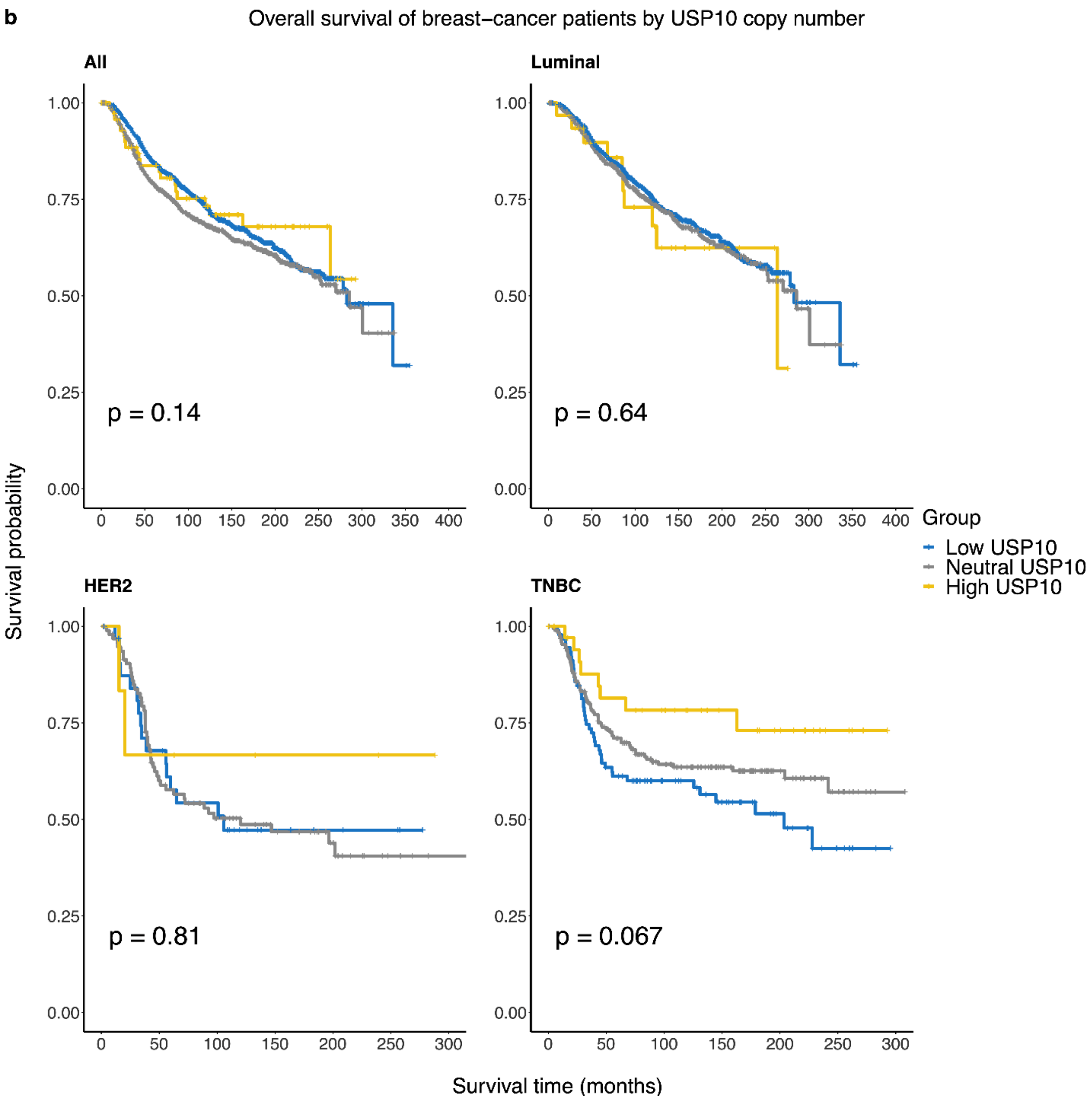
b
Overall survival of breast−cancer patients by USP10 copy number
All
Luminal
HER2
TNBC
p = 0.14
p = 0.64
p = 0.81
p = 0.067
Survival probability
Survival time (months)
Group
Low USP10
Neutral USP10
High USP10

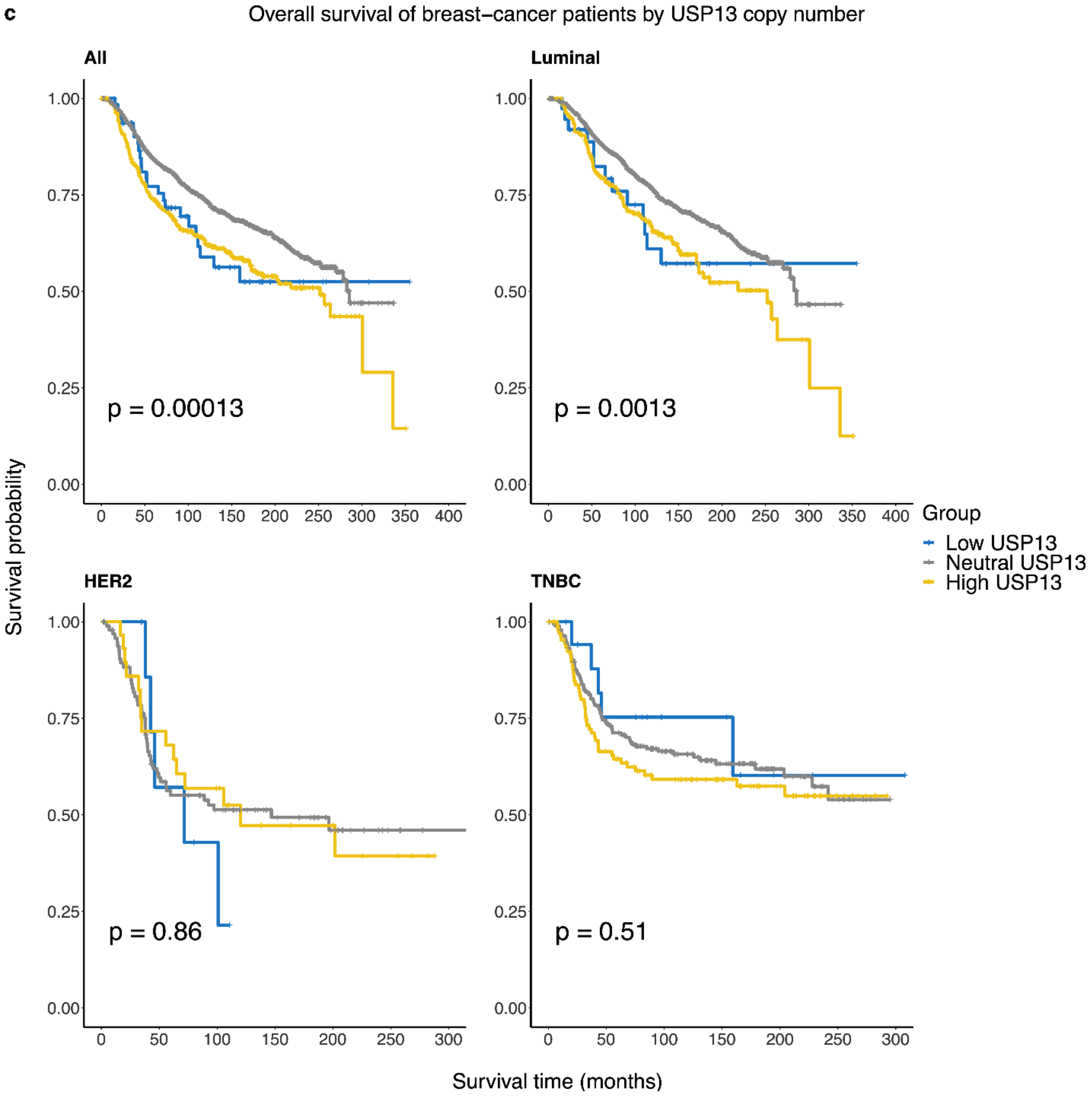
c
Overall survival of breast−cancer patients by USP13 copy number
All
Luminal
HER2
TNBC
p = 0.00013
p = 0.0013
p = 0.86
p = 0.51
1.00
0.75
0.50
0.25
0.00
0
50
100
150
200
250
300
350
400
Survival probability
Survival time (months)
Group
Low USP13
Neutral USP13
High USP13

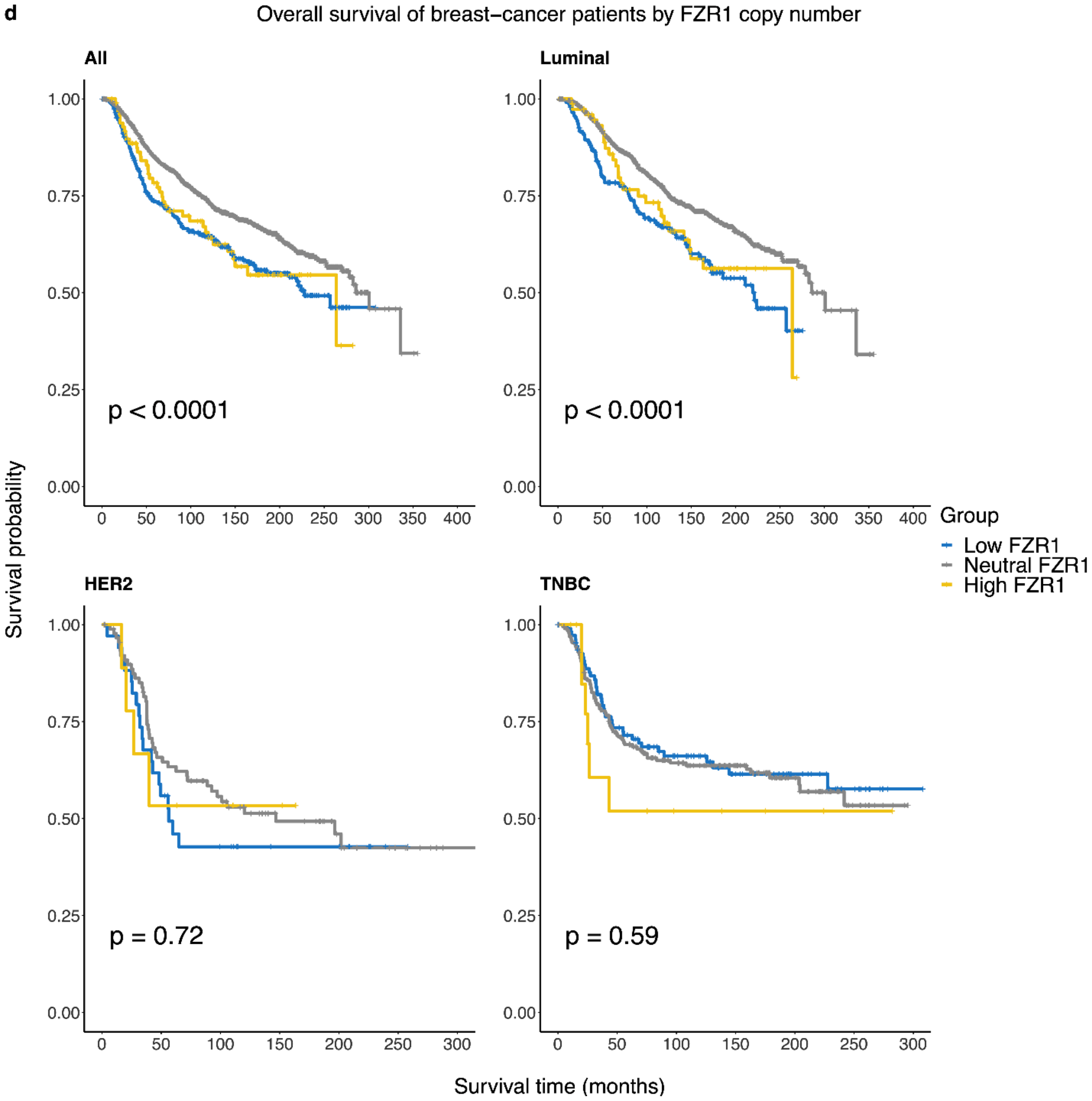

Fig. S5. Differences in overall survival between the SKP2 (a), USP10 (b), USP13 (c), or FZR1 (d) copy-number (CN) groups. Analyses were performed on the data set in aggregate (n = 1,981) and on three major subtypes separately: luminal (n = 1,527), HER2 (n = 134), and TNBC (n = 320). Subtypes are defined as in **Fig. 2**. The p-values shown were calculated using the log-rank test. Samples were grouped by copy number–alteration levels as described in **Figure S4**. Descriptive statistics are given in **Table S7**.

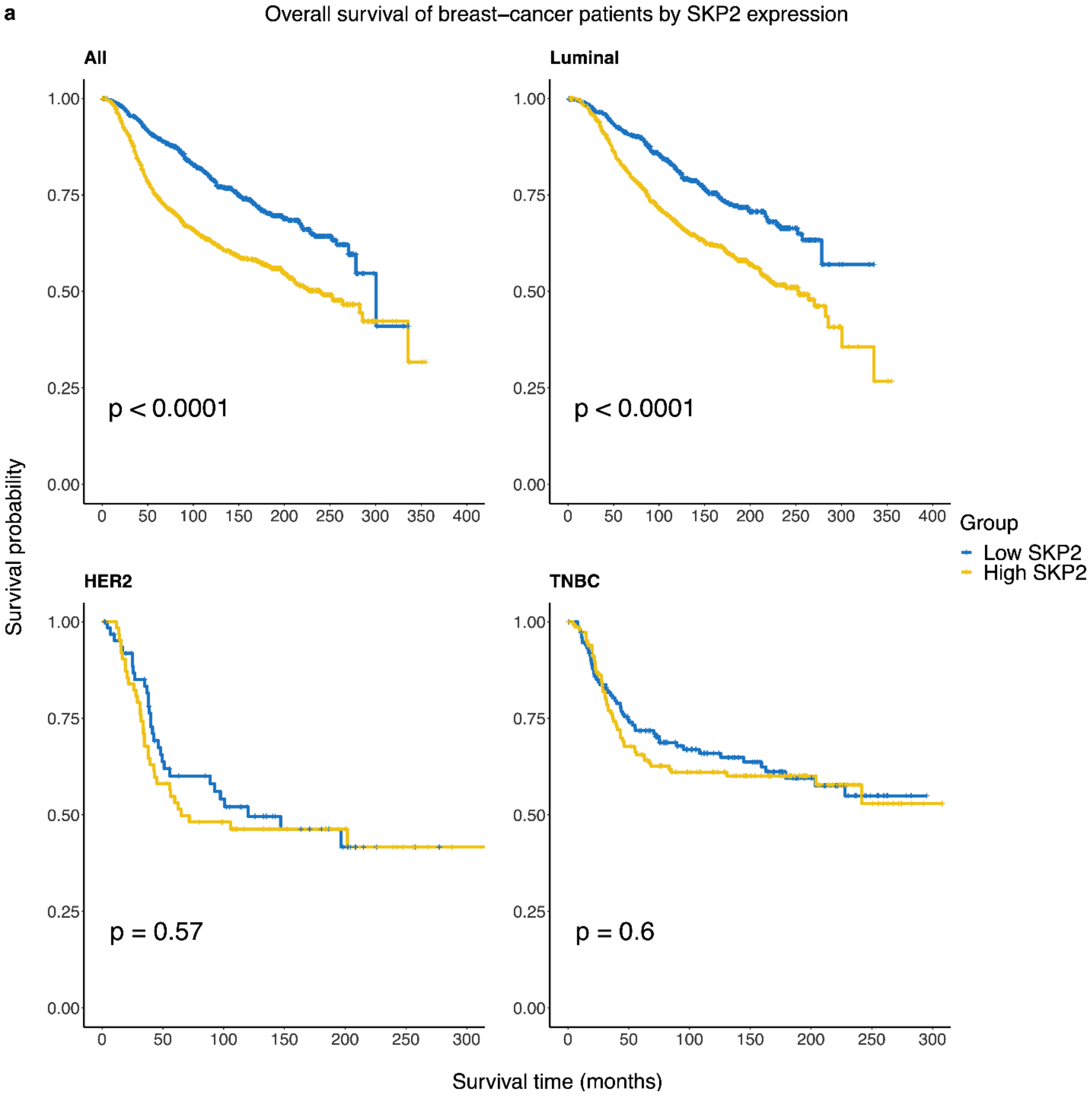
a
Overall survival of breast–cancer patients by SKP2 expression
All
Luminal
HER2
TNBC
Survival probability
1.00
0.75
0.50
0.25
0.00
0
50
100
150
200
250
300
350
400
p < 0.0001
p < 0.0001
p = 0.57
p = 0.6
Group
Low SKP2
High SKP2
Survival time (months)

b

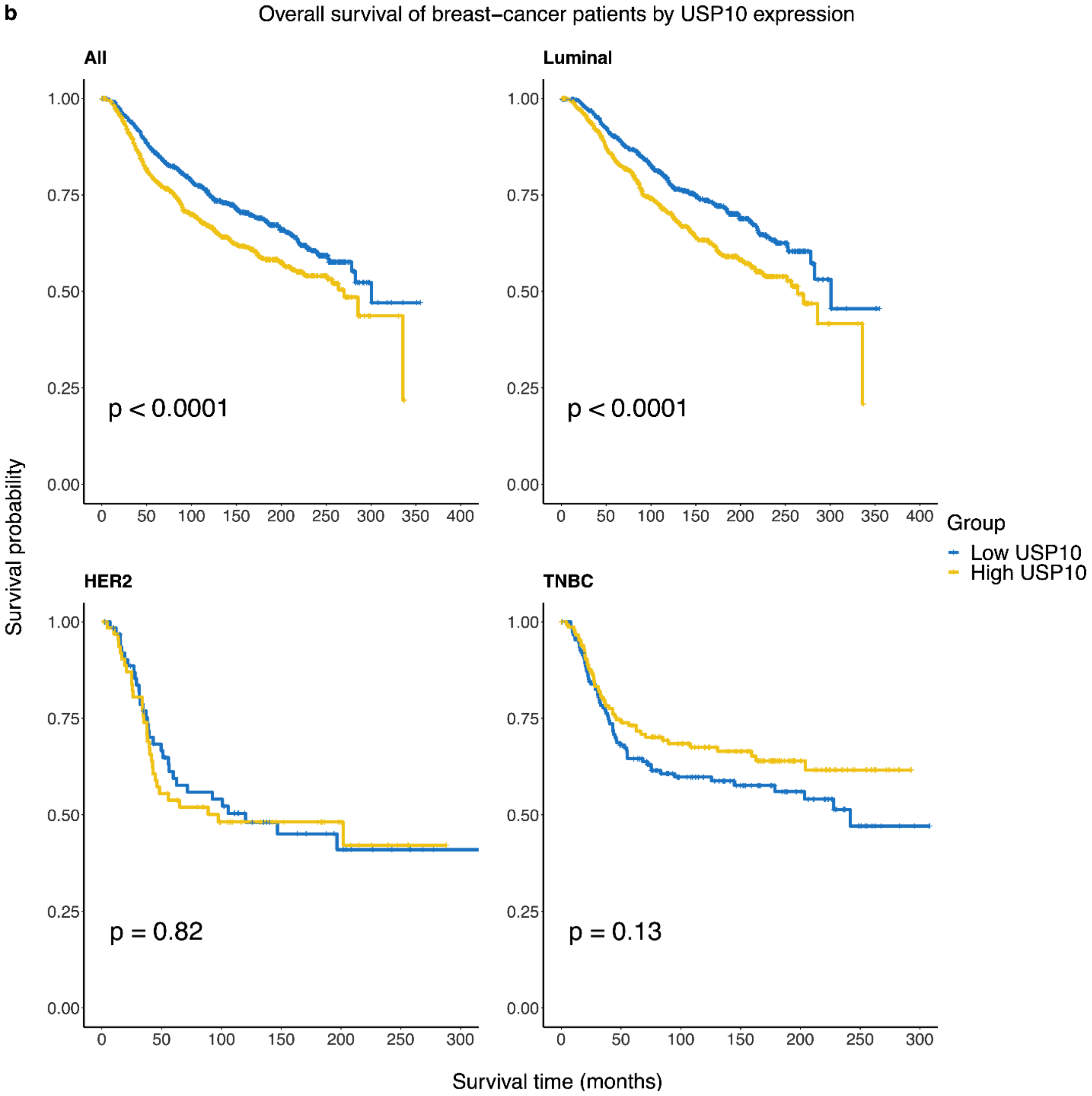

Overall survival of breast−cancer patients by USP10 expression
All
Luminal
HER2
TNBC
Survival probability
1.00
0.75
0.50
0.25
0.00
p < 0.0001
p < 0.0001
p = 0.82
p = 0.13
0
50
100
150
200
250
300
350
400
Survival time (months)
Group
Low USP10
High USP10

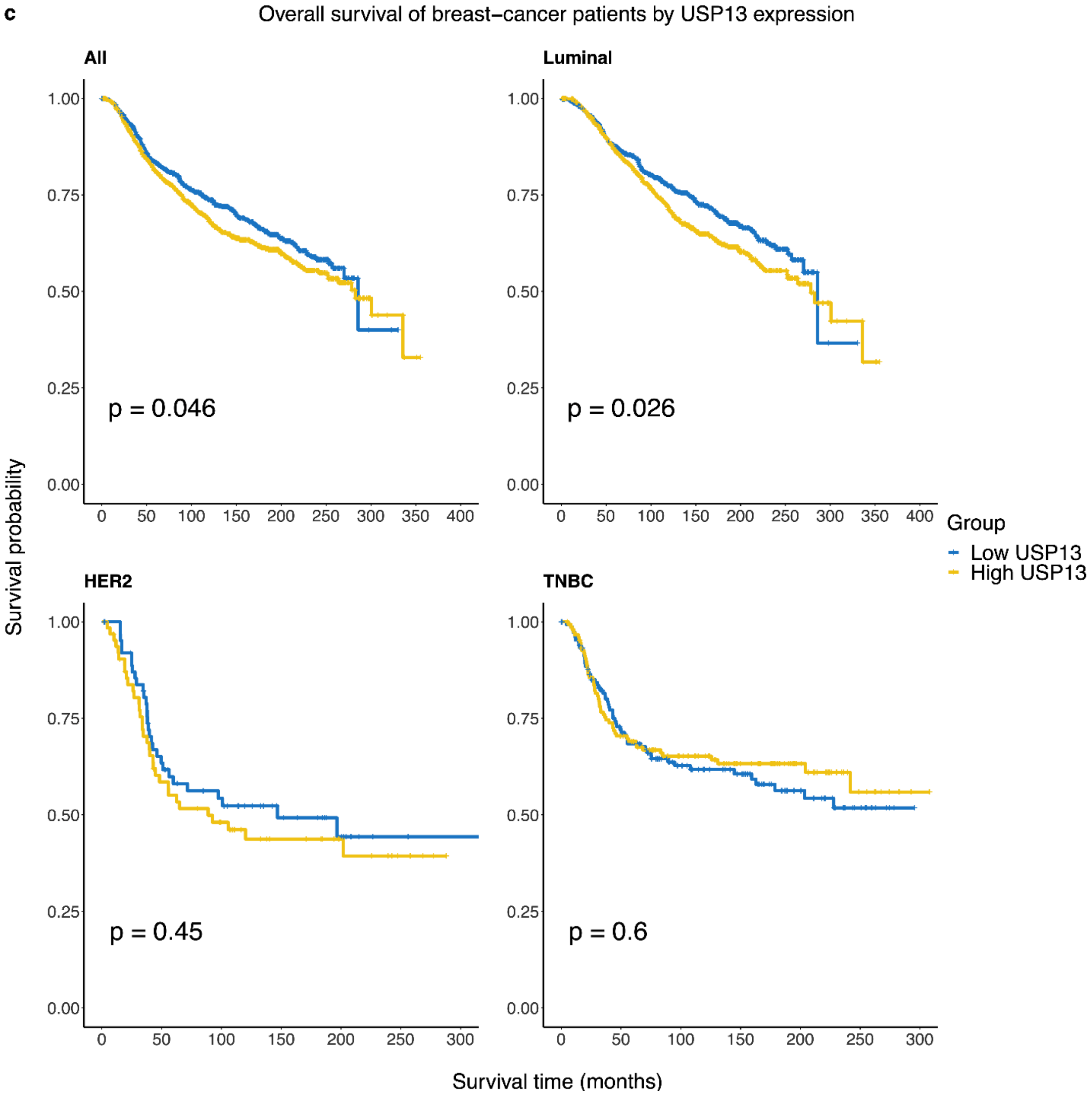
c
Overall survival of breast−cancer patients by USP13 expression
All
Luminal
HER2
TNBC
Survival probability
1.00
0.75
0.50
0.25
0.00
p = 0.046
p = 0.026
p = 0.45
p = 0.6
0
50
100
150
200
250
300
350
400
Survival time (months)
Group
Low USP13
High USP13

d

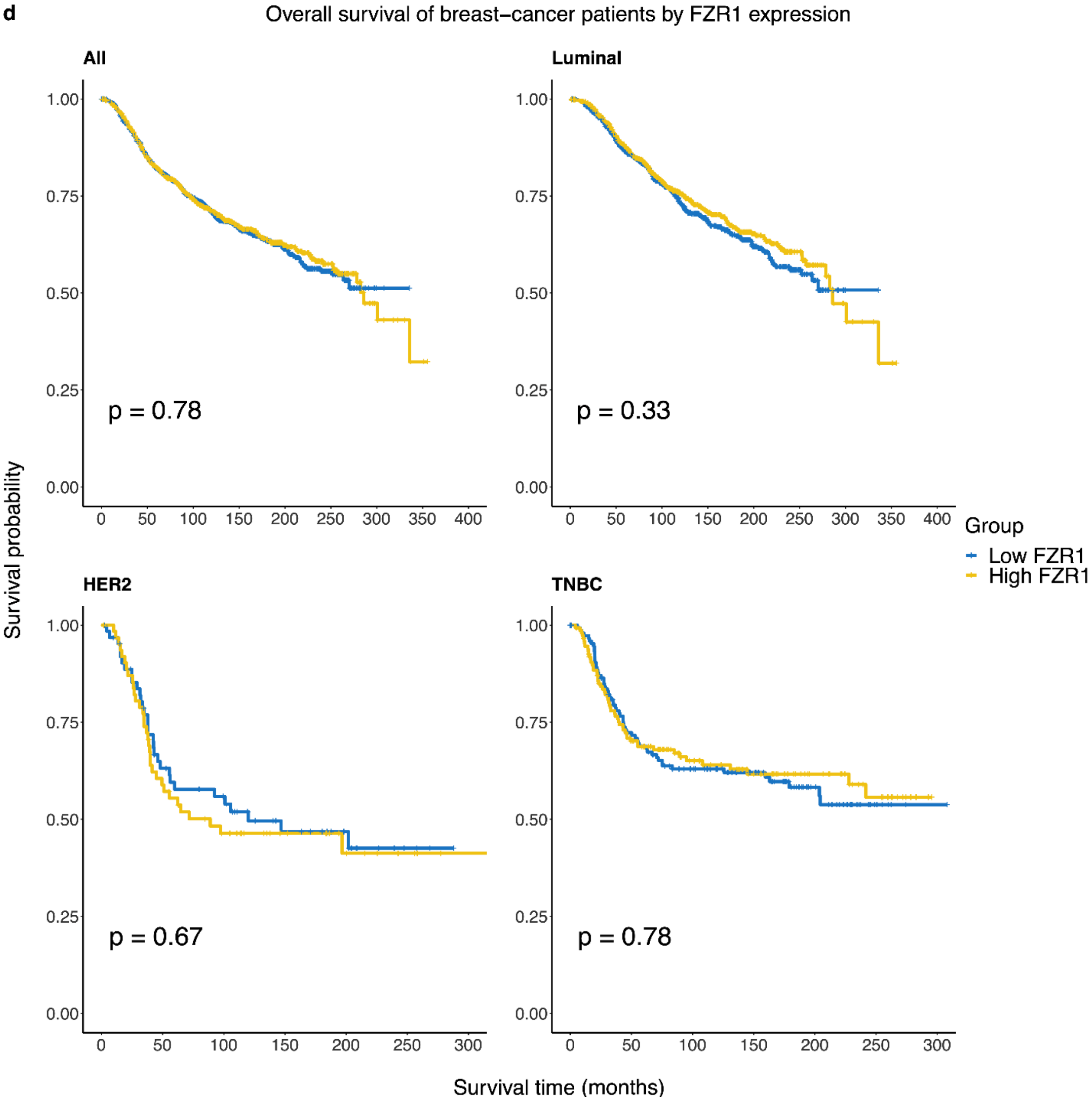

Fig. S6. Differences in overall survival between the SKP2 (a), USP10 (b), USP13 (c), or FZR1 (d) expression groups. Analyses were performed on the data set in aggregate (n = 1,904) and on three major subtypes separately: luminal (n = 1,478), HER2 (n = 127), and TNBC (n = 299). Subtypes are defined as in **Fig. 2**. Samples were grouped according to expression of the gene of interest: samples with expression <= median expression of the gene of interest in their subtype were designated as “Low [gene of interest]”, and those with expression > median expression of the gene of interest in their subtype were designated as “High [gene of interest].” The *p*-values shown were calculated using the log-rank test. Descriptive statistics are given in **Table S8**.

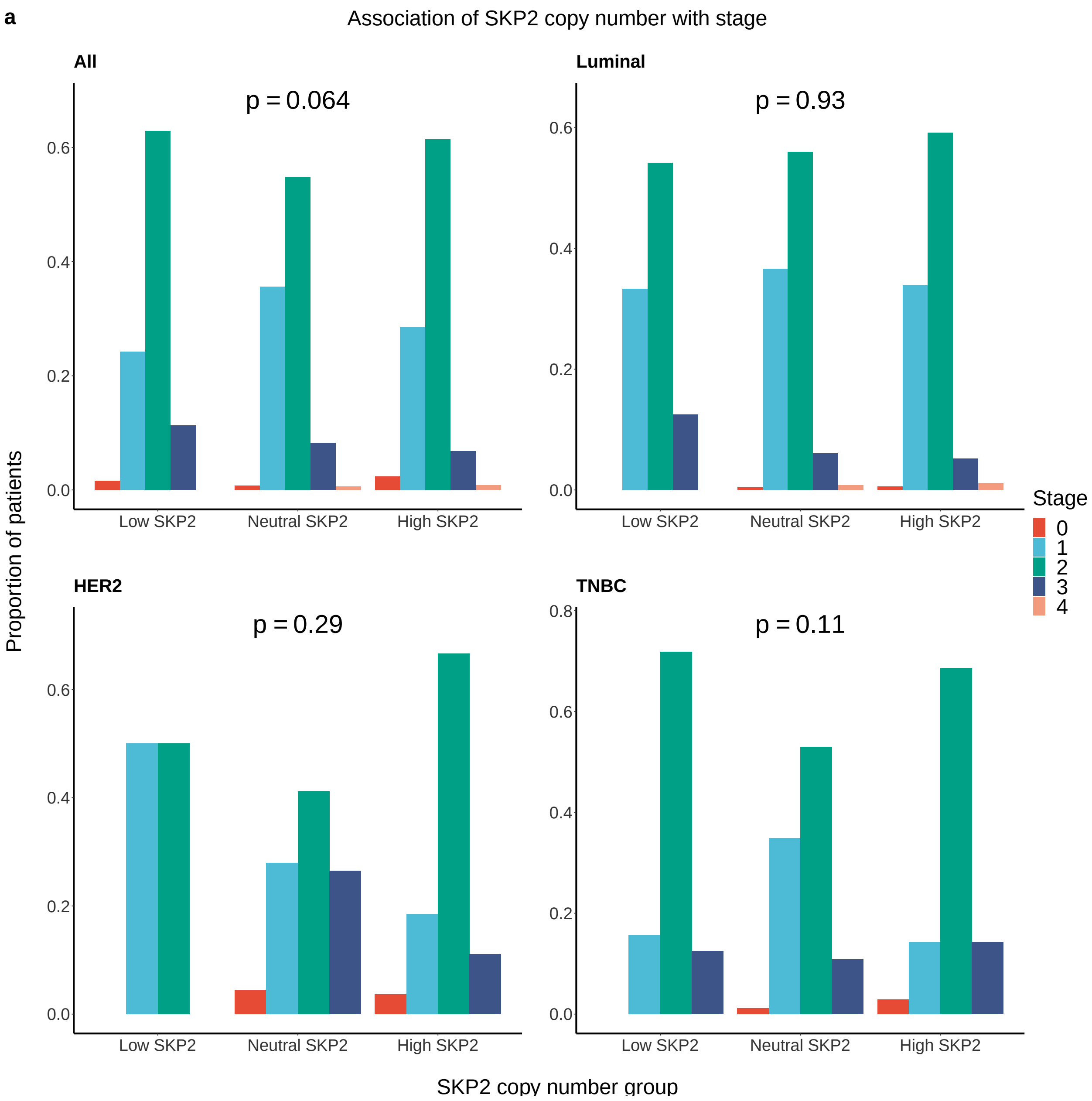
a
Association of SKP2 copy number with stage
All
p = 0.064
Luminal
p = 0.93
HER2
p = 0.29
TNBC
p = 0.11
Proportion of patients
0.0
0.2
0.4
0.6
0.8
Low SKP2
Neutral SKP2
High SKP2
SKP2 copy number group
Stage
0
1
2
3
4

b

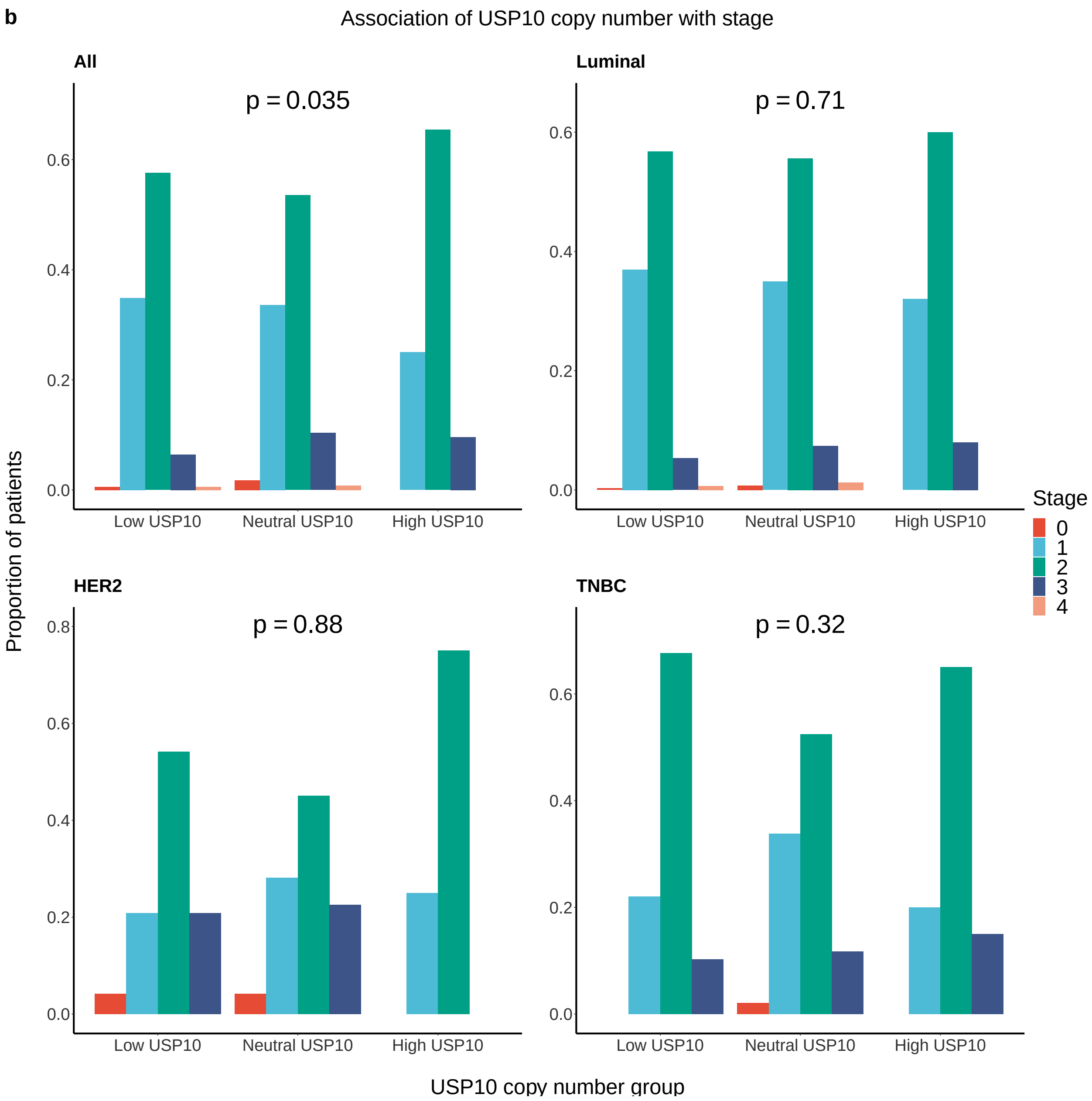
Association of USP10 copy number with stage
All
p = 0.035
Luminal
p = 0.71
HER2
p = 0.88
TNBC
p = 0.32
0.0
0.2
0.4
0.6
0.8
Low USP10
Neutral USP10
High USP10
Proportion of patients
USP10 copy number group
Stage
0
1
2
3
4

c

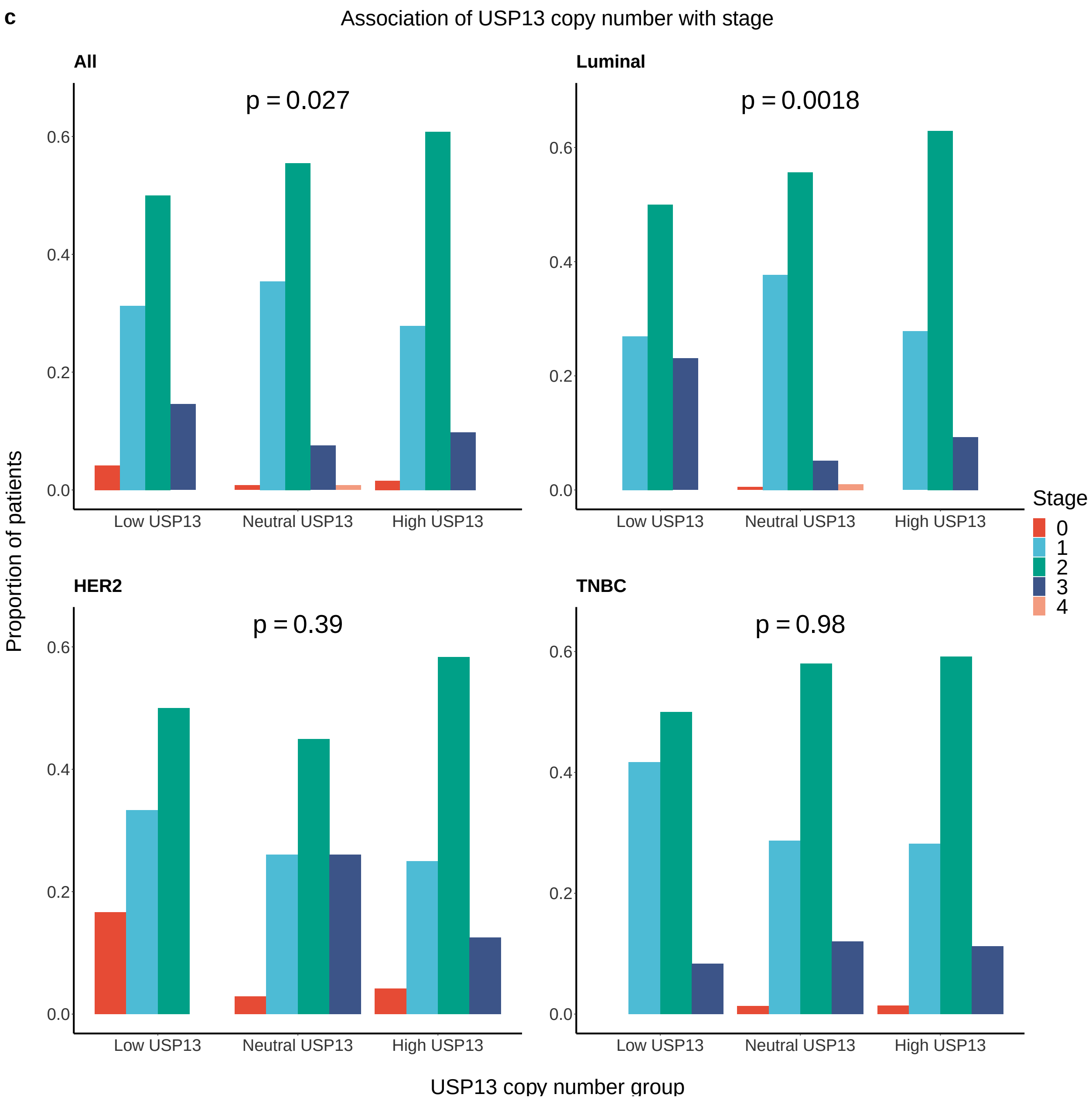
Association of USP13 copy number with stage
All
p = 0.027
Luminal
p = 0.0018
HER2
p = 0.39
TNBC
p = 0.98
0.0
0.2
0.4
0.6
Low USP13
Neutral USP13
High USP13
Proportion of patients
USP13 copy number group
Stage
0
1
2
3
4

d

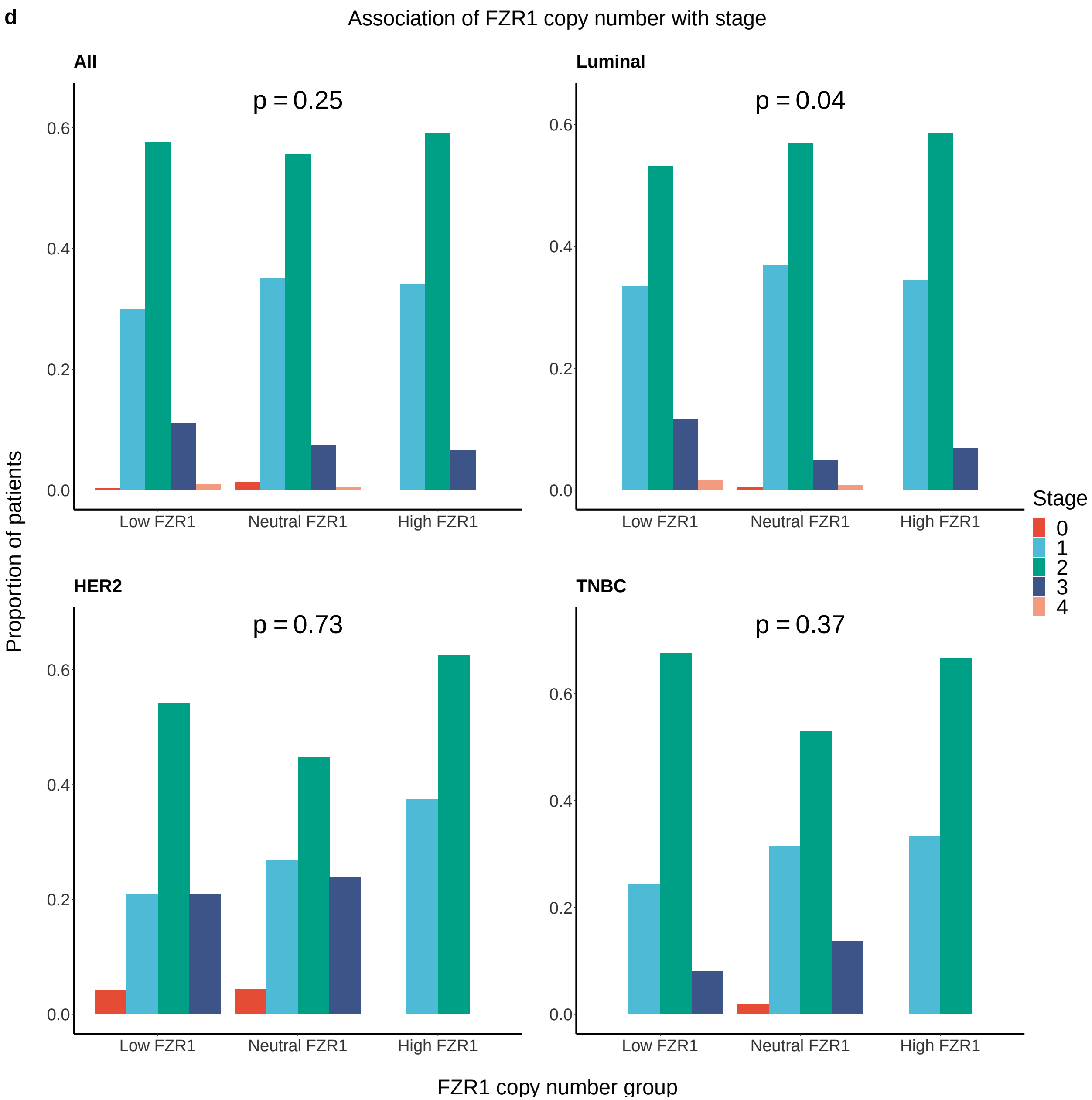

Fig. S7. The distribution of patients at a given tumor stage by copy-number group of SKP2 (a), USP10 (b), USP13 (c), or FZR1 (d). Analyses were performed on the data set in aggregate (*n* = 1,552) and on three major subtypes separately: luminal (*n* = 1,169), HER2 (*n* = 99), and TNBC (*n* = 233). Subtypes are defined as in **Fig. 2**. The chi-square test was used to determine the significance of association between ubiquitination group and tumor stage. Samples were grouped by copy number–alteration levels as described in **Figure S4**.

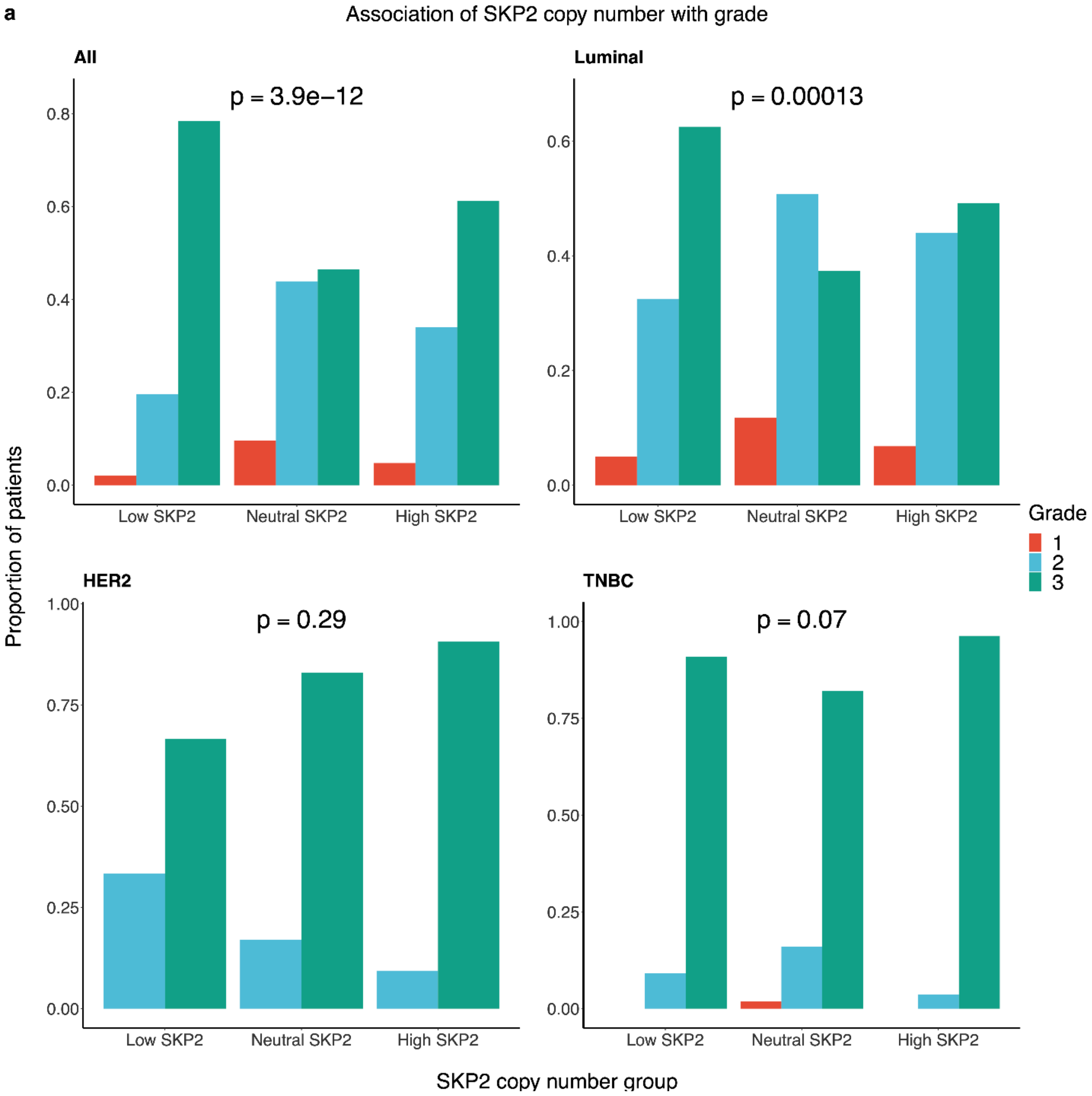
a
Association of SKP2 copy number with grade
All
p = 3.9e−12
Luminal
p = 0.00013
HER2
p = 0.29
TNBC
p = 0.07
Proportion of patients
Low SKP2
Neutral SKP2
High SKP2
SKP2 copy number group
Grade
1
2
3

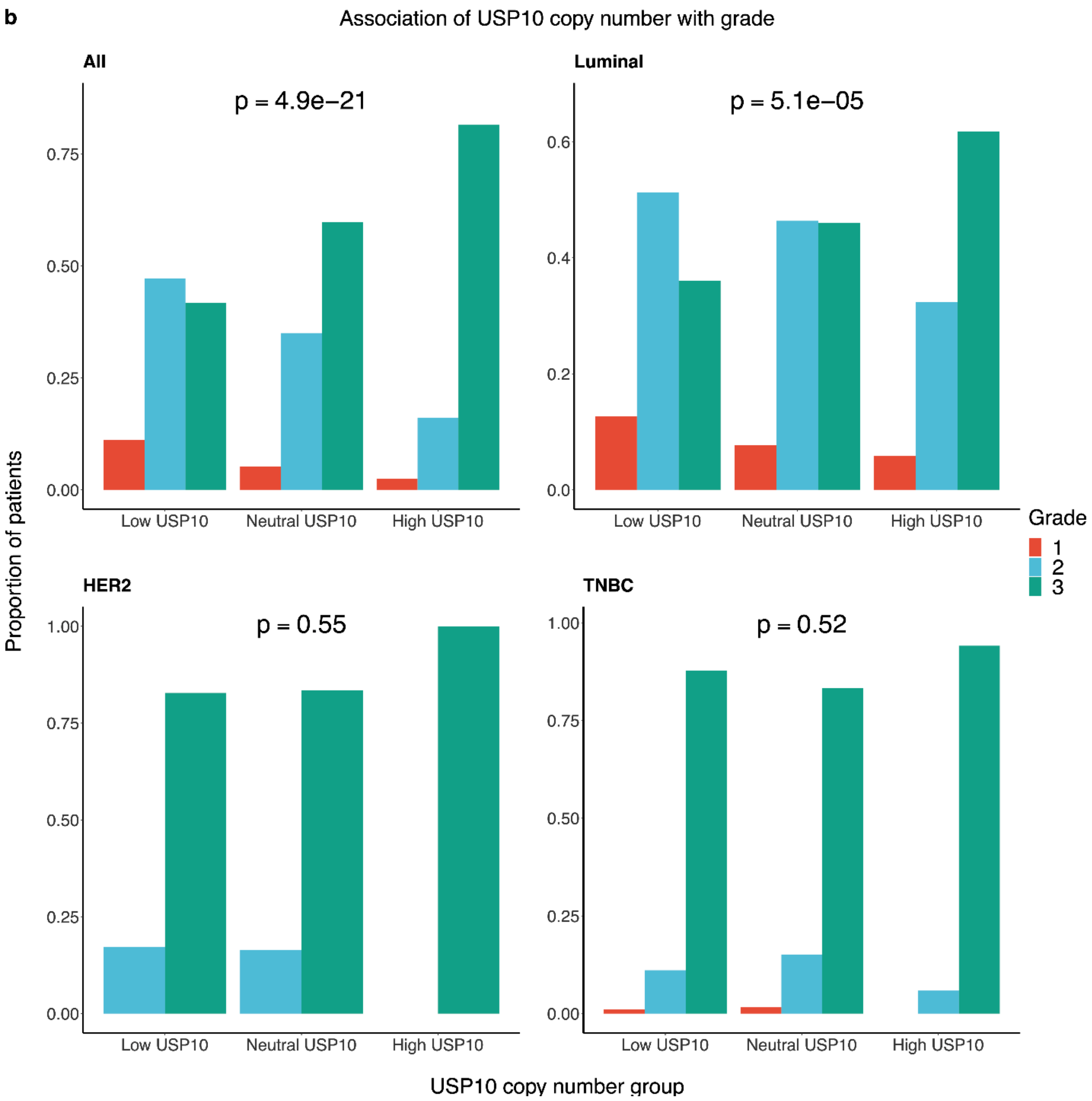

b
Association of USP10 copy number with grade
All
p = 4.9e−21
Luminal
p = 5.1e−05
HER2
p = 0.55
TNBC
p = 0.52
Proportion of patients
0.00
0.25
0.50
0.75
1.00
0.0
0.2
0.4
0.6
Low USP10
Neutral USP10
High USP10
Grade
1
2
3
USP10 copy number group

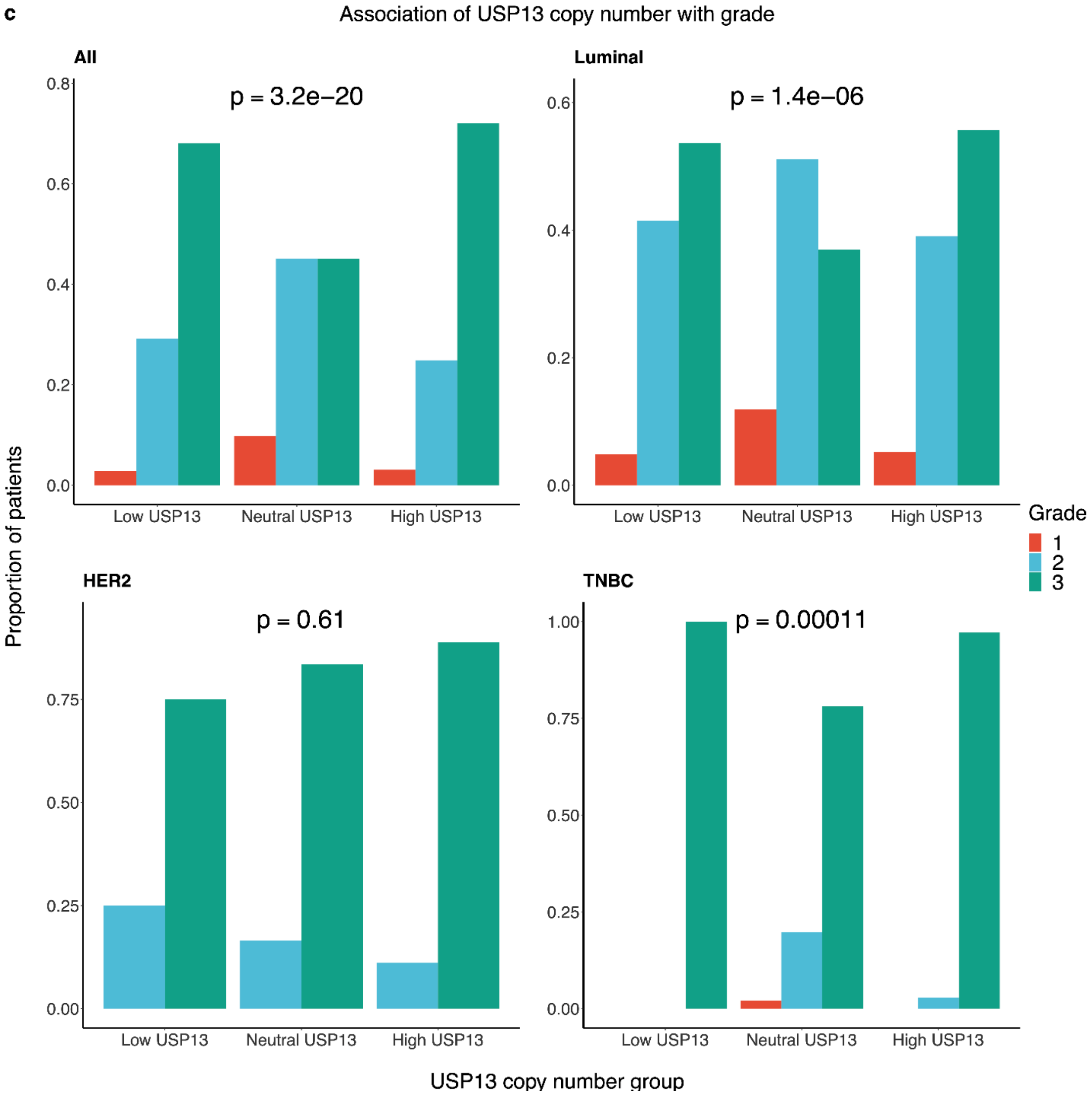
c
Association of USP13 copy number with grade
All
p = 3.2e−20
Luminal
p = 1.4e−06
HER2
p = 0.61
TNBC
p = 0.00011
Proportion of patients
Low USP13
Neutral USP13
High USP13
Grade
1
2
3
USP13 copy number group

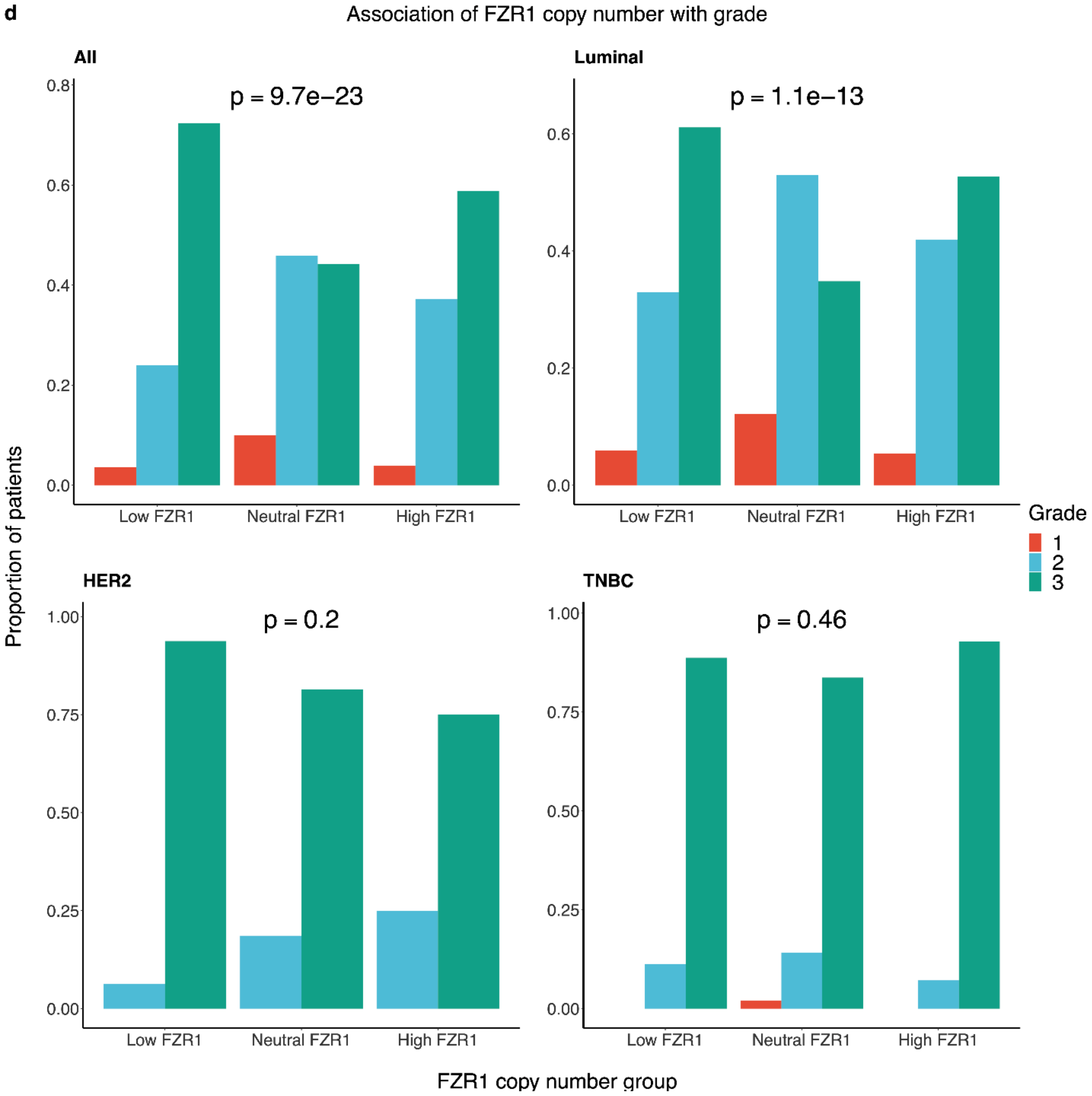

Fig. S8. The distribution of patients with a given tumor grade by copy-number group of SKP2 (a), USP10 (b), USP13 (c), or FZR1 (d). Analyses were performed on the data set in aggregate (*n* = 2,072) and on three major subtypes separately: luminal (*n* = 1,560), HER2 (*n* = 126), and TNBC (*n* = 310). Subtypes are defined as in **Fig. 2**. The chi-square test was used to determine the significance of association between ubiquitination group and tumor stage. Samples were grouped by copy number–alteration levels as described in **Figure S4**.

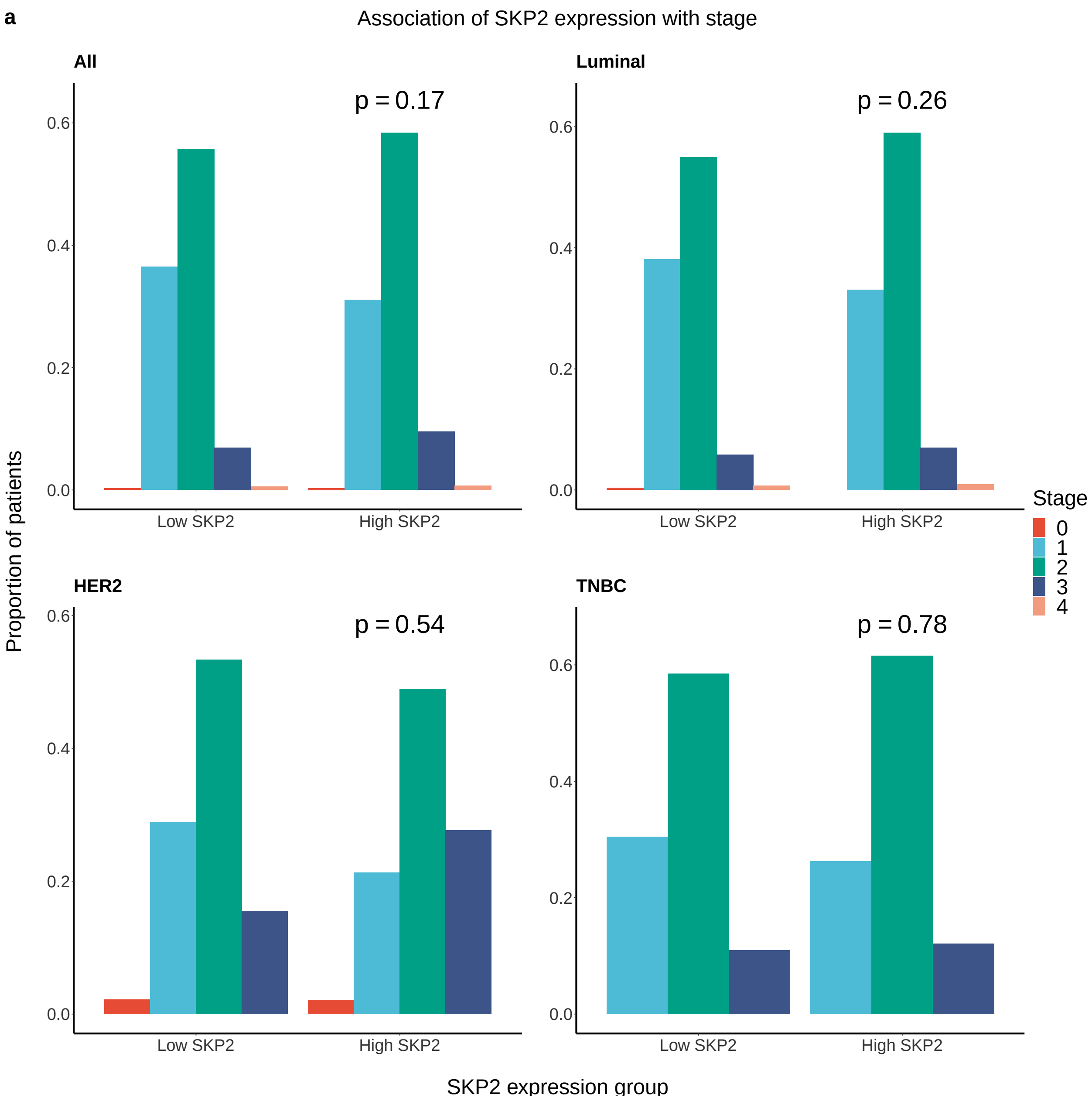
a
Association of SKP2 expression with stage
All
p = 0.17
Luminal
p = 0.26
HER2
p = 0.54
TNBC
p = 0.78
0.0
0.2
0.4
0.6
Low SKP2
High SKP2
Proportion of patients
SKP2 expression group
Stage
0
1
2
3
4

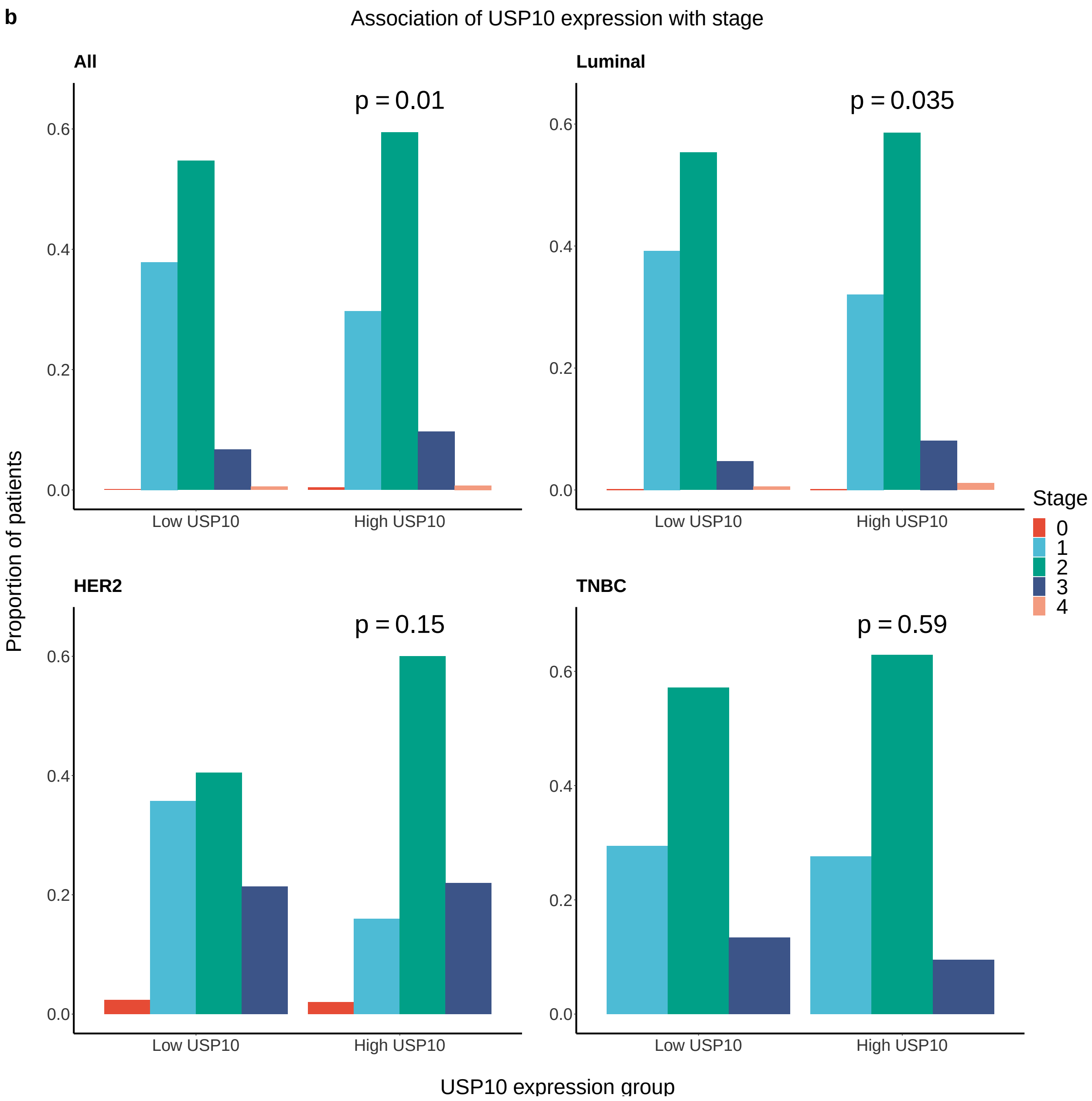
b
Association of USP10 expression with stage
All
Luminal
HER2
TNBC
p = 0.01
p = 0.035
p = 0.15
p = 0.59
Proportion of patients
0.0
0.2
0.4
0.6
Low USP10
High USP10
USP10 expression group
Stage
0
1
2
3
4

c

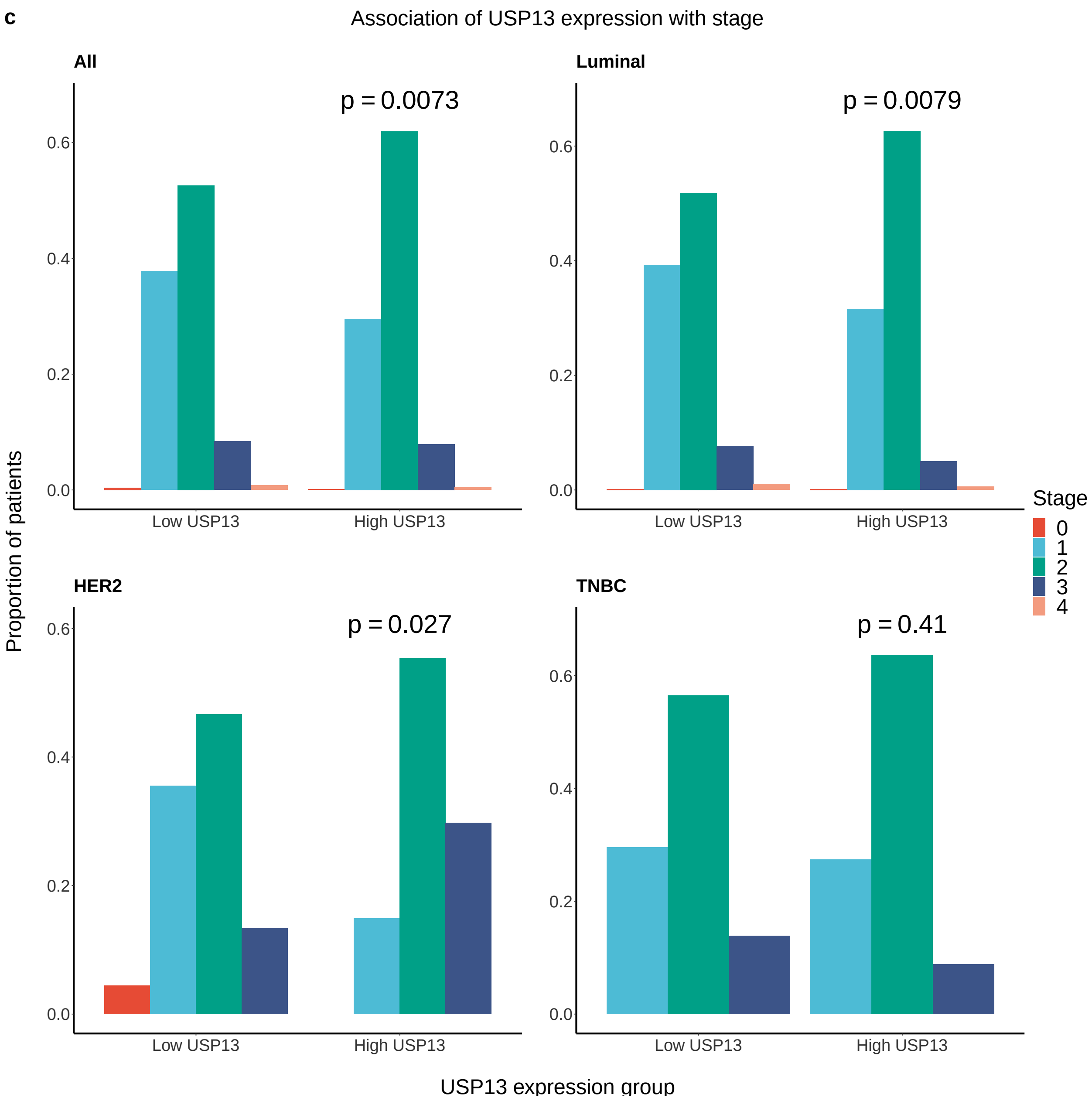

Association of USP13 expression with stage
All
p = 0.0073
Luminal
p = 0.0079
HER2
p = 0.027
TNBC
p = 0.41
0.0
0.2
0.4
0.6
Low USP13
High USP13
Proportion of patients
USP13 expression group
Stage
0
1
2
3
4

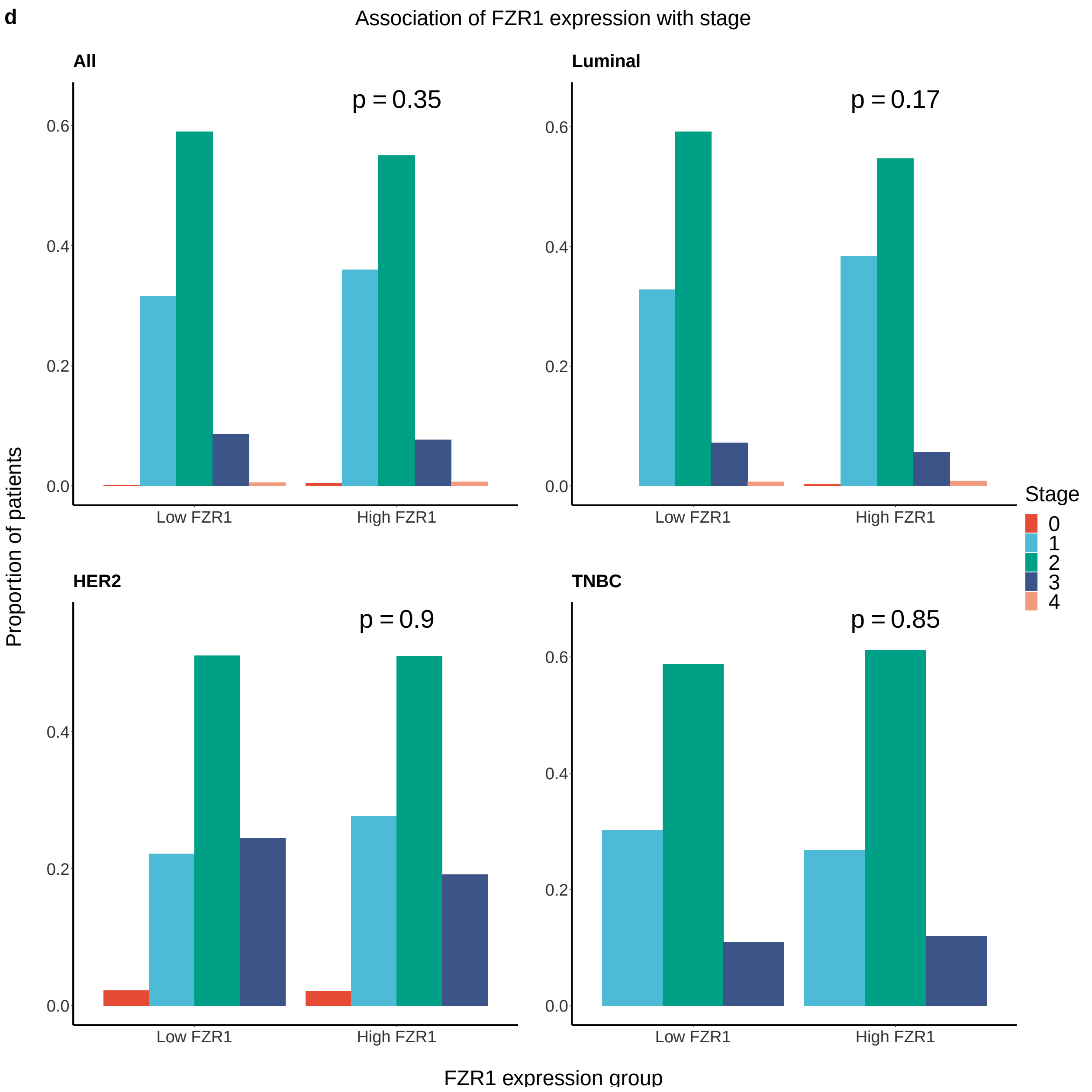

Figure S9. **The distribution of patients at a given tumor stage by expression of SKP2 (a), USP10 (b), USP13 (c), or FZR1 (d)**. Analyses were performed on the data set in aggregate (*n* = 1,403) and on three major subtypes separately: luminal (*n* = 1,094), HER2 (*n* = 92), and TNBC (*n* = 217). Subtypes are defined as in **Fig. 2**. The chi-square test was used to determine the significance of association between ubiquitination group and tumor stage. Samples were grouped by expression of the gene of interest as described in **Figure S6**.

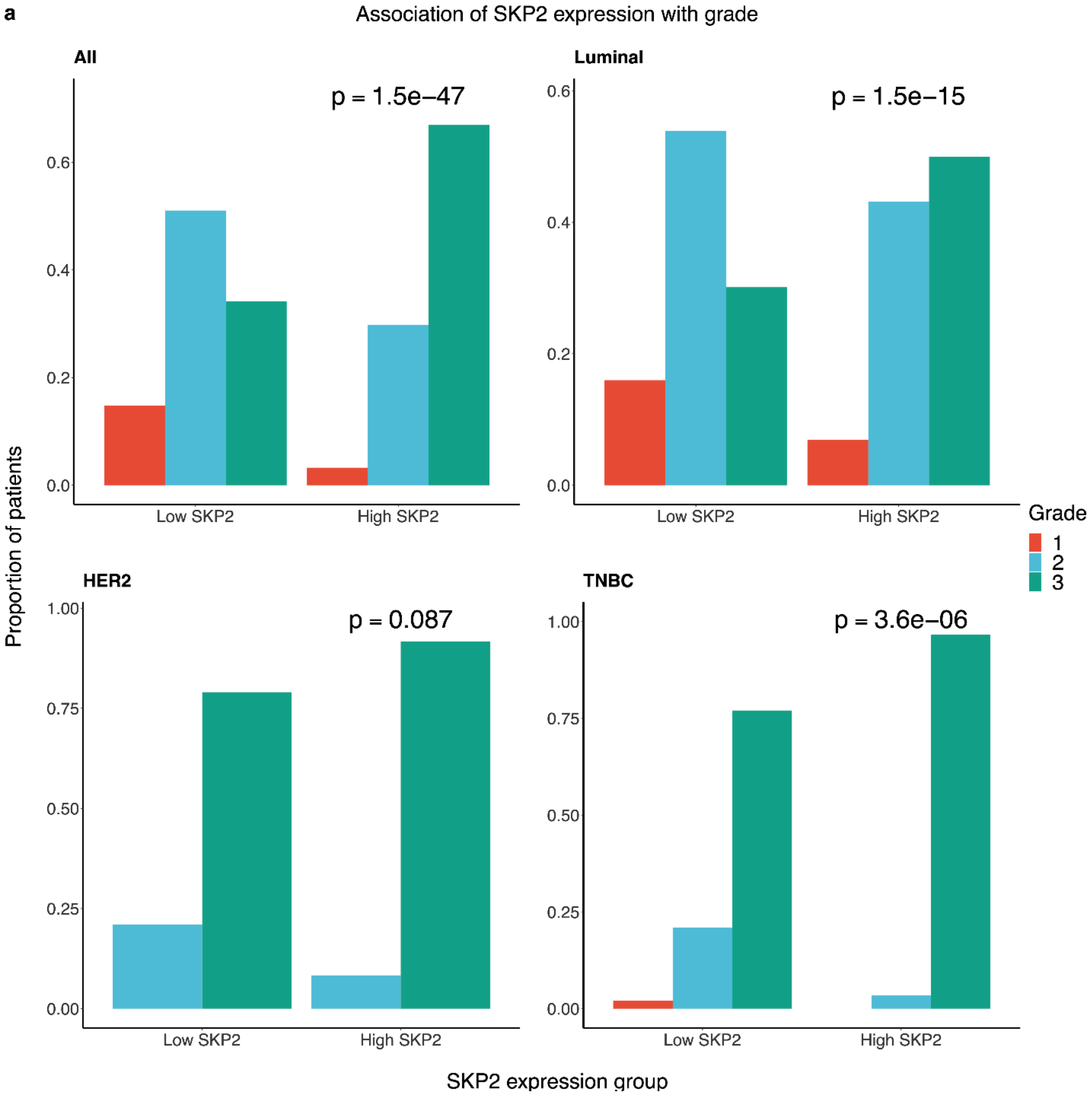

a
Association of SKP2 expression with grade
All
p = 1.5e−47
Luminal
p = 1.5e−15
HER2
p = 0.087
TNBC
p = 3.6e−06
Proportion of patients
0.0
0.2
0.4
0.6
0.00
0.25
0.50
0.75
1.00
Low SKP2
High SKP2
Grade
1
2
3
SKP2 expression group

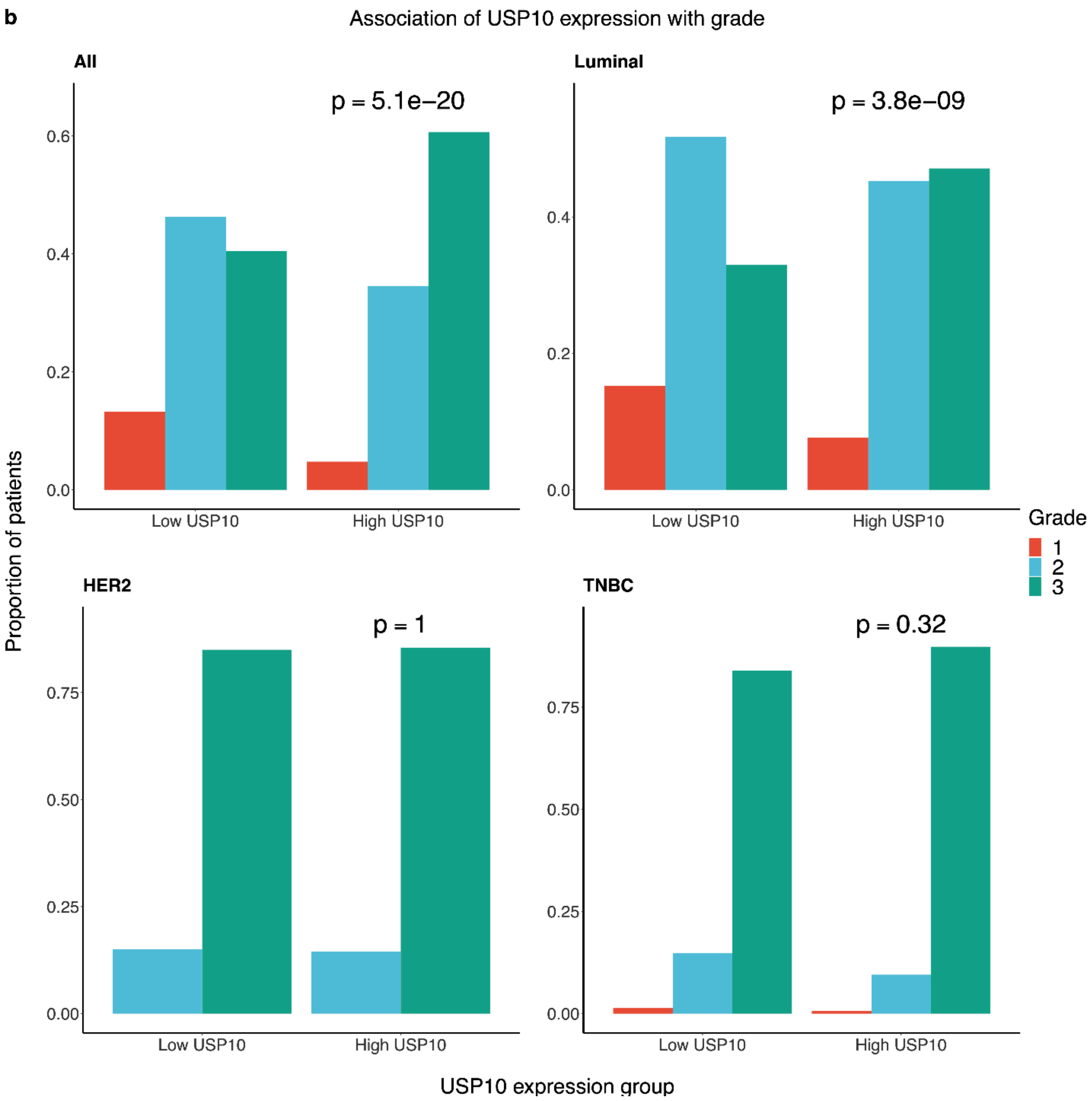
b
Association of USP10 expression with grade
All
p = 5.1e−20
Luminal
p = 3.8e−09
HER2
p = 1
TNBC
p = 0.32
Proportion of patients
0.0
0.2
0.4
0.6
0.00
0.25
0.50
0.75
Low USP10
High USP10
Grade
1
2
3
USP10 expression group

c

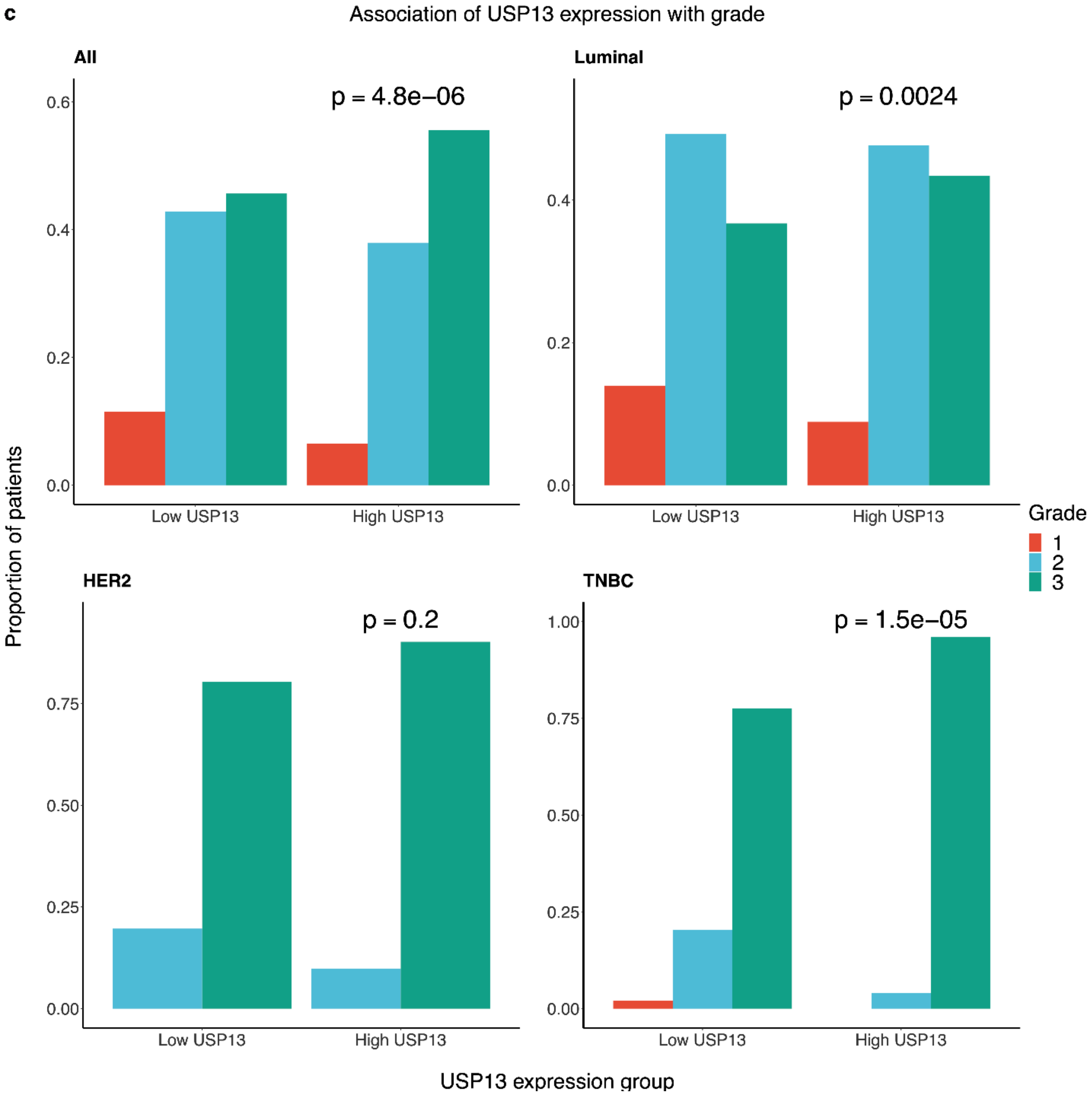
Association of USP13 expression with grade
All
p = 4.8e−06
0.6
0.4
0.2
0.0
Low USP13
High USP13
Luminal
p = 0.0024
0.4
0.2
0.0
Low USP13
High USP13
Grade
1
2
3
Proportion of patients
HER2
p = 0.2
0.75
0.50
0.25
0.00
Low USP13
High USP13
TNBC
p = 1.5e−05
1.00
0.75
0.50
0.25
0.00
Low USP13
High USP13
USP13 expression group

d

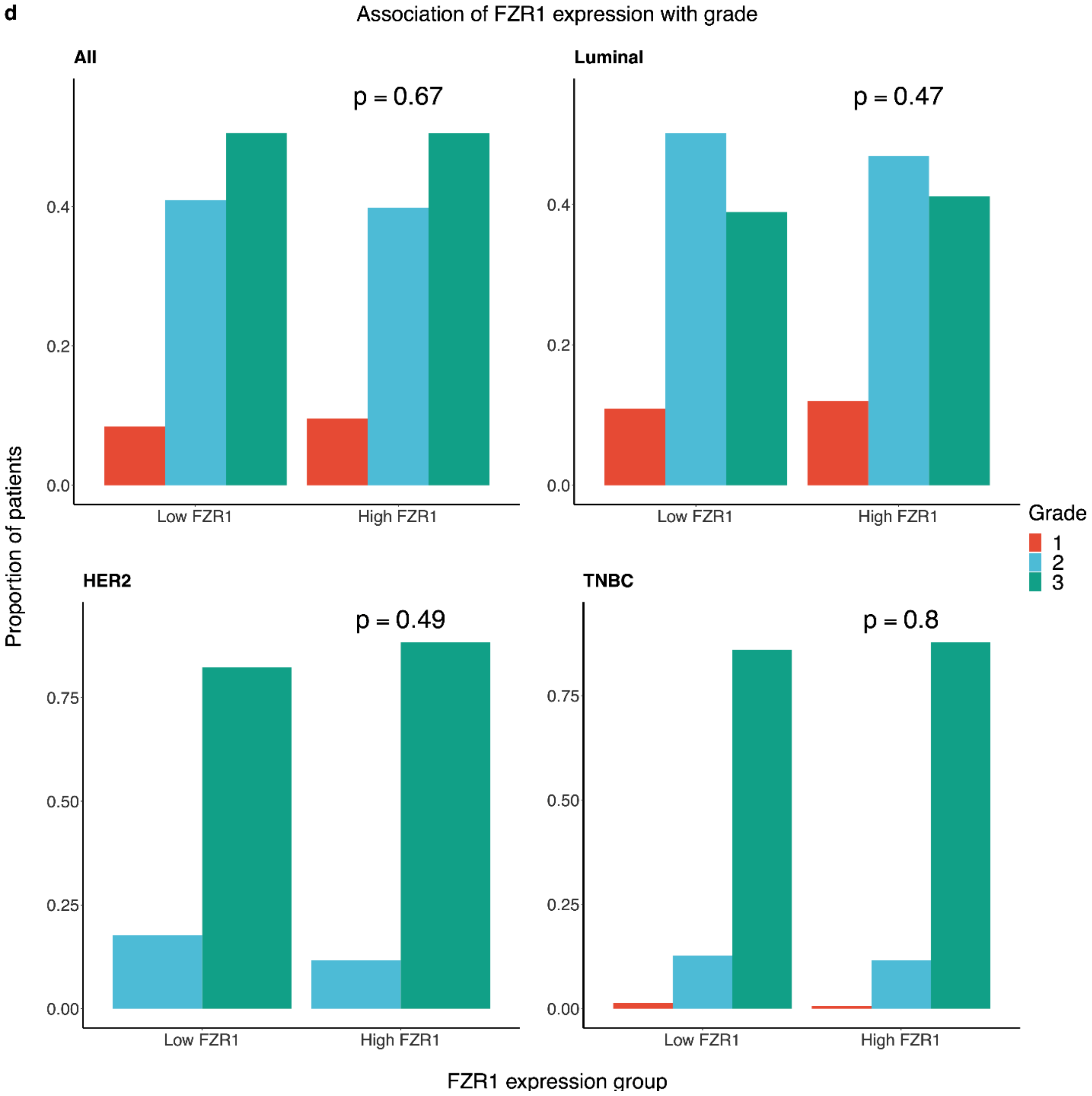

Figure S10. **The distribution of patients with a given tumor grade by expression of SKP2 (a), USP10 (b), USP13 (c), or FZR1 (d)**. Analyses were performed on the data set in aggregate (n = 1,832) and on three major subtypes separately: luminal (n = 1,414), HER2 (n = 122), and TNBC (n = 296). Subtypes are defined as in **Fig. 2**. The chi-square test was used to determine the significance of association between ubiquitination group and tumor grade. Samples were grouped by expression of the gene of interest as described in **Figure S6**.

a

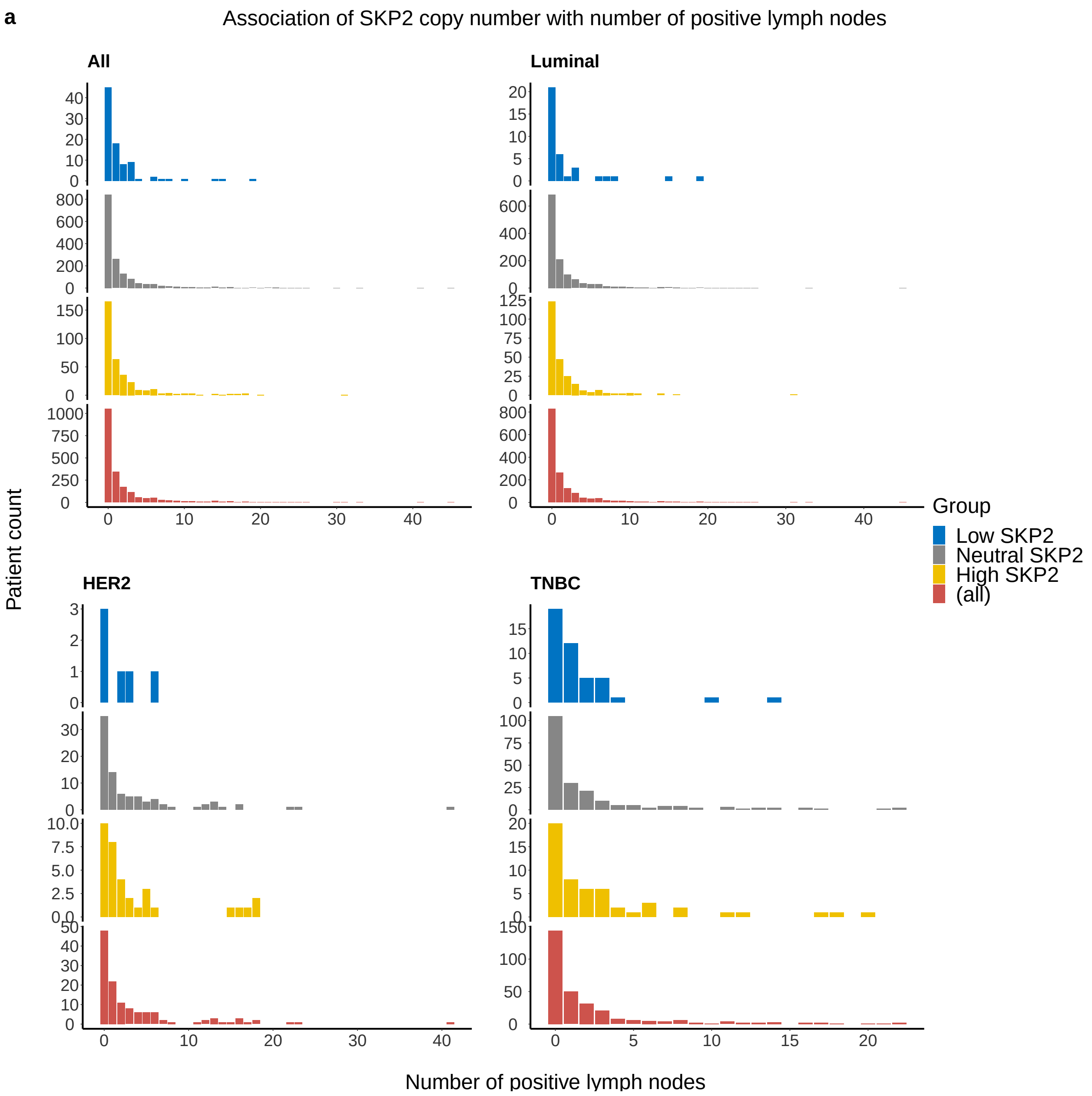
Association of SKP2 copy number with number of positive lymph nodes
All
Luminal
HER2
TNBC
Patient count
Number of positive lymph nodes
Group
Low SKP2
Neutral SKP2
High SKP2
(all)

b

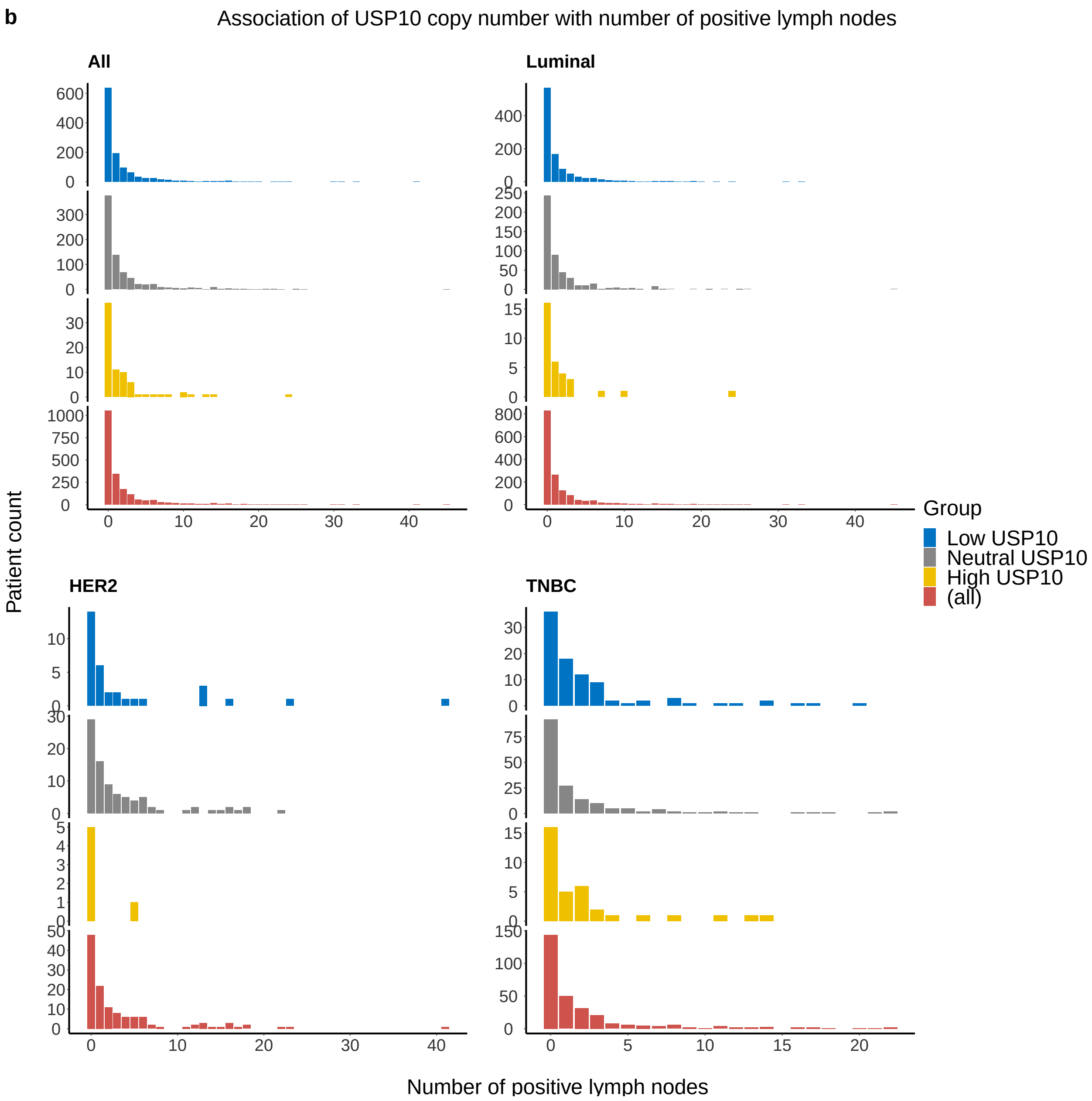
Association of USP10 copy number with number of positive lymph nodes
All
Luminal
HER2
TNBC
Patient count
Number of positive lymph nodes
Group
Low USP10
Neutral USP10
High USP10
(all)

c

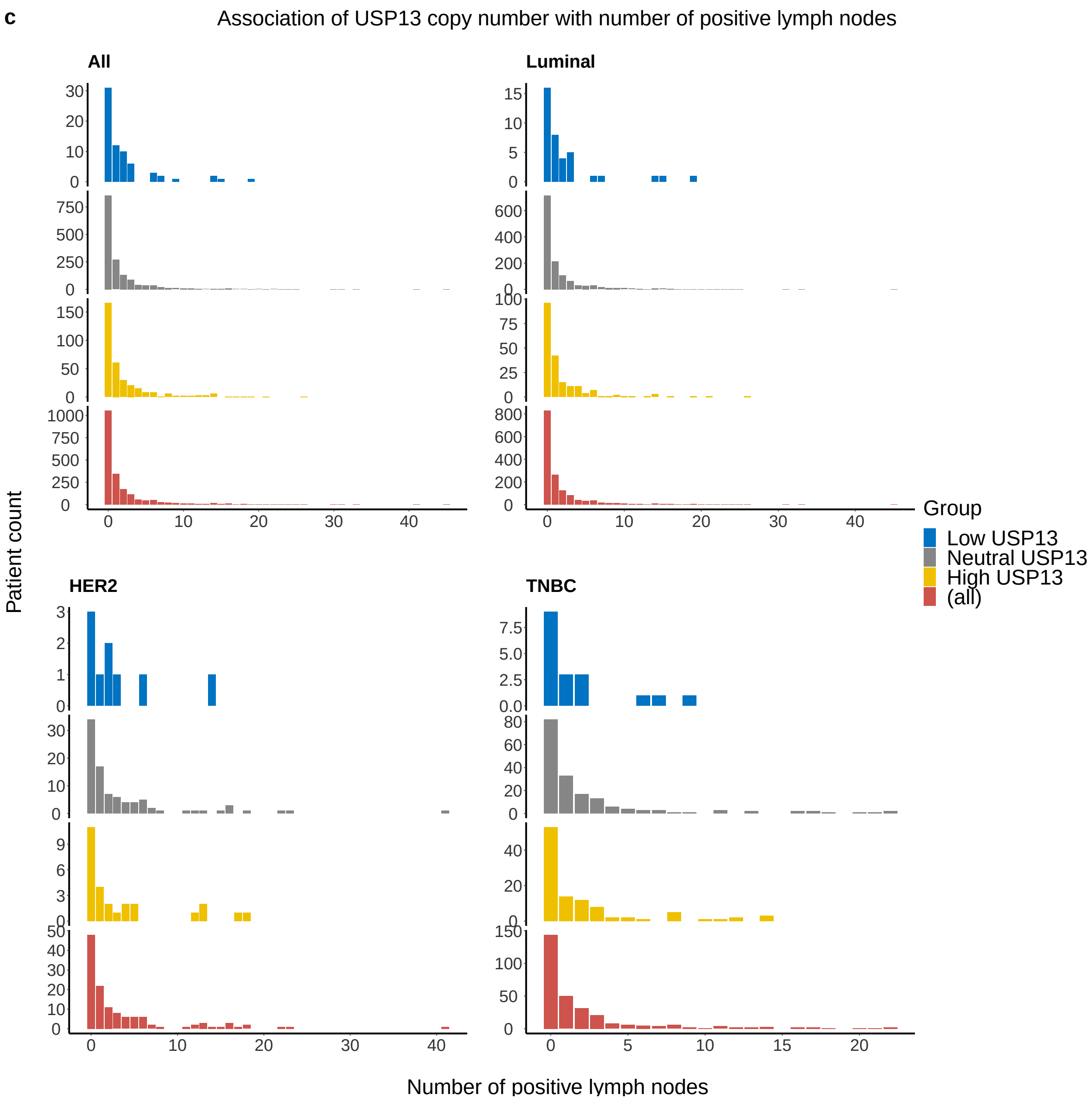
Association of USP13 copy number with number of positive lymph nodes
All
Luminal
HER2
TNBC
Patient count
Number of positive lymph nodes
Group
Low USP13
Neutral USP13
High USP13
(all)

d

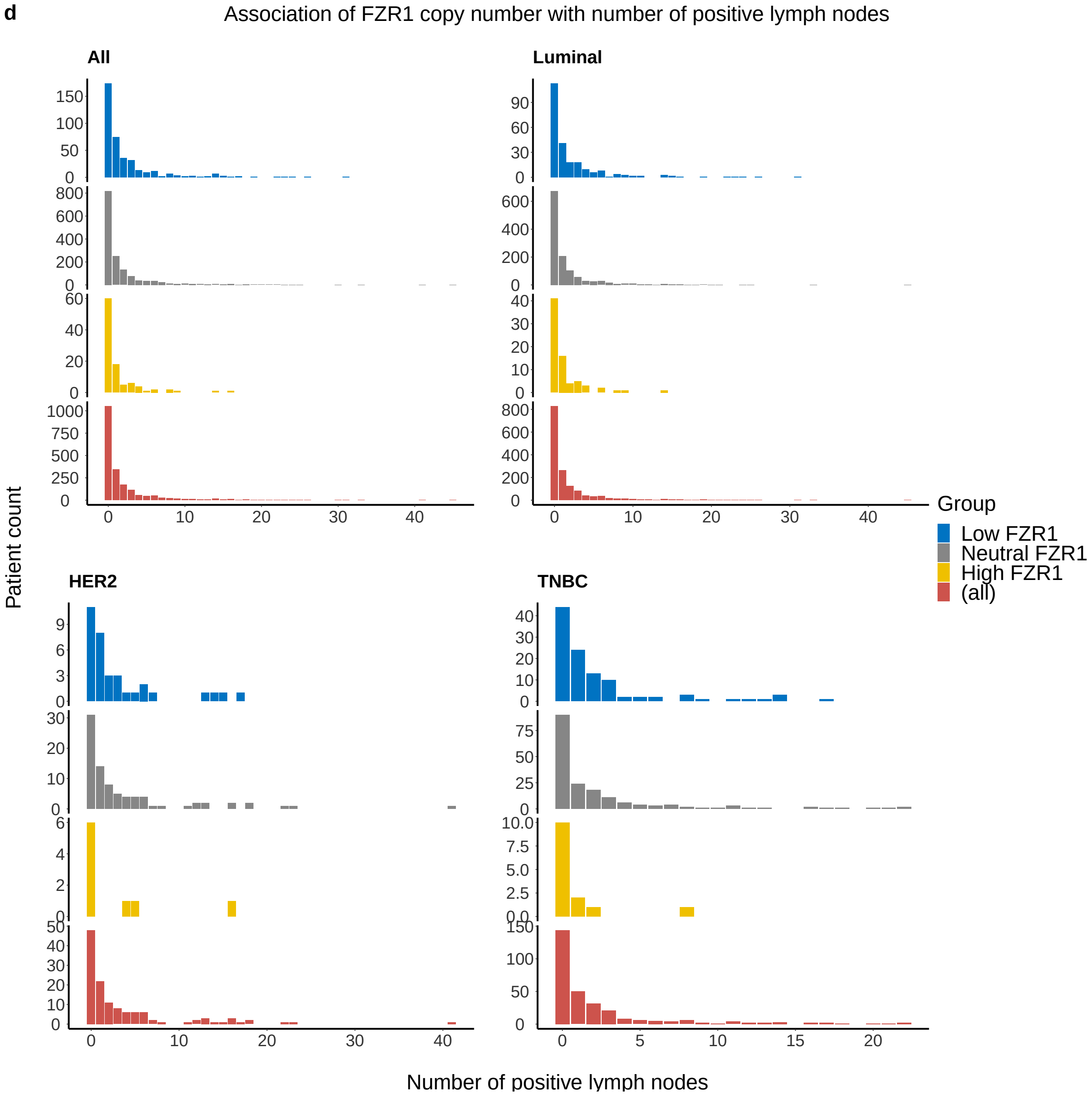

Fig. S11. Comparison of the number of positive lymph nodes per patient among the copy number (CN) groups of SKP2 (a), USP10 (b), USP13 (c), or FZR1 (d). Analyses were performed on the data set in aggregate (*n* = 2,004) and on three major subtypes separately: luminal (*n* = 1,524), HER2 (*n* = 127), and TNBC (*n* = 299). Subtypes are defined as in **Fig. 2**. Negative binomial regression was used to model count data. The means and variances of the counts of positive lymph nodes for each BC subtype is as follows: All: $\mu = 2.00$; $\sigma^2 = 16.84$. Luminal: $\mu = 1.80$; $\sigma^2 = 14.52$. HER2: $\mu = 3.70$; $\sigma^2 = 37.77$. TNBC: $\mu = 2.27$; $\sigma^2 = 16.95$. Samples were grouped by copy number–alteration levels as described in **Figure S4.** Coefficients for the negative binomial regression model fit to the count data are in **Table S9**.

a

Association of SKP2 expression with number of positive lymph nodes

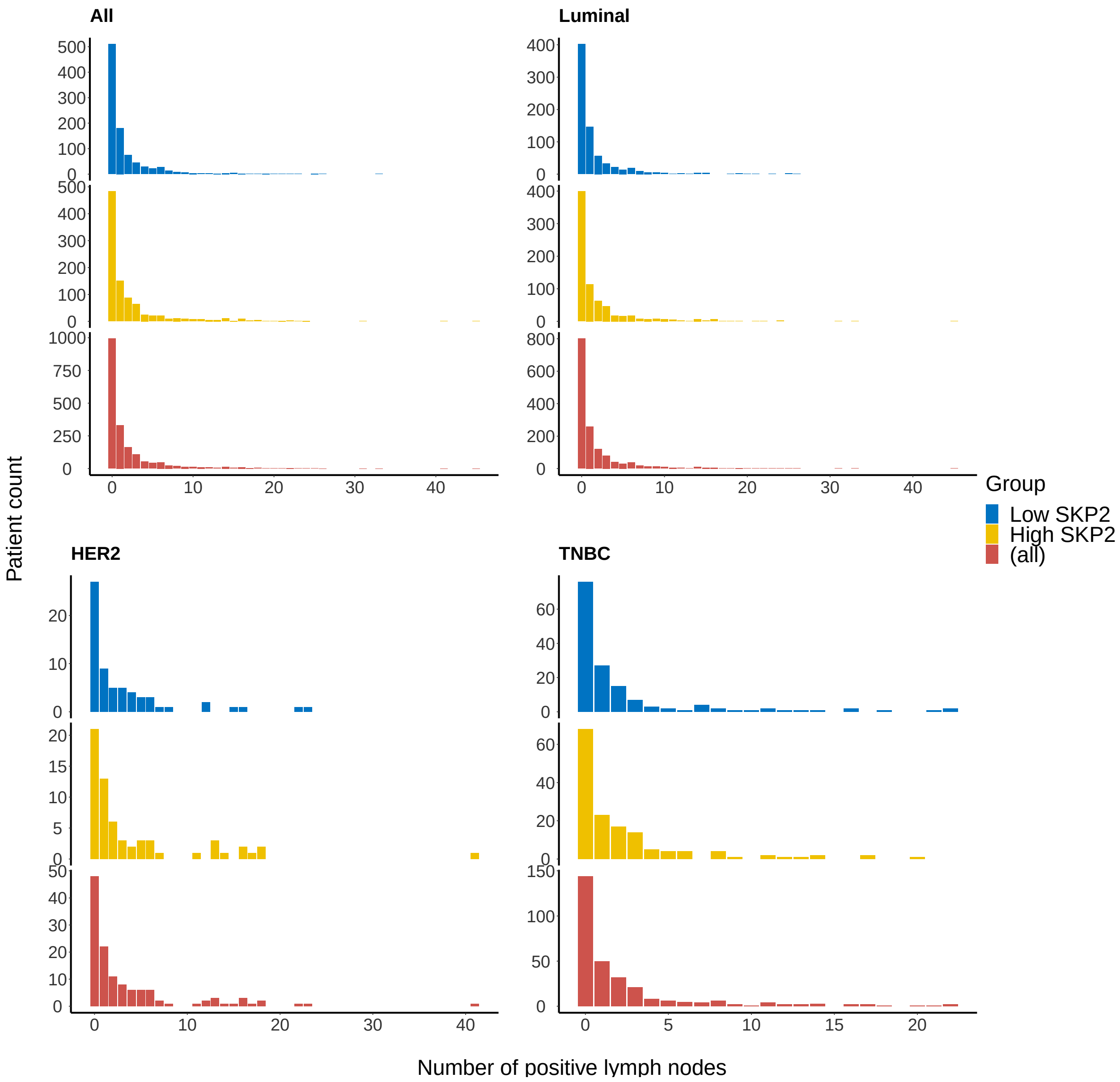

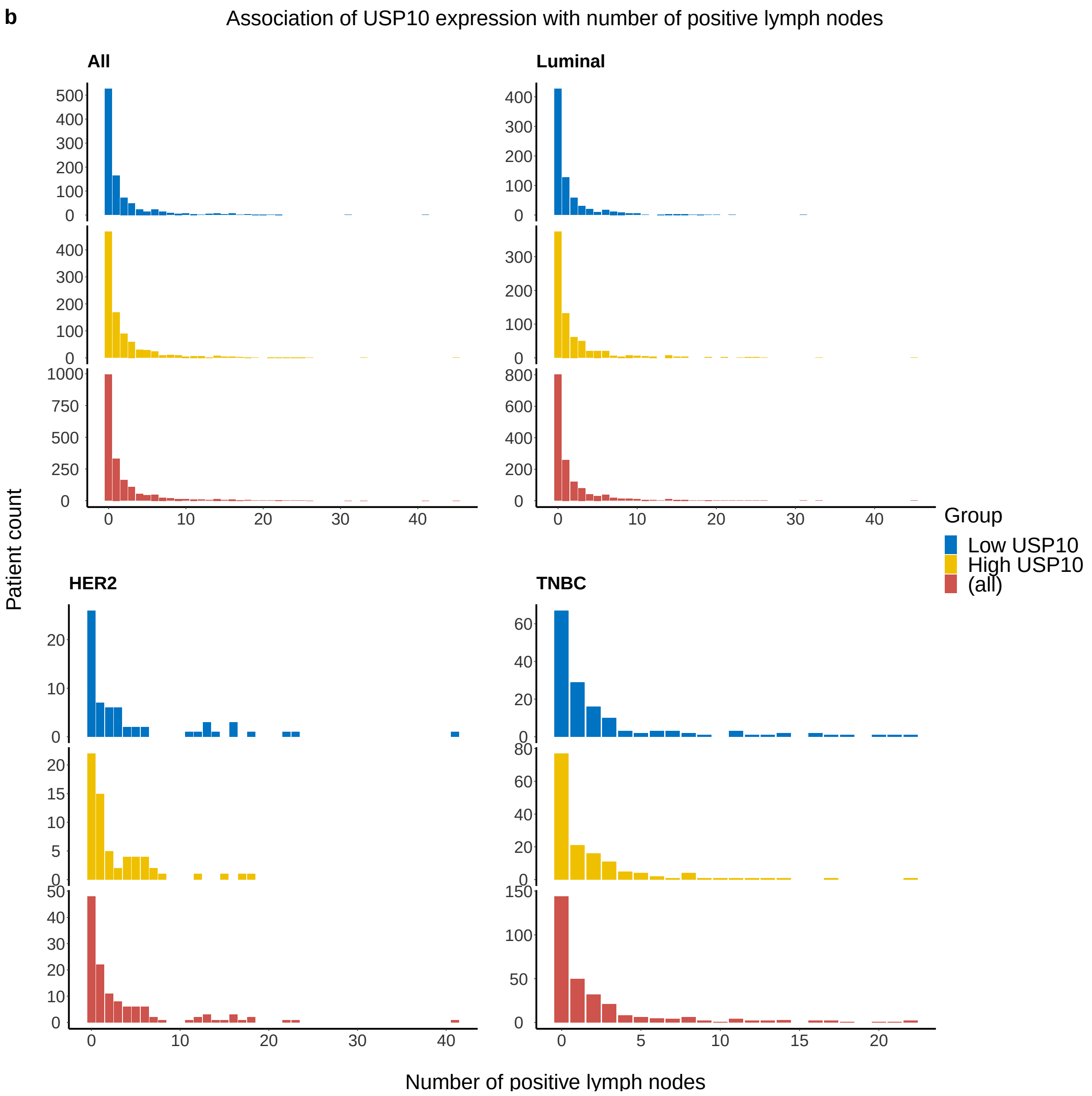
b
Association of USP10 expression with number of positive lymph nodes
All
Luminal
HER2
TNBC
Patient count
Number of positive lymph nodes
Group
Low USP10
High USP10
(all)

c

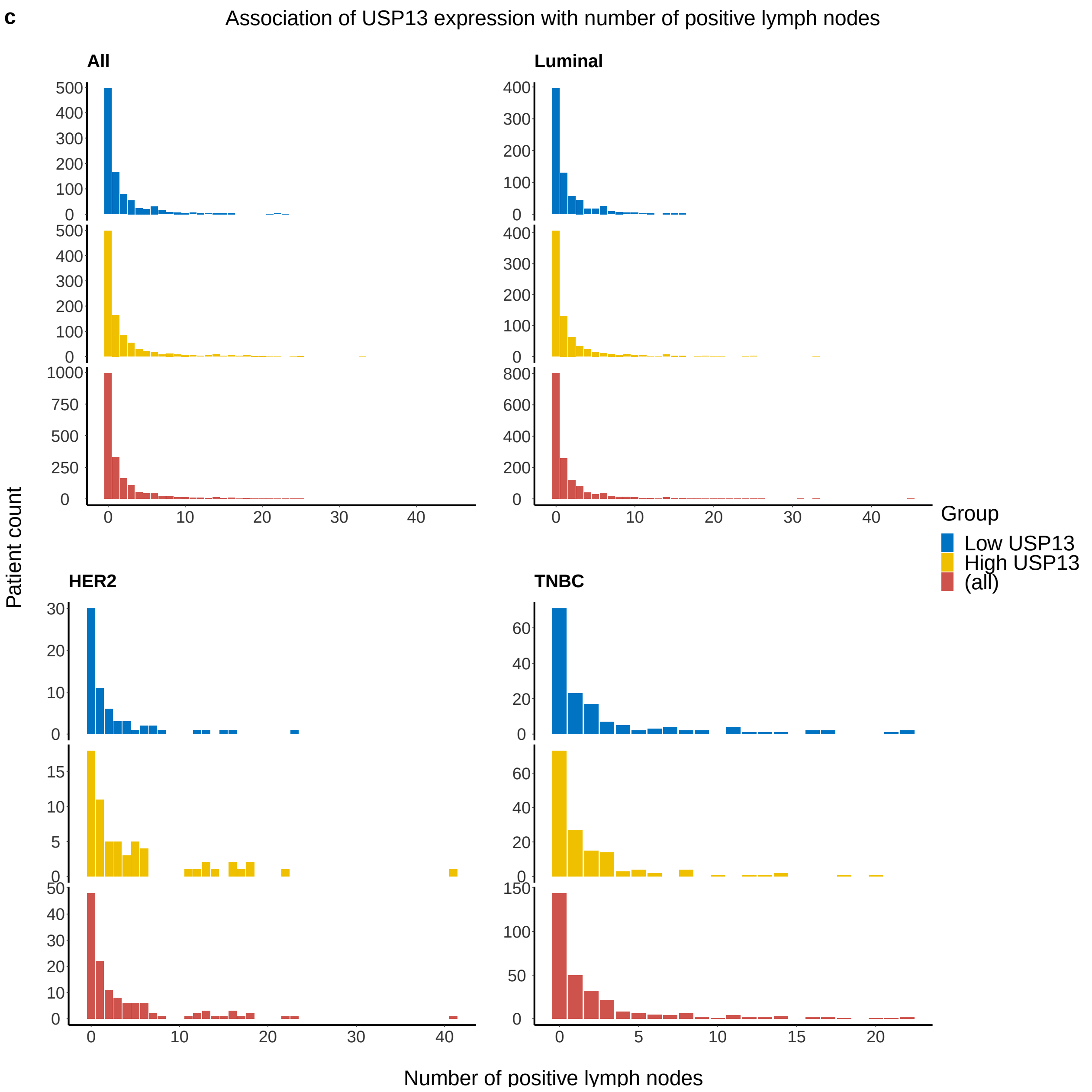

Association of USP13 expression with number of positive lymph nodes
All
Luminal
HER2
TNBC
Patient count
Number of positive lymph nodes
Group
Low USP13
High USP13
(all)

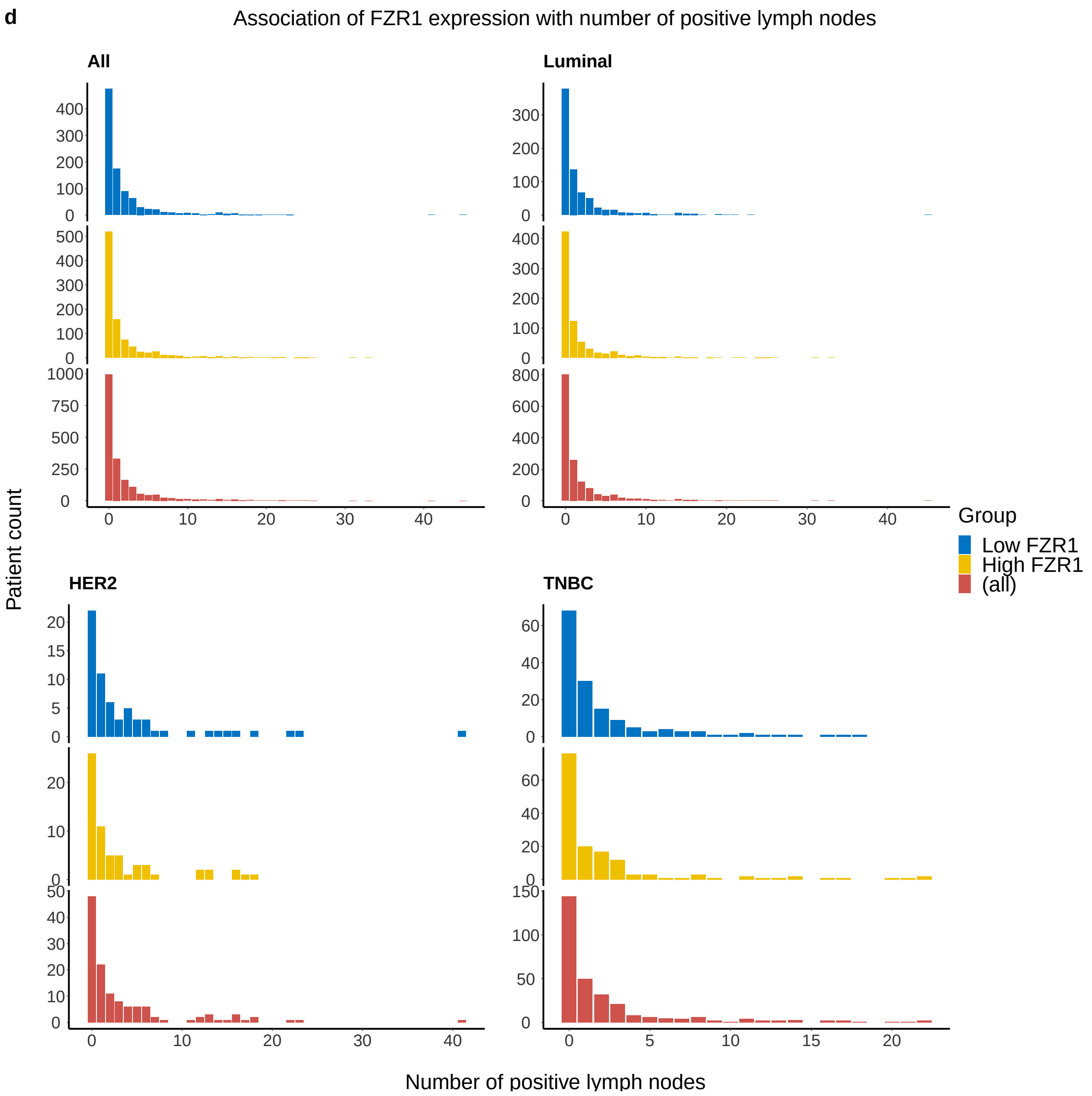

Figure S12. Comparison of the number of positive lymph nodes per patient among the expression groups of SKP2 (a), USP10 (b), USP13 (c), or FZR1 (d). Analyses were performed on the data set in aggregate (n = 1,904) and on three major subtypes separately: luminal (n = 1,478), HER2 (n = 127), and TNBC (n = 299.) Subtypes are defined as in **Fig. 2**. Negative binomial regression was used to model count data. The means and variances of the counts of positive lymph nodes for each BC subtype is as follows: All: $\mu = 2.00$; $\sigma^2 = 16.84$. Luminal: $\mu = 1.80$; $\sigma^2 = 14.52$. HER2: $\mu = 3.70$; $\sigma^2 = 37.77$. TNBC: $\mu = 2.27$; $\sigma^2 = 16.95$. Samples were grouped by expression of gene of interest as described in **Figure S6**. Coefficients for the negative binomial regression model fit to the count data are in **Table S10**.

All
USP10, FZR1

Group	Count	Mean	SD
High ubiquitination	438	0.25	0.37
Intermediate ubiquitination	289	0.22	0.40
Low ubiquitination	146	0.12	0.36

All
USP13, FZR1

Group	Count	Mean	SD
High ubiquitination	89	0.20	0.41
Intermediate ubiquitination	439	0.27	0.37
Low ubiquitination	345	0.17	0.38

All
USP14, FZR1

Group	Count	Mean	SD
High ubiquitination	191	0.19	0.37
Intermediate ubiquitination	472	0.25	0.38
Low ubiquitination	210	0.18	0.40

Luminal
USP10, FZR1

Group	Count	Mean	SD
High ubiquitination	364	0.29	0.37
Intermediate ubiquitination	182	0.27	0.41
Low ubiquitination	83	0.18	0.36

Luminal
USP13, FZR1

Group	Count	Mean	SD
High ubiquitination	64	0.27	0.42
Intermediate ubiquitination	349	0.30	0.37
Low ubiquitination	216	0.21	0.38

Luminal
USP14, FZR1

Group	Count	Mean	SD
High ubiquitination	148	0.23	0.37
Intermediate ubiquitination	357	0.29	0.38
Low ubiquitination	124	0.24	0.42

HER2
USP10, FZR1

Group	Count	Mean	SD
High ubiquitination	16	-0.0570	0.32

All
USP10, FZR1

Group	Count	Mean	SD
Intermediate ubiquitination	9	-0.0330	0.35
Low ubiquitination	11	-0.0056	0.37

HER2
USP13, FZR1

Group	Count	Mean	SD
High ubiquitination	5	-0.240	0.22
Intermediate ubiquitination	17	-0.063	0.31
Low ubiquitination	14	0.070	0.38

HER2
USP14, FZR1

Group	Count	Mean	SD
High ubiquitination	11	-0.13	0.36
Intermediate ubiquitination	13	-0.11	0.24
Low ubiquitination	12	0.13	0.36

TNBC
USP10, FZR1

Group	Count	Mean	SD
High ubiquitination	18	0.150	0.33
Intermediate ubiquitination	45	0.100	0.38
Low ubiquitination	29	0.088	0.37

TNBC
USP13, FZR1

Group	Count	Mean	SD
High ubiquitination	4	0.061	0.48
Intermediate ubiquitination	27	0.170	0.35
Low ubiquitination	61	0.085	0.37

TNBC
USP14, FZR1

Group	Count	Mean	SD
High ubiquitination	14	0.025	0.35
Intermediate ubiquitination	39	0.130	0.38
Low ubiquitination	39	0.120	0.36

Table S1. Summary statistics of p27 protein levels by ubiquitination signature group. Groups were determined as specified in **Table 1**.

All

Group	n	Events	Median	95% LCL	95% UCL
High ubiquitination	966	280	336	278	NA
Low ubiquitination	217	79	NA	NA	NA
Neutral ubiquitination	798	287	253	226	NA

Luminal

Group	n	Events	Median	95% LCL	95% UCL
High ubiquitination	886	246	336	278	NA
Low ubiquitination	114	44	NA	145	NA
Neutral ubiquitination	527	171	257	233	NA

HER2

Group	n	Events	Median	95% LCL	95% UCL
High ubiquitination	26	10	NA	39.8	NA
Low ubiquitination	26	13	49.5	34.7	NA
Neutral ubiquitination	82	43	92.5	56.3	NA

TNBC

Group	n	Events	Median	95% LCL	95% UCL
High ubiquitination	54	24	204	44.8	NA
Low ubiquitination	77	22	NA	NA	NA
Neutral ubiquitination	189	73	NA	204.2	NA

Table S2. Descriptive survival statistics for patients in the METABRIC dataset. Times reported are in months. “LCL” = “lower confidence limit”; “UCL” = “upper confidence limit.”

Group	n	Events	Median	95% LCL	95% UCL
High ubiquitination	55	6	NA	NA	NA
Low ubiquitination	102	29	NA	NA	NA
Neutral ubiquitination	141	36	14.4	12.3	NA

Table S3. Descriptive survival statistics for patients in the GSE17705 dataset. Times reported are in years. “LCL” = “lower confidence limit”; “UCL” = “upper confidence limit.”

All
USP13, FZR1

Group	Count	Mean	SD
High ubiquitination	89	0.130	0.33
Intermediate ubiquitination	439	0.094	0.34
Low ubiquitination	345	0.120	0.34

All
USP10, FZR1

Group	Count	Mean	SD
High ubiquitination	438	0.110	0.34
Intermediate ubiquitination	289	0.130	0.33
Low ubiquitination	146	0.058	0.34

All
USP14, FZR1

Group	Count	Mean	SD
High ubiquitination	191	0.150	0.33
Intermediate ubiquitination	472	0.110	0.34
Low ubiquitination	210	0.071	0.33

Luminal
USP13, FZR1

Group	Count	Mean	SD
High ubiquitination	64	0.120	0.33
Intermediate ubiquitination	349	0.093	0.33
Low ubiquitination	216	0.140	0.35

Luminal
USP10, FZR1

Group	Count	Mean	SD
High ubiquitination	364	0.110	0.34
Intermediate ubiquitination	182	0.120	0.32
Low ubiquitination	83	0.083	0.36

Luminal
USP14, FZR1

Group	Count	Mean	SD
High ubiquitination	148	0.160	0.33
Intermediate ubiquitination	357	0.099	0.34
Low ubiquitination	124	0.091	0.33

HER2
USP13, FZR1

Group	Count	Mean	SD
High ubiquitination	5	0.021	0.27
Intermediate ubiquitination	17	0.078	0.37
Low ubiquitination	14	-0.053	0.25

All
USP13, FZR1

Group	Count	Mean	SD

HER2
USP10, FZR1

Group	Count	Mean	SD
High ubiquitination	16	0.0037	0.31
Intermediate ubiquitination	9	0.1700	0.31
Low ubiquitination	11	-0.0820	0.29

HER2
USP14, FZR1

Group	Count	Mean	SD
High ubiquitination	11	0.014	0.39
Intermediate ubiquitination	13	0.180	0.24
Low ubiquitination	12	-0.150	0.22

TNBC
USP13, FZR1

Group	Count	Mean	SD
High ubiquitination	4	0.130	0.052
Intermediate ubiquitination	27	0.170	0.380
Low ubiquitination	61	0.061	0.310

TNBC
USP10, FZR1

Group	Count	Mean	SD
High ubiquitination	18	0.100	0.37
Intermediate ubiquitination	45	0.140	0.34
Low ubiquitination	29	0.025	0.28

TNBC
USP14, FZR1

Group	Count	Mean	SD
High ubiquitination	14	0.2700	0.25
Intermediate ubiquitination	39	0.1200	0.37
Low ubiquitination	39	0.0048	0.28

Table S4. Summary statistics of p21 protein levels by ubiquitination signature group. Groups were determined as specified in **Table 1**.

SKP2
All

Group	Count	Mean	SD
Shallow deletion	86	8.5	0.62
Diploid	1499	8.3	0.55
Gain	269	8.7	0.71
Amplification	50	9.2	0.84

SKP2
Luminal

Group	Count	Mean	SD
Shallow deletion	36	8.1	0.37
Diploid	1210	8.2	0.39
Gain	200	8.4	0.53
Amplification	32	8.9	0.69

SKP2
HER2

Group	Count	Mean	SD
Shallow deletion	6	8.1	0.42
Diploid	87	8.7	0.48
Gain	24	9.0	0.49
Amplification	10	9.3	0.66

SKP2
TNBC

Group	Count	Mean	SD
Shallow deletion	44	8.9	0.52
Diploid	202	9.1	0.71
Gain	45	9.6	0.68
Amplification	8	10.0	0.84

USP10
All

Group	Count	Mean	SD
Deep deletion	4	7.2	0.43
Shallow deletion	1094	7.1	0.33
Diploid	734	7.5	0.38
Gain	65	7.9	0.35
Amplification	7	8.5	0.50

USP10
Luminal

Group	Count	Mean	SD
Deep deletion	4	7.2	0.43
Shallow deletion	970	7.1	0.32
Diploid	473	7.5	0.39

SKP2
All

Group	Count	Mean	SD
Gain	28	8.0	0.33
Amplification	3	8.8	0.60

USP10
HER2

Group	Count	Mean	SD
Shallow deletion	33	7.1	0.47
Diploid	88	7.5	0.39
Gain	4	7.9	0.30
Amplification	2	8.3	0.24

USP10
TNBC

Group	Count	Mean	SD
Shallow deletion	91	7.2	0.35
Diploid	173	7.5	0.34
Gain	33	7.9	0.38
Amplification	2	8.4	0.59

USP13
All

Group	Count	Mean	SD
Deep deletion	1	5.7	NA
Shallow deletion	62	7.6	0.54
Diploid	1515	7.8	0.53
Gain	250	8.1	0.66
Amplification	76	8.6	0.64

USP13
Luminal

Group	Count	Mean	SD
Shallow deletion	36	7.6	0.50
Diploid	1247	7.8	0.52
Gain	152	8.0	0.65
Amplification	43	8.5	0.63

USP13
HER2

Group	Count	Mean	SD
Shallow deletion	9	7.3	0.62
Diploid	91	7.6	0.50
Gain	24	7.9	0.46
Amplification	3	8.3	1.30

USP13
TNBC

SKP2
All

Group	Count	Mean	SD
Group	Count	Mean	SD
Deep deletion	1	5.7	NA
Shallow deletion	17	7.8	0.52
Diploid	177	7.8	0.63
Gain	74	8.4	0.64
Amplification	30	8.8	0.53

FZR1
All

Group	Count	Mean	SD
Shallow deletion	372	5.8	0.20
Diploid	1436	5.9	0.23
Gain	85	5.9	0.21
Amplification	11	6.1	0.30

FZR1
Luminal

Group	Count	Mean	SD
Shallow deletion	230	5.8	0.21
Diploid	1175	5.9	0.22
Gain	67	5.9	0.20
Amplification	6	6.1	0.25

FZR1
HER2

Group	Count	Mean	SD
Shallow deletion	34	5.8	0.21
Diploid	84	5.9	0.29
Gain	5	5.9	0.11
Amplification	4	6.2	0.33

FZR1
TNBC

Group	Count	Mean	SD
Shallow deletion	108	5.8	0.19
Diploid	177	5.8	0.20
Gain	13	5.9	0.27
Amplification	1	5.7	NA

Table S5. Summary statistics of gene expression by copy-number group of single genes. Samples were classified according to copy-number alteration (CNA) of the gene of interest as follows: samples with CNA levels < –1: deep deletion: CNA levels = –1: shallow deletion; CNA levels = 0: diploid; CNA levels = 1: gain; CNA levels > 1: amplification. Copy-number levels were taken directly from cBioPortal, as described in the **Methods** section.

SKP2
All

Group	Count	Mean	SD
Low SKP2	89	0.07	0.30
Neutral SKP2	485	0.27	0.37
High SKP2	299	0.18	0.41
Total	873	NA	NA

SKP2
Luminal

Group	Count	Mean	SD
Low SKP2	44	0.083	0.28
Neutral SKP2	380	0.310	0.37
High SKP2	205	0.230	0.41
Total	629	NA	NA

SKP2
HER2

Group	Count	Mean	SD
Low SKP2	4	-0.160	0.12
Neutral SKP2	15	0.088	0.42
High SKP2	17	-0.110	0.25
Total	36	NA	NA

SKP2
TNBC

Group	Count	Mean	SD
Low SKP2	23	0.069	0.33
Neutral SKP2	37	0.180	0.38
High SKP2	32	0.050	0.38
Total	92	NA	NA

USP10
All

Group	Count	Mean	SD
Low USP10	546	0.26	0.39
Neutral USP10	244	0.16	0.37
High USP10	83	0.13	0.37
Total	873	NA	NA

USP10
Luminal

Group	Count	Mean	SD
Low USP10	437	0.29	0.38
Neutral USP10	146	0.22	0.39
High USP10	46	0.23	0.37
Total	629	NA	NA

SKP2
All

Group	Count	Mean	SD

USP10
HER2

Group	Count	Mean	SD
Low USP10	17	0.015	0.36
Neutral USP10	13	-0.140	0.24
High USP10	6	0.059	0.44
Total	36	NA	NA

USP10
TNBC

Group	Count	Mean	SD
Low USP10	35	0.15	0.38
Neutral USP10	36	0.11	0.35
High USP10	21	0.03	0.37
Total	92	NA	NA

USP13
All

Group	Count	Mean	SD
Low USP13	45	0.21	0.44
Neutral USP13	552	0.27	0.38
High USP13	276	0.12	0.35
Total	873	NA	NA

USP13
Luminal

Group	Count	Mean	SD
Low USP13	31	0.30	0.45
Neutral USP13	442	0.30	0.38
High USP13	156	0.17	0.36
Total	629	NA	NA

USP13
HER2

Group	Count	Mean	SD
Low USP13	4	-0.1400	0.18
Neutral USP13	18	-0.0380	0.37
High USP13	14	-0.0029	0.33
Total	36	NA	NA

USP13
TNBC

Group	Count	Mean	SD
Low USP13	1	0.360	NA

SKP2
All

Group	Count	Mean	SD
Neutral USP13	32	0.210	0.41
High USP13	59	0.046	0.34
Total	92	NA	NA

FZR1
All

Group	Count	Mean	SD
Low FZR1	265	0.18	0.40
Neutral FZR1	483	0.26	0.37
High FZR1	125	0.17	0.38
Total	873	NA	NA

FZR1
Luminal

Group	Count	Mean	SD
Low FZR1	160	0.23	0.40
Neutral FZR1	389	0.29	0.37
High FZR1	80	0.24	0.40
Total	629	NA	NA

FZR1
HER2

Group	Count	Mean	SD
Low FZR1	14	0.072	0.38
Neutral FZR1	14	-0.060	0.29
High FZR1	8	-0.180	0.30
Total	36	NA	NA

FZR1
TNBC

Group	Count	Mean	SD
Low FZR1	46	0.093	0.39
Neutral FZR1	30	0.120	0.34
High FZR1	16	0.130	0.38
Total	92	NA	NA

Table S6. Summary statistics of the protein levels by copy number (CN) of SKP2, USP10, USP13, or FZR1. Samples were grouped by copy number–alteration levels as described in **Figure S4**.

SKP2
All

Group	n	Events	Median	0.95% LCL	0.95% UCL
Low SKP2	86	30	NA	228	NA
Neutral SKP2	1566	496	286	270	NA
High SKP2	329	120	264	198	NA

SKP2
Luminal

Group	n	Events	Median	0.95% LCL	0.95% UCL
Low SKP2	36	11	NA	148	NA
Neutral SKP2	1251	376	283	270	NA
High SKP2	240	74	264	211	NA

SKP2
HER2

Group	n	Events	Median	0.95% LCL	0.95% UCL
Low SKP2	6	2	97.3	97.3	NA
Neutral SKP2	94	41	201.8	92.5	NA
High SKP2	34	23	46.2	34.7	106

SKP2
TNBC

Group	n	Events	Median	0.95% LCL	0.95% UCL
Low SKP2	44	17	NA	144.7	NA
Neutral SKP2	221	79	NA	241.6	NA
High SKP2	55	23	NA	62.9	NA

USP10
All

Group	n	Events	Median	0.95% LCL	0.95% UCL
Low USP10	1116	350	283	257	NA
Neutral USP10	792	276	286	252	NA
High USP10	73	20	NA	264	NA

USP10
Luminal

Group	n	Events	Median	0.95% LCL	0.95% UCL
Low USP10	992	292	283	278	NA
Neutral USP10	504	159	286	252	NA
High USP10	31	10	264	125	NA

USP10
HER2

Group	n	Events	Median	0.95% LCL	0.95% UCL
Low USP10	33	16	106	55.7	NA
Neutral USP10	95	48	120	51.0	NA
High USP10	6	2	NA	20.4	NA

SKP2
All

Group	n	Events	Median	0.95% LCL	0.95% UCL

USP10
TNBC

Group	n	Events	Median	0.95% LCL	0.95% UCL
Low USP10	91	42	204	126	NA
Neutral USP10	193	69	NA	242	NA
High USP10	36	8	NA	NA	NA

USP13
All

Group	n	Events	Median	0.95% LCL	0.95% UCL
Low USP13	64	23	NA	111	NA
Neutral USP13	1581	482	286	278	NA
High USP13	336	141	252	173	NA

USP13
Luminal

Group	n	Events	Median	0.95% LCL	0.95% UCL
Low USP13	37	13	NA	111	NA
Neutral USP13	1290	366	286	278	NA
High USP13	200	82	252	171	NA

USP13
HER2

Group	n	Events	Median	0.95% LCL	0.95% UCL
Low USP13	9	5	71.6	42.4	NA
Neutral USP13	96	46	146.8	51.0	NA
High USP13	29	15	120.1	62.5	NA

USP13
TNBC

Group	n	Events	Median	0.95% LCL	0.95% UCL
Low USP13	18	5	NA	159	NA
Neutral USP13	195	70	NA	228	NA
High USP13	107	44	NA	163	NA

FZR1
All

Group	n	Events	Median	0.95% LCL	0.95% UCL
Low FZR1	373	148	228	186	NA
Neutral FZR1	1509	461	286	278	NA
High FZR1	99	37	264	148	NA

FZR1
Luminal

Group	n	Events	Median	0.95% LCL	0.95% UCL
Low FZR1	231	90	221	173	NA

SKP2
All

Group	n	Events	Median	0.95% LCL	0.95% UCL
Neutral FZR1	1221	344	301	278	NA
High FZR1	75	27	264	148	NA

FZR1
HER2

Group	n	Events	Median	0.95% LCL	0.95% UCL
Low FZR1	34	19	56.3	42.7	NA
Neutral FZR1	91	43	146.8	88.9	NA
High FZR1	9	4	NA	26.7	NA

FZR1
TNBC

Group	n	Events	Median	0.95% LCL	0.95% UCL
Low FZR1	108	39	NA	227.8	NA
Neutral FZR1	197	74	NA	204.2	NA
High FZR1	15	6	NA	25.2	NA

Table S7. Descriptive survival statistics for patients in the METABRIC dataset as classified by copy-number alteration levels (see Figure S3.) Times reported are in months. “LCL” = “lower confidence limit”; “UCL” = “upper confidence limit.”

SKP2
All

Group	n	Events	Median	0.95% LCL	0.95% UCL
Low SKP2	952	231	301	278	NA
High SKP2	952	391	239	204	NA

SKP2
Luminal

Group	n	Events	Median	0.95% LCL	0.95% UCL
Low SKP2	739	161	NA	278	NA
High SKP2	739	284	253	213	NA

SKP2
HER2

Group	n	Events	Median	0.95% LCL	0.95% UCL
Low SKP2	64	30	120.1	55.6	NA
High SKP2	63	34	64.9	43.0	NA

SKP2
TNBC

Group	n	Events	Median	0.95% LCL	0.95% UCL
Low SKP2	150	55	NA	204	NA
High SKP2	149	58	NA	204	NA

USP10
All

Group	n	Events	Median	0.95% LCL	0.95% UCL
Low USP10	952	280	301	278	NA
High USP10	952	342	270	226	NA

USP10
Luminal

Group	n	Events	Median	0.95% LCL	0.95% UCL
Low USP10	739	193	301	278	NA
High USP10	739	252	264	224	NA

USP10
HER2

Group	n	Events	Median	0.95% LCL	0.95% UCL
Low USP10	64	32	120.1	56.3	NA
High USP10	63	32	97.3	42.7	NA

USP10
TNBC

Group	n	Events	Median	0.95% LCL	0.95% UCL
Low USP10	150	64	242	145	NA
High USP10	149	49	NA	NA	NA

USP13
All

Group	n	Events	Median	0.95% LCL	0.95% UCL

SKP2
All

Group	n	Events	Median	0.95% LCL	0.95% UCL
Low USP13	952	288	286	270	NA
High USP13	952	334	283	252	NA

USP13
Luminal

Group	n	Events	Median	0.95% LCL	0.95% UCL
Low USP13	739	202	286	270	NA
High USP13	739	243	278	252	NA

USP13
HER2

Group	n	Events	Median	0.95% LCL	0.95% UCL
Low USP13	64	30	146.8	56.3	NA
High USP13	63	34	88.9	44.9	NA

USP13
TNBC

Group	n	Events	Median	0.95% LCL	0.95% UCL
Low USP13	150	59	NA	163	NA
High USP13	149	54	NA	242	NA

FZR1
All

Group	n	Events	Median	0.95% LCL	0.95% UCL
Low FZR1	952	317	NA	264	NA
High FZR1	952	305	286	278	NA

FZR1
Luminal

Group	n	Events	Median	0.95% LCL	0.95% UCL
Low FZR1	739	232	NA	252	NA
High FZR1	739	213	286	278	NA

FZR1
HER2

Group	n	Events	Median	0.95% LCL	0.95% UCL
Low FZR1	64	31	120.1	56.3	NA
High FZR1	63	33	88.9	44.9	NA

FZR1
TNBC

Group	n	Events	Median	0.95% LCL	0.95% UCL
Low FZR1	150	59	NA	204	NA
High FZR1	149	54	NA	242	NA

Table S8. Descriptive survival statistics for patients in the METABRIC dataset as classified by expression levels (see Figure S6.). Times reported are in months. “LCL” = “lower confidence limit”; “UCL” = “upper confidence limit.”

SKP2
All

Factor level	Estimate	Std. error	z-value	p-value	Significance
(Intercept)	0.52	0.21	2.50	0.011	*
Neutral SKP2	0.17	0.21	0.81	0.420	
High SKP2	0.20	0.23	0.87	0.390	

SKP2
Luminal

Factor level	Estimate	Std. error	z-value	p-value	Significance
(Intercept)	0.690	0.32	2.10	0.032	*
Neutral SKP2	-0.094	0.33	-0.29	0.770	
High SKP2	-0.170	0.35	-0.49	0.620	

SKP2
HER2

Factor level	Estimate	Std. error	z-value	p-value	Significance
(Intercept)	0.61	0.70	0.86	0.39	
Neutral SKP2	0.72	0.72	1.00	0.32	
High SKP2	0.74	0.76	0.98	0.33	

SKP2
TNBC

Factor level	Estimate	Std. error	z-value	p-value	Significance
(Intercept)	0.39	0.28	1.4	0.160	
Neutral SKP2	0.41	0.31	1.3	0.180	
High SKP2	0.73	0.37	2.0	0.046	*

USP10
All

Factor level	Estimate	Std. error	z-value	p-value	Significance
(Intercept)	0.60	0.057	11.00	3.4e-26	***
Neutral USP10	0.21	0.089	2.40	1.6e-02	*
High USP10	0.15	0.230	0.67	5.0e-01	

USP10
Luminal

Factor level	Estimate	Std. error	z-value	p-value	Significance
(Intercept)	0.51	0.062	8.20	1.8e-16	***
Neutral USP10	0.23	0.110	2.10	3.4e-02	*
High USP10	0.19	0.350	0.53	5.9e-01	

USP10
HER2

Factor level	Estimate	Std. error	z-value	p-value	Significance
(Intercept)	1.50	0.28	5.40	5.5e-08	***
Neutral USP10	-0.24	0.33	-0.73	4.7e-01	

SKP2
All

Factor level	Estimate	Std. error	z-value	p-value	Significance
High USP10	-1.70	0.82	-2.10	3.8e-02	*

USP10
TNBC

Factor level	Estimate	Std. error	z-value	p-value	Significance
(Intercept)	0.93	0.19	5.00	6.1e-07	***
Neutral USP10	-0.18	0.23	-0.76	4.5e-01	
High USP10	-0.12	0.36	-0.33	7.4e-01	

USP13
All

Factor level	Estimate	Std. error	z-value	p-value	Significance
(Intercept)	0.800	0.23	3.50	0.00053	***
Neutral USP13	-0.120	0.23	-0.52	0.60000	
High USP13	-0.042	0.25	-0.17	0.87000	

USP13
Luminal

Factor level	Estimate	Std. error	z-value	p-value	Significance
(Intercept)	0.88	0.31	2.90	0.0044	**
Neutral USP13	-0.32	0.31	-1.00	0.3000	
High USP13	-0.19	0.34	-0.55	0.5800	

USP13
HER2

Factor level	Estimate	Std. error	z-value	p-value	Significance
(Intercept)	1.10	0.55	2.10	0.04	*
Neutral USP13	0.18	0.58	0.32	0.75	
High USP13	0.19	0.64	0.30	0.76	

USP13
TNBC

Factor level	Estimate	Std. error	z-value	p-value	Significance
(Intercept)	0.54	0.43	1.30	0.21	
Neutral USP13	0.35	0.45	0.78	0.43	
High USP13	0.17	0.47	0.37	0.71	

FZR1
All

Factor level	Estimate	Std. error	z-value	p-value	Significance
(Intercept)	0.88	0.096	9.2	2.3e-20	***
Neutral FZR1	-0.23	0.110	-2.1	3.6e-02	*
High FZR1	-0.60	0.220	-2.7	6.0e-03	**

FZR1
Luminal

Factor level	Estimate	Std. error	z-value	p-value	Significance

SKP2
All

Factor level	Estimate	Std. error	z-value	p-value	Significance
(Intercept)	0.91	0.12	7.4	1.7e-13	***
Neutral FZR1	-0.38	0.14	-2.8	5.5e-03	**
High FZR1	-0.67	0.26	-2.5	1.1e-02	*

FZR1
HER2

Factor level	Estimate	Std. error	z-value	p-value	Significance
(Intercept)	1.20	0.28	4.10	3.4e-05	***
Neutral FZR1	0.21	0.33	0.63	5.3e-01	
High FZR1	-0.15	0.62	-0.24	8.1e-01	

FZR1
TNBC

Factor level	Estimate	Std. error	z-value	p-value	Significance
(Intercept)	0.790	0.17	4.60	4.3e-06	***
Neutral FZR1	0.093	0.22	0.43	6.7e-01	
High FZR1	-0.940	0.56	-1.70	8.9e-02	

Table S9. Coefficients for the negative binomial regression model fit to the count data. Positive coefficients indicate a higher expected count value for that factor level (i.e., copy-number group) compared to the baseline factor level (here, low copy number.) Significance levels are indicated by asterisks as follows: “***”: $p < 0.001$, “**”: $0.001 <= p < 0.01$, “*”: $0.01 <= p < 0.05$, no text: $0.05 <= p <= 1$. Samples were grouped by copy number–alteration levels as described in **Figure S4**.

SKP2
All

Factor level	Estimate	Std. error	z-value	p-value	Significance
(Intercept)	0.54	0.062	8.7	3.3e-18	***
High SKP2	0.29	0.087	3.3	1.1e-03	**

SKP2
Luminal

Factor level	Estimate	Std. error	z-value	p-value	Significance
(Intercept)	0.48	0.072	6.7	2.5e-11	***
High SKP2	0.21	0.100	2.1	3.9e-02	*

SKP2
HER2

Factor level	Estimate	Std. error	z-value	p-value	Significance
(Intercept)	1.10	0.21	5.5	4.3e-08	***
High SKP2	0.33	0.29	1.1	2.5e-01	

SKP2
TNBC

Factor level	Estimate	Std. error	z-value	p-value	Significance
(Intercept)	0.830	0.15	5.70	1.3e-08	***
High SKP2	-0.029	0.21	-0.14	8.9e-01	

USP10
All

Factor level	Estimate	Std. error	z-value	p-value	Significance
(Intercept)	0.60	0.062	9.7	4.3e-22	***
High USP10	0.18	0.087	2.1	3.9e-02	*

USP10
Luminal

Factor level	Estimate	Std. error	z-value	p-value	Significance
(Intercept)	0.43	0.072	5.9	3.0e-09	***
High USP10	0.30	0.100	3.0	2.8e-03	**

USP10
HER2

Factor level	Estimate	Std. error	z-value	p-value	Significance
(Intercept)	1.50	0.20	7.6	2.4e-14	***
High USP10	-0.51	0.29	-1.8	7.9e-02	

USP10
TNBC

Factor level	Estimate	Std. error	z-value	p-value	Significance
(Intercept)	0.96	0.14	6.7	2.7e-11	***
High USP10	-0.31	0.21	-1.5	1.3e-01	

USP13
All

Factor level	Estimate	Std. error	z-value	p-value	Significance

SKP2
All

Factor level	Estimate	Std. error	z-value	p-value	Significance
(Intercept)	0.700	0.062	11.00	8.5e-30	***
High USP13	-0.013	0.087	-0.14	8.9e-01	

USP13
Luminal

Factor level	Estimate	Std. error	z-value	p-value	Significance
(Intercept)	0.620	0.071	8.60	6.0e-18	***
High USP13	-0.054	0.100	-0.53	5.9e-01	

USP13
HER2

Factor level	Estimate	Std. error	z-value	p-value	Significance
(Intercept)	0.93	0.21	4.5	5.9e-06	***
High USP13	0.66	0.29	2.3	2.1e-02	*

USP13
TNBC

Factor level	Estimate	Std. error	z-value	p-value	Significance
(Intercept)	0.99	0.14	6.8	7.6e-12	***
High USP13	-0.37	0.21	-1.8	7.4e-02	

FZR1
All

Factor level	Estimate	Std. error	z-value	p-value	Significance
(Intercept)	0.7000	0.062	11.000	2.1e-29	***
High FZR1	-0.0031	0.087	-0.036	9.7e-01	

FZR1
Luminal

Factor level	Estimate	Std. error	z-value	p-value	Significance
(Intercept)	0.600	0.071	8.40	3.2e-17	***
High FZR1	-0.027	0.100	-0.27	7.9e-01	

FZR1
HER2

Factor level	Estimate	Std. error	z-value	p-value	Significance
(Intercept)	1.50	0.20	7.1	8.9e-13	***
High FZR1	-0.31	0.29	-1.1	2.9e-01	

FZR1
TNBC

Factor level	Estimate	Std. error	z-value	p-value	Significance
(Intercept)	0.76	0.15	5.20	2.1e-07	***
High FZR1	0.11	0.21	0.52	6.1e-01	

Table S10. Coefficients for the negative binomial regression model fit to the count data. Positive coefficients indicate a higher expected count value for that factor level (i.e., expression group) compared to the baseline factor level (here, low expression.) Significance levels are indicated by asterisks as follows: "***": $p < 0.001$, "**": $0.001 <= p < 0.01$, "*": $0.01 <= p < 0.05$, no text: $0.05 <= p <= 1$. Samples were grouped by expression levels as described in **Figure S6**.

Supplementary References:

1. Michnick, S. W. The connectivity map. *Nat. Chem. Biol.* **2**, 663–664 (2006).
2. Xiao, Y. *et al.* Gene Perturbation Atlas (GPA): a single-gene perturbation repository for characterizing functional mechanisms of coding and non-coding genes. *Sci. Rep.* **5**, 10889 (2015).
3. Chan, C.-H. *et al.* Pharmacological inactivation of Skp2 SCF ubiquitin ligase restricts cancer stem cell traits and cancer progression. *Cell* **154**, (2013).
4. Wang, G. *et al.* Her2 promotes early dissemination of breast cancer by suppressing the p38 pathway through Skp2-mediated proteasomal degradation of Tpl2. *Oncogene* **39**, 7034–7050 (2020).
